# Burden of disease attributable to risk factors in European countries: a scoping literature review

**DOI:** 10.1186/s13690-023-01119-x

**Published:** 2023-06-25

**Authors:** Vanessa Gorasso, Joana Nazaré Morgado, Periklis Charalampous, Sara M. Pires, Juanita A. Haagsma, João Vasco Santos, Jane Idavain, Che Henry Ngwa, Isabel Noguer, Alicia Padron-Monedero, Rodrigo Sarmiento, Vera Pinheiro, Elena Von der Lippe, Lea Sletting Jakobsen, Brecht Devleesschauwer, Dietrich Plass, The COST Action CA18218 participants, Gunn Marit Aasvang, Balázs Ádám, Ala’a Alkerwi, Jalal Arabloo, Ana Lúcia Baltazar, Hilal Bektas Uysal, Boris Bikbov, Anette Kocbach Bolling, Maria Borrell-Pages, Giulia Carreras, Giulio Castelpietra, José Chen-Xu, Šeila Cilović Lagarija, Barbara Corso, Sarah Cuschieri, Robby De Pauw, Sonia Dhaouadi, Klara Dokova, Keren Dopelt, Mary Economou, Theophilus I. Emeto, Peter Fantke, Florian Fischer, Alberto Freitas, Lucia Galluzzo, Juan Manuel García-González, Federica Gazzelloni, Mika Gissler, Artemis Gkitakou, Sezgin Gubes, Irina Guseva Canu, Cesar A. Hincapié, Paul Hynds, Irena Ilic, Milena Ilic, Gaetano Isola, Zubair Kabir, Pavel Kolkhir, Naime Meriç Konar, Mirjam Kretzschmar, Mukhtar Kulimbet, Carlo La Vecchia, Carina Ladeira, Brian Lassen, Paolo Lauriola, Heli Lehtomäki, Miriam Levi, Marjeta Majer, Scott A. McDonald, Enkeleint A. Mechili, Janis Misins, Lorenzo Monasta, Javier Muñoz Laguna, Sónia Namorado, Evangelia Nena, Edmond S. W. Ng, Paul Nguewa, Vikram Niranjan, Iskra Alexandra Nola, Marija Obradović, Rónán O’Caoimh, Nazife Öztürk, M. Ramiro Pastorinho, Panagiotis Petrou, Mariana Peyroteo, Miguel Reina Ortiz, Silvia Riva, João Rocha-Gomes, Cornelia Melinda Adi Santoso, Tugce Schmitt, Rajesh Shigdel, Rannveig Sigurvinsdottir, Joan B. Soriano, Ana Catarina Sousa, Maximilian Sprügel, Paschalis Steiropoulos, Fimka Tozija, Brigid Unim, Bram Vandeninden, Orsolya Varga, Milena Vasic, Susana Viegas, Rafael Vieira, Francesco S. Violante, Grant M. A. Wyper, Vahit Yigit, Jelka Zaletel

**Affiliations:** 1 Department of Epidemiology and Public Health, Sciensano, Brussels, Belgium; 2 Department of Public Health and Primary Care, Ghent University, Ghent, Belgium; 3 Environmental Health and Nutrition Laboratory, University of Lisbon, Lisbon, Portugal; 4 Department of Public Health, Erasmus MC, Rotterdam, The Netherlands; 5 National Food Institute, Technical University of Denmark, Lyngby, Denmark; 6 MEDCIDS - Department of Community Medicine, Information and Health Decision Sciences, University of Porto, Porto, Portugal; 7 CINTESIS, Centre for Health Technology and Services Research, Porto, Portugal; 8 Public Health Unit, ACES Grande Porto V - Porto Ocidental, Porto, Portugal; 9 Department of Health Statistics, National Institute for Health Development, Tallinn, Estonia; 10 School of Public Health and Community Medicine, Sahlgrenska Academy, Gothenburg, Sweden; 11 Department of Epidemiology and Population Health, American University of Beirut, Beirut, Lebanon; 12 Carlos III Institute of Health, National School of Public Health, Madrid, Spain; 13 Medicine School, University of Applied and Environmental Sciences, Bogota, Colombia; 14 Public Health Unit, Matosinhos Local Health Unit, Matosinhos, Portugal; 15 Department of Epidemiology and Health Monitoring, Robert Koch Institute, Berlin, Germany; 16 Division for Diet, Disease Prevention and Toxicology, Technical University of Denmark, Lyngby, Denmark; 17 Department of Translational Physiology, Infectiology and Public Health, Ghent University, Merelbeke, Belgium; 18 Department for Exposure Assessment and Environmental Health Indicators, German Environment Agency, Berlin, Germany

**Keywords:** Burden of disease, Comparative risk assessment, Risk factors

## Abstract

**Objectives:**

Within the framework of the burden of disease (BoD) approach, disease and injury burden estimates attributable to risk factors are a useful guide for policy formulation and priority setting in disease prevention. Considering the important differences in methods, and their impact on burden estimates, we conducted a scoping literature review to: (1) map the BoD assessments including risk factors performed across Europe; and (2) identify the methodological choices in comparative risk assessment (CRA) and risk assessment methods.

**Methods:**

We searched multiple literature databases, including grey literature websites and targeted public health agencies websites.

**Results:**

A total of 113 studies were included in the synthesis and further divided into independent BoD assessments (54 studies) and studies linked to the Global Burden of Disease (59 papers). Our results showed that the methods used to perform CRA varied substantially across independent European BoD studies. While there were some methodological choices that were more common than others, we did not observe patterns in terms of country, year or risk factor. Each methodological choice can affect the comparability of estimates between and within countries and/or risk factors, since they might significantly influence the quantification of the attributable burden. From our analysis we observed that the use of CRA was less common for some types of risk factors and outcomes. These included environmental and occupational risk factors, which are more likely to use bottom-up approaches for health outcomes where disease envelopes may not be available.

**Conclusions:**

Our review also highlighted misreporting, the lack of uncertainty analysis and the under-investigation of causal relationships in BoD studies. Development and use of guidelines for performing and reporting BoD studies will help understand differences, avoid misinterpretations thus improving comparability among estimates.

**Registration:**

The study protocol has been registered on PROSPERO, CRD42020177477 (available at: https://www.crd.york.ac.uk/PROSPERO/).

**Supplementary Information:**

The online version contains supplementary material available at 10.1186/s13690-023-01119-x.

## Background

Since the publication of the first Global Burden of Disease (GBD) study, the burden of disease (BoD) approach has been widely applied to identify the comparative population health impact of risk factors and their disease outcomes across different populations. In particular, the BoD approach utilises Disability-Adjusted Life Years (DALY): a summary measure of population health merging into a single figure a mortality component, expressed in Years of Life Lost (YLL), and morbidity component, expressed in Years Lived with Disability (YLD) [1]. Disease and injury burden estimates attributable to risk factors are a useful guide for policy formulation and priority setting in prevention, since many risk factors are linked to individual behaviours or environmental factors that can be modified.

Two major approaches are used for the evaluation of risk factor assessment: top-down and bottom-up approach, which are mainly distinguished by the use of the Population Attributable Fraction (PAF) and could produce substantially different estimates for the same factor. Risk factor assessment as used in the GBD studies uses the Comparative Risk Assessment (CRA) framework to estimate the fraction of disease burden in a population that can be avoided if exposure to a given risk factor was removed or reduced to an ideal scenario, using PAF [2, 3]. The PAF is calculated using relative risks (RR) and quantitative information on the exposure to the risk factor in a specific population. CRA is referred to as a top-down approach, where the currently observed distribution of exposure is compared with an exposure where the risk to develop health complaints is at a minimum level, the so-called Theoretical Minimum Risk Exposure Level (TMREL). The latter can take many forms such as the lowest observed exposure or the full absence of the risk factor [2, 3]. However, attributable burden can be estimated using other approaches than CRA but remaining within the risk assessment paradigm. This is considered a bottom-up approach where the potential adverse health effects associated with exposure to a risk factor are estimated without estimating a PAF. An important difference with CRA is the absence of the comparison with a TMREL and the lack of a disease envelope, meaning the absence of an estimation of the total burden of a specific disease. In the CRA approach, the disease envelope (or total burden) would be multiplied with the PAF of a risk factor to estimate the burden attributable to that risk factor. The absence of a disease envelope might result in “unrealistic” estimations since the total disease envelope is not considered.

In general, the CRA framework offers a useful approach for synthesising evidence on risk factors as well as risk-outcome associations. The CRA methodology has been applied in several sub-national and national studies, but with adaptations to their contexts and the use of methodological choices and assumptions selected for different settings, risk factors and populations.

Previous mapping activities of BoD assessments performed across the European Region showed that methodological design choices and model parameters for assessing the BoD and/or injuries are not harmonized [4-7]. WHO/ILO collaborators conducted a systematic review of the work-related BoD and injuries. It highlighted the importance of risk of bias, quality of evidence and strength of evidence in BoD studies [8]. However, none of these systematic literature reviews mapped specific methodological design choices that have been used in BoD studies assessing the BoD attributable to risk factors. Therefore, we aimed to systematically review BoD assessing the burden attributable to risk factor across Europe and assess their methodological choices when using the CRA approach. The following key questions were addressed:

i. How many BoD assessments including risk factors have been performed across Europe, and which risk factors were considered?
ii. Which BoD methodological choices have been used in these studies?
iii. Are there any patterns of these BoD methodological choices by country, year, or risk factor studied?

## Materials and methods

The scoping literature review was part of a series of literature reviews launched by the COST Action CA18218 European Burden of Disease Network (burden-eu) [9]*.* This literature review was conducted following the guideline produced by the Centre for Reviews and Dissemination (CRD) [10]. The study protocol has been registered on PROSPERO, CRD42020177477 (available at: https://www.crd.york.ac.uk/PROSPERO/).

### Data sources and search strategy

We systematically searched Medline, Embase, Cochrane Library, Google Scholar, and Web of Science, using search terms covering CRA and calculations of BoD attributable to risk factors. The search strategy was developed after consultation with an experienced librarian from the Erasmus MC, The Netherlands, in April 2020. The search strings are provided in Additional file 1. A grey literature search was also carried out including (1) grey literature websites (i.e., OpenGrey, OAIster, CABDirect, and World Health Organization) and (2) websites of public health agencies (see Additional file 2). Only formal reports were included in our review. Further, the reference lists of identified systematic reviews were screened for eligible studies. Additionally, *burden-eu* members were asked to contribute to the final list of publications with any additional literature available in their own countries. We did not apply a restriction by language. Since the DALY concept was introduced in the 1993 World Development Report [11], we screened BoD studies published between January 1990 and April 2020.

### Inclusion and exclusion criteria

Inclusion and exclusion criteria are summarized in the PCOS-T below (Table 1). We included studies that assessed the BoD attributable to risk factors in terms of YLL, YLD, or DALY conducted within the GBD European Region (45 countries from Western, Central and Eastern Europe, see Supporting files). Global studies were also included provided that they also included data for the European countries of interest. We defined as risk factor every individual behavioural choice or environmental, metabolic, occupational factor that affects the risk associated to a disease outcome. This included some diseases regarded as risk factors, for example, type 2 diabetes is associated with an increased risk of some cancers. We excluded BoD studies that did not assess the impact of risk factors but only focused on diseases or injuries. We also excluded studies that included indicators/health metrics other than YLL, YLD and/or DALY (e.g. computation of potential years of life lost, estimation of disability weights), as well as books, theses, conference proceedings, editorials, and letters-to-editor.

**Table 1:** PCOS-T table summarizing inclusion and exclusion criteria

	**Inclusion**	**Exclusion**
Population	GBD European region countries (45 countries from Western, Central and Eastern Europe)	Non- GBD European region countries
Outcomes	Burden of disease methodology and calculations information related to comparative risk assessments and attributable burden	Non burden of disease outcomes; definition of “years of life lost” different from the one indicated in Murray (1994) [12]
Study design	National and local burden of disease studies, global burden of disease studies	Non-burden of disease studies, non-observational studies (e.g. clinical trials or interventional studies); publications not including methodological information (e.g. conference abstracts)
Time frame	1990 to present	Before 1990 (DALYs got introduced in the 1990s, before there were already several studies on “years of life lost”)

### Screening and data extraction

The records were screened using Rayyan [13] and imported into an Excel spreadsheet. Two researchers (VG and JM) independently screened the publications. The decision to include a paper was based on title, abstract, and full-text screening. All queries were discussed by the reviewers and any outstanding queries resolved by DP. Data extraction was performed independently by VG and JM using an Excel spreadsheet. The extraction items were previously discussed by a larger group of collaborators (DP, BD, VG, JM, PC, JH, SMP, EVdL) and were piloted in previous systematic literature reviews [5, 7]. Definitions of these items can be found in the Additional file 3.

### Data synthesis

For the data synthesis, studies were classified according to: (i) type of risk factor analysed, based on the different levels defined by GBD (see Table 2) and (ii) type of study (independent *versus* GBD-linked study). The term ‘independent BoD study’ refers to single-country or multi-country studies for which researchers performed their own calculations and analyses of YLL, YLD and/or DALY attributable to risk factors. The term ‘GBD-linked study’ refers to single-country or multi-country studies in which the BoD attributable to risk factors was derived from the existing GBD study estimates [14, 15] (i.e. Institute for Health Metrics and Evaluation or WHO Global Health Estimates). Our review focused on the summary of the methodological choices of independent BoD risk factor studies. GBD-linked studies were excluded from the summary because they share the same methodological design choices and thus, their inclusion would bias the results. In order to present an accurate mapping of the studies estimating risk factor attributable burden in Europe, we included GBD-linked studies in the initial descriptive analysis and on the reference lists that can be found in the Additional file 5.

**Table 2:** Risk factor hierarchical categorization as defined by GBD study 2019 [14]

**Level**	**Risk factor**
Level 1	Environmental/occupational risks
Level 2	Unsafe water, sanitation, and handwashing
Level 3	Unsafe water source
Level 3	Unsafe sanitation
Level 3	No access; handwashing facility
Level 2	Air pollution
Level 3	Particulate matter pollution
Level 4	Ambient particulate matter pollution
Level 4	Household air pollution from solid fuels
Level 3	Ambient ozone pollution
Level 2	Non-optimal temperature
Level 3	High temperature
Level 3	Low temperature
Level 2	Other environmental risks
Level 3	Residential radon
Level 3	Lead exposure
Level 2	Occupational risks
Level 3	Occupational carcinogens
Level 4	Occupational exposure; asbestos
Level 4	Occupational exposure; arsenic
Level 4	Occupational exposure; benzene
Level 4	Occupational exposure; beryllium
Level 4	Occupational exposure; cadmium
Level 4	Occupational exposure; chromium
Level 4	Occupational exposure; diesel engine exhaust
Level 4	Occupational exposure; formaldehyde
Level 4	Occupational exposure; nickel
Level 4	Occupational exposure; polycyclic aromatic hydrocarbons
Level 4	Occupational exposure; silica
Level 4	Occupational exposure; sulfuric acid
Level 4	Occupational exposure; trichloroethylene
Level 3	Occupational asthmagens
Level 3	Occupational particulate matter, gases, and fumes
Level 3	Occupational noise
Level 3	Occupational injuries
Level 3	Occupational ergonomic factors
Level 1	Behavioral risks
Level 2	Child and maternal malnutrition
Level 3	Suboptimal breastfeeding
Level 4	Non-exclusive breastfeeding
Level 4	Discontinued breastfeeding
Level 3	Child growth failure
Level 4	Child underweight
Level 4	Child wasting
Level 4	Child stunting
Level 3	Low birth weight and short gestation
Level 4	Low birth weight
Level 4	Short gestation
Level 3	Iron deficiency
Level 3	Vitamin A deficiency
Level 3	Zinc deficiency
Level 2	Tobacco
Level 3	Smoking
Level 3	Chewing tobacco
Level 3	Secondhand smoke
Level 2	Alcohol use
Level 2	Drug use
Level 2	Dietary risks
Level 3	Diet low in fruits
Level 3	Diet low in vegetables
Level 3	Diet low in legumes
Level 3	Diet low in whole grains
Level 3	Diet low in nuts and seeds
Level 3	Diet low in milk
Level 3	Diet high in red meat
Level 3	Diet high in processed meat
Level 3	Diet high in sugar-sweetened beverages
Level 3	Diet low in fiber
Level 3	Diet low in calcium
Level 3	Diet low in seafood omega-3 fatty acids
Level 3	Diet low in polyunsaturated fatty acids
Level 3	Diet high in trans fatty acids
Level 3	Diet high in sodium
Level 2	Intimate partner violence
Level 2	Childhood sexual abuse and bullying
Level 3	Childhood sexual abuse
Level 3	Bullying victimization
Level 2	Unsafe sex
Level 2	Low physical activity
Level 1	Metabolic risks
Level 2	High fasting plasma glucose
Level 2	High LDL cholesterol
Level 2	High systolic blood pressure
Level 2	High body-mass index
Level 2	Low bone mineral density
Level 2	Impaired kidney function

Within the independent BoD studies, results were summarized and discussed by methodological approach (i.e. bottom-up versus top-down approach) even though the focus of the paper is on CRA. We analysed the elements used to compute the attributable burden, including causality and uncertainty implications. Proving causality refers to the identification of the risk-outcome pairs, going beyond finding a significant association between the risk factor and the risk of developing a certain health outcome.

## Results

### Study selection

**Fig. 1:**
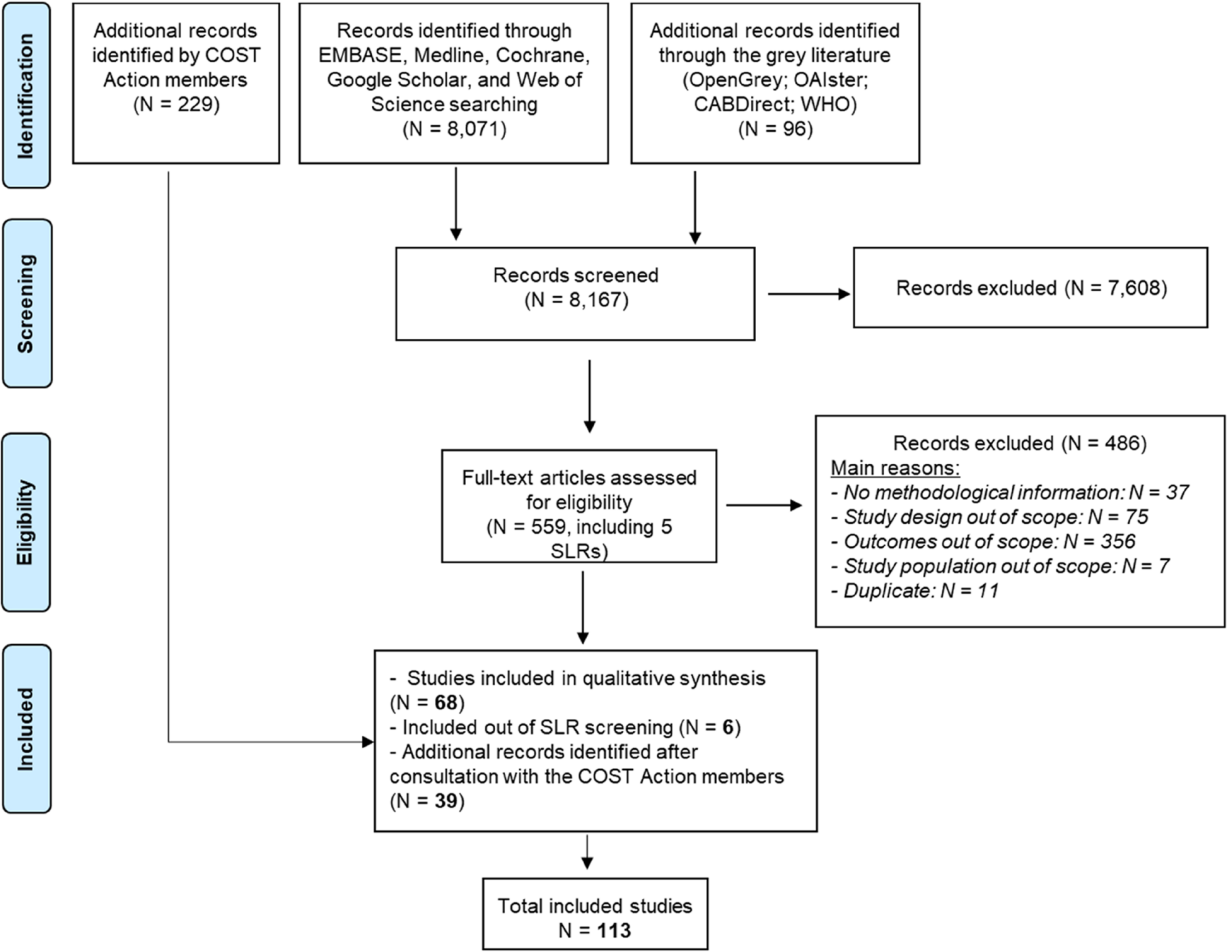
PRISMA flow chart

The database and grey literature searches resulted in 8,167 records after elimination of duplicates. After screening of titles and abstracts, 559 studies including 5 systematic reviews were brought forward to full text screening and assessed for eligibility. The total number of articles that met inclusion criteria was 74. From these, 68 were selected from the review and 6 from the reference list of the systematic reviews retrieved. In addition to the database and grey literature screening, 39 additional studies were eligible for inclusion following the consultation with the burden-eu members (Fig. 1). The latter studies included, but were not limited to, non-English studies and national BoD reports.

Within the 113 selected full-texts, 107 used CRA for the estimation of the attributable burden and 6 used other types of modelling. Therefore, the following subsections will focus on the CRA studies, but we dedicated one paragraph to the synthesis of the methods of the other types of modelling.

### Comparative risk assessment

#### Descriptive analysis

**Fig. 2:**
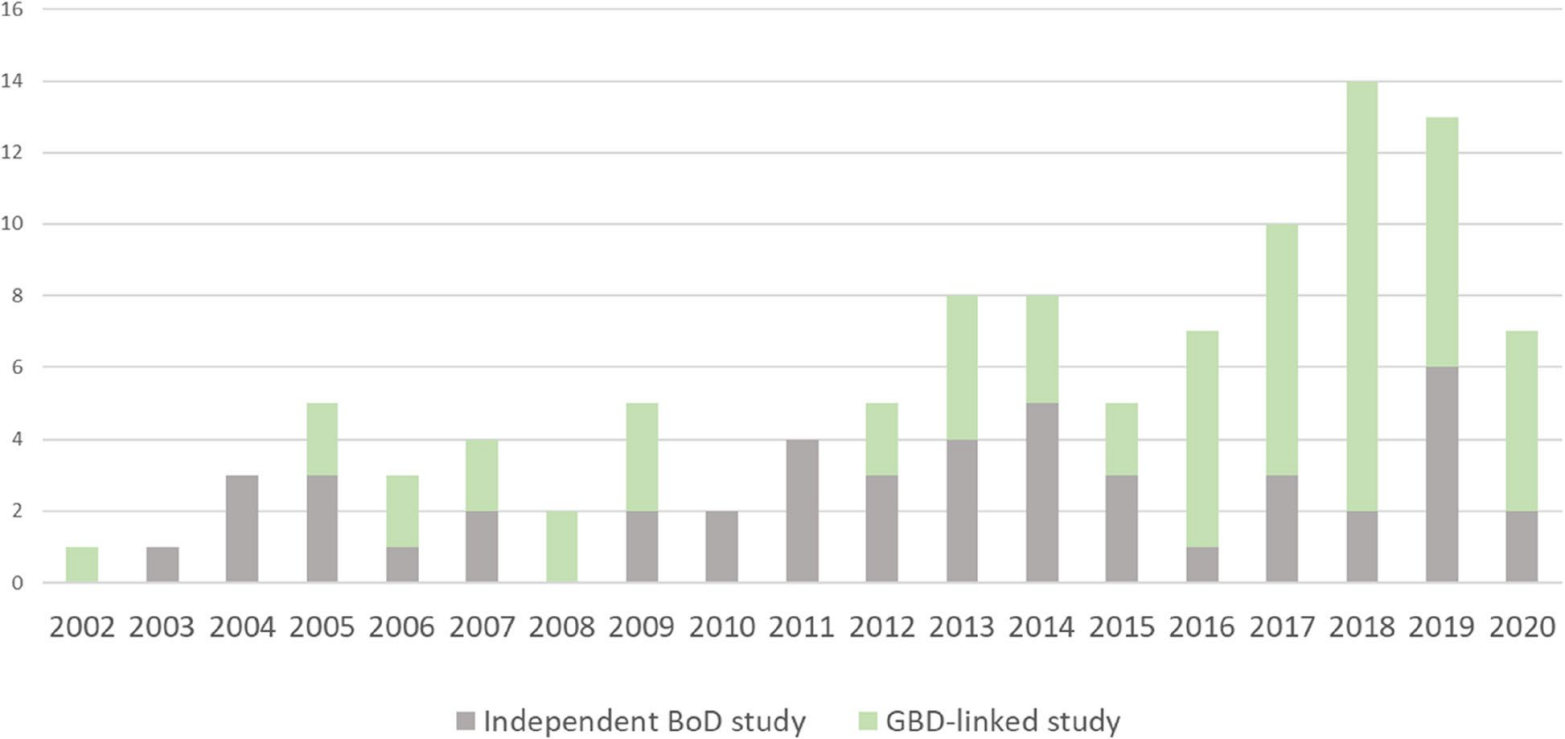
Number of studies by publication year and by type of study

Out of the 107 included studies, 48 papers were independent BoD studies, whereas 59 were GBD-linked studies. Data from multiple countries was included in 58 studies; they were mainly studies at the global level (44 studies) or focusing on the European region (12 studies). In total, 49 studies analysed data from one country only, with the United Kingdom being the most represented country (7 studies), followed by Greece, Portugal, and Sweden (4 studies each). Figure 2 shows number of papers by publication year and the type of study. GBD-linked studies were more concentrated in literature published after 2011, whereas independent BoD studies are more evenly distributed over the years with an increase in the latest years.

Considering level 1 risk factors, most studies investigated behavioural risk factors (97 studies), followed by environmental risk factors (54 studies), metabolic risk factors (46 studies) and occupational risk factors (34 studies). Six studies included risk factors that do not fall under these categorizations. These were type 2 diabetes and major depressive disorders (regarded as a disease by GBD), low socioeconomic position, use of oral contraceptives and use of hormone replacement therapy. The most investigated level 2 risk factors were air pollution, alcohol use and tobacco use (16 studies each).

#### Exposure assessment

**Fig. 3:**
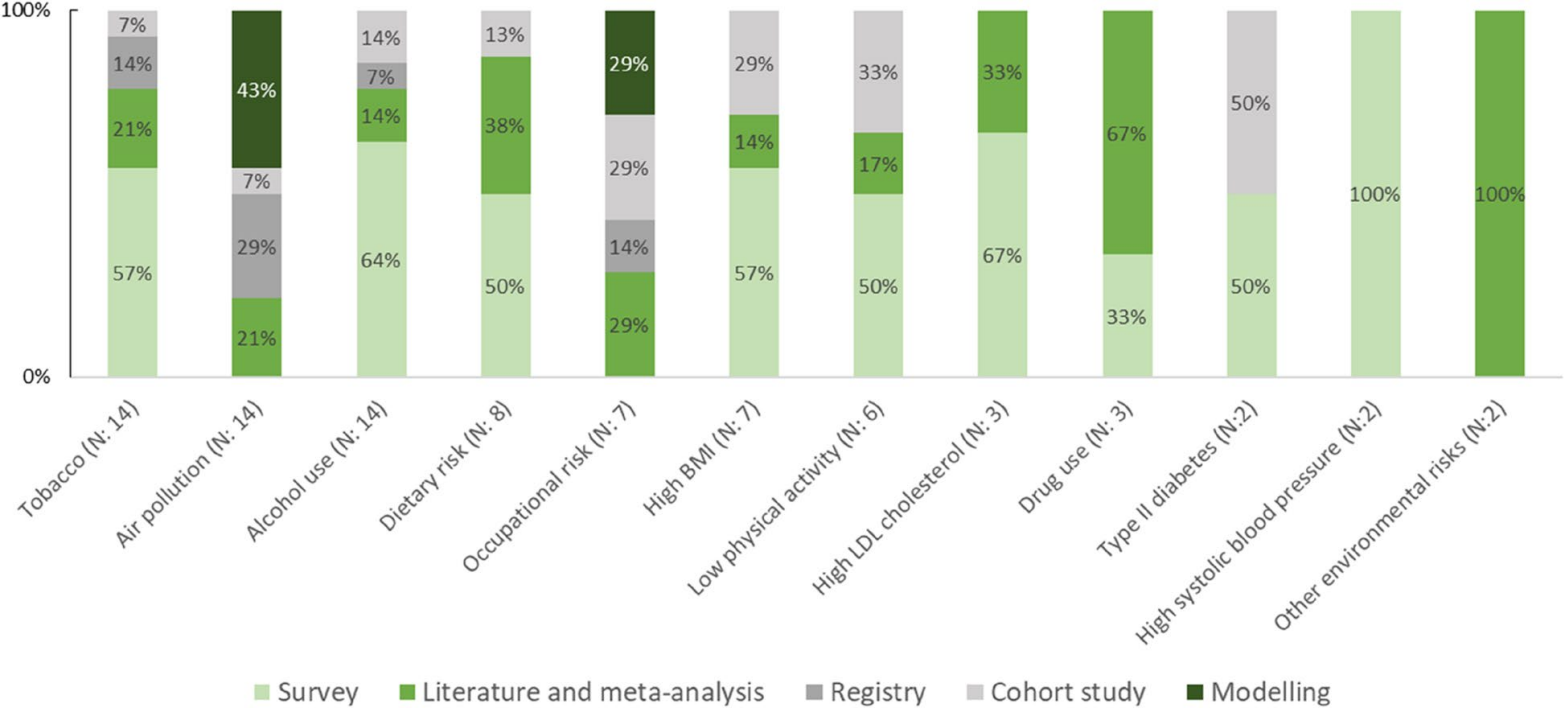
Percentage of types of data sources used for exposure assessment in independent BoD studies by risk factor (level 2)—Only risk factors that were investigated more than once are included in the figure; BMI: Body Mass Index

Figure 3 shows the type of data used for the exposure assessment by level 2 risk factors. All the independent BoD studies clearly defined the risk factor analysed and the associated exposure of the respective population. Only four studies omitted how participants were exposed to the risk factor. The most common data source type for the exposure assessment was survey data (38% of the independent studies), followed by data from the literature (35%) and from registries (23%). Nine independent studies (19%) used modelling techniques to assess the exposure levels. Almost all of these ten studies investigated the BoD attributable to environmental and occupational risk factors, such as air pollution and environmental noise. Tobacco, alcohol use, and occupational risk showed the highest variation in types of data sources.

#### Population attributable fraction and its components

Among the independent BoD studies, the top-down approach including the use of the PAF was the most common methodology to estimate the BoD attributable to risk factors. 87% (46 papers) of all independent studies used PAF, but out of these around 9% (4 studies) did not use the term “PAF”. Examples of different wordings used were: impact fraction [16] and effect factor [17]. Results of the data extraction for these independent studies are reported in Additional file 4.

All the independent studies that computed PAF used RR to express the exposure–response relationship for the risk-outcome-pair, with some exceptions being hazard ratios (HR), in four studies, and odds ratios (OR), in five studies. The main source for RR/OR/HR were single studies (43%), literature reviews or meta-analyses (27%). 14% of the studies that used RR/OR/HR derived these within the context of the study. For example, Papadimitriou et al. [18] and Tsilidis et al. [19] derived HR for smoking using Cox models within a cohort study. Lelieveld et al. [20] developed HR functions for the exposure to air pollution using the Global Exposure Mortality Model. It is worth mentioning the case of Knol et al. [21], investigating the BoD attributable to ultraviolet (UV) radiation: the authors reported that induction and development of skin cancer due to UV exposure is a complicated and long-term process, leading to a complicated exposure–response relationship. Therefore, the authors decided to estimate the burden attributable to UV exposure based on the observed total number of cases with skin cancer in a certain population.

Another crucial element of the CRA approach is the choice of the counterfactual scenario, the TMREL. Nine studies (17% of the independent BoD studies) did not report any information about the counterfactual value. The majority (81%) used a fixed value to express the counterfactual value, whereas two studies used a distribution. In the case of a fixed numeric counterfactual value, 71% of studies defined it as the exposure level corresponding to a RR of 1 for the outcome of interest. Air pollution and dietary risks were the cases where the risk factor level was more commonly set to different scenarios, but with substantial differences among studies. For example, the TMREL of PM_10_ concentration could vary from a reduction of 3,5 μg/m^3^ to setting the level to 20 μg/m^3^.

#### Causality and uncertainty

Thirteen independent BoD studies investigated or acknowledged causality in their CRA. The majority of these (54%) discussed the causality relation, for example describing the complexity of the causal chain [22] or acknowledging the assumptions of causality in the study [23, 24]. In the remaining studies, causality was investigated in the data sources used to compute the attributable burden. In Rehm et al. [25], authors used the epidemiological criteria of causality presented in Rothman & Greenland 1998 [26], where the association between risk factor and disease was included only if a biological pathway was identified [25]. In Tod et al. [27], Directed Acyclic Graphs (DAG) were used to illustrate causal relationships between exposure to different risk factors and stroke, identifying the total effect for an exposure using mediation analysis.

Half of the independent BoD studies (50%) performed uncertainty analysis. All these studies reported parametric uncertainty: 21% used bootstrap and 17% Monte Carlo simulations. The rest of the studies did not provide details about their uncertainty analysis. Eight studies conducted a scenario analysis. Among the elements changed, the definition and the source of the exposure levels, using different disability weights and applying age-weighting and time discounting are included.

When looking at which uncertainty elements were included in the analysis, we see that analysis on the uncertainty associated with the exposure levels was the least reported (63% of the studies that performed uncertainty analysis) compared to uncertainty on the RR and on the morbidity or mortality outcomes (96%).

### Studies using other approaches than CRA

The studies that did not apply CRA (6 studies) used other common methods of analysis, such as logistic regression and stratification analysis, Markov models or a risk assessment approach, where the estimated burden is derived from the integration of a dose–response relationship with exposure to the risk factor. The latter approach was used for dietary risk factors in De Oliveira Mota et al. [28], where they estimated the excess risk of having colorectal cancer when consuming red meat among the French population assuming that people either ate or did not eat red meat in France. Similarly, in Jakobsen et al. [29] computed the burden attributable to acrylamide exposure using a bottom-up approach. In such a study, the slope factor expressed the increase in the risk of cancer per daily unit of exposure to the carcinogen derived by the dose–response function, to estimate the number of annual cancer cases caused by exposure to acrylamide. The burden was estimated without scaling into an existing disease envelope. The impact of air pollution was also investigated with a bottom-up approach in Orru et al. [30] using RR for respiratory and cardiovascular hospitalizations and the estimated excess exposure to PM_10_. Dzhambov et al. 2015 estimated the probability of highly annoyed people by road traffic noise using logistic response functions [31].

Stratification analysis was conducted in May et al. [32] combining the estimation of the probability of higher DALY looking at individual lifestyle factors using logistic regression, and Meijerink et al. [33] used Markov models to estimated DALY attributable to drug intake.

## Discussion

The aim of our scoping literature review was to compile studies that assessed the BoD attributable to risk factors performed in Europe since 1990, with a main focus on CRA. We extracted data on the data input sources and methodological choices needed to compute YLL, YLD or DALY attributable to one or more risk factors. A total of 113 papers were identified. Within them, 107 used the CRA approach and were categorized as either independent BoD or GBD-linked studies. Our results showed that the methods used to perform CRA varied substantially across independent European BoD studies. While there were some methodological choices that are more common than others, we did not observe patterns in terms of country, year or risk factor. All the different methodological choices could affect the comparability of estimates between and within countries and/or risk factors, since they might significantly influence the quantification of the attributable burden.

In general, our review showed a propensity in Europe to explore behavioural risk factors more than others. Tobacco use, air pollution and alcohol use are the most analysed risk factors. This might be due to the fact that causal relationships for these risk factors are more studied, together with availability of better exposure data (e.g. tobacco and cancer). These risk factors are obviously important, but there are other risk factors that while equally important, have not been widely studied in a European population. Particular attention could be drawn to the relationship between dietary risks/low physical activity and cardiovascular disease, highly prevalent in European countries. Independent studies analysed risk factors not included in GBD, like low socioeconomic status and depressive disorders. The definition of risk factor might also be a point of discussion when some diseases can also be regarded as risk factors. In this study, we defined a risk factor as every individual behavioural choice or environmental factor that affects the risk associated to a disease outcome. We used the categorization adopted in the GBD studies but also included risk factors that were not regarded as such in the GBD framework. An example is type 2 diabetes which could increase the risk of cancer. This can be problematic since it is difficult to establish if the risk factor triggered a chronic diseases or if it was triggered by a chronic disease. For example, depressive disorders can develop in the setting of chronic disease such as dementia but can also result in the development of chronic disease [34]. That is why the investigation of causality is a keystone for the estimation of the attributable burden.

From 2000 onwards, the most common methodology was the use of PAF with RR and a counterfactual value set to the category with the lowest risk, as performed in the GBD study [14]. Deviations from the GBD framework included even differences in basic concepts, like the definition of risk factor or the terminology used to refer to PAF. Even though the diversity in terminology does not affect the final results, it hampers comparison of results across studies and adds to confusion in the interpretation of the methods and results.

Among independent BoD studies, many differences were observed for the exposure–response function, in its definition and source. The latter particularly varies across studies, with less than half of the studies using meta-analysis and literature reviews. Different sources and definitions can lead to different estimations of the attributable burden within the same risk factor. Another important choice in the CRA framework is the selection of the TMREL. The great majority of the studies decided to set it to the category at the lowest risk, which is implicit in the CRA methodology. However, this was rarely specified in the papers, affecting the comparability across countries, diseases and/or risk factors. Exploring different optimal scenarios was not uncommon in our review and was used to assess the impact of different interventions, most commonly in air pollution, alcohol and smoking. This is often referred to as health impact assessment (HIA), where the CRA methodology and HIA go hand in hand but for which the difference in purposes is often neglected.

The remaining six studies used an approach other than CRA, which was more common for some types of risk factors and outcomes. These included environmental and occupational risk factors, which more commonly employ bottom-up approaches and health outcomes where disease envelopes may not be available. This could be explained by the fact that traditionally this category of risk factors may have been included in a toxicological risk assessment of chemicals-approach, where for example exposure–response functions were derived with another purpose than a quantitative estimate of number of incident cases.

Our review highlighted some gaps in the uncertainty analyses and the investigation of causal relationships of BoD studies. Half of the independent BoD studies did not perform uncertainty analysis, and half of those that took into account parameter uncertainty did not report important methodological information like the method used for the analysis. Uncertainty on exposure–response functions was more frequently propagated than other inputs, such as exposure assessments. Less than half of the studies investigated or discussed causality in their CRA. Although the gold standard for concluding on causality is often considered to be a randomized controlled trial, in practice researchers often must rely on the strength of evidence that is brought by a variety of studies. It is therefore important to discuss causality in a risk factor assessment exercise [35]. On the other hand, causality might be very difficult to prove for certain hazards and restricting inclusion of health effects to only those where causality is proven might underestimate the true burden. For many diseases causality is multifactorial leading to a difficulty to clearly disentangle the burden of each risk factor on a determinate disease, as well as how different risk factors may further interact with each other. In addition, randomized controlled trials are often not feasible due to numerous reasons, e.g. resources availability, ethical controversies. Scenario analysis was undertaken in very few independent studies but represents an essential tool for exploring the impact of different methodological choices and inclusion or exclusion of health outcomes of varying degree of causality.

The detected lack of consistency in terminology and methods makes comparisons and interpretation of results more challenging. Well-established guidelines that can be used in future studies estimating risk factor attributable burden could be achieved by publication of handbooks, manuals, protocols, etc. While heterogeneity is inevitable, it is important to make assumptions and methodological choices explicit, and to discuss possible limitations or develop alternative scenarios to quantify the associated uncertainties.

### Strengths and limitations

Our scoping literature review brings together existing risk factor BoD studies undertaken in Europe. We comprehensively reviewed the methodological choices and assumptions used to calculate the BoD attributable to risk factors in terms of YLL, YLD, and DALY within CRA studies. This literature review used a variety of literature databases and search engines, as well as the consultation with European experts that work in the field of BoD in their respective countries. Nevertheless, our search may be limited by the nature of the grey literature searched and the national public health websites targeted, where some BoD studies may have been missed. In contrast to what is commonly done in systematic literature reviews, we did not perform a quality assessment of the included studies. Considering that no estimates were extracted but only methodological information, we did not consider a bias assessment relevant for the objectives of this literature review. In addition, to the best of our knowledge, there is not a tool that was specifically develop for evaluating the quality of BoD studies and CRA studies. For this reason, the results of this review will be used to feed future developments of these kind of bias assessment tools. Within our study we focused on the methodological choices of CRA studies. Considering the importance of other methods, we decided to also include the non-CRA studies identified by our search string. Nevertheless, since the search strategy focused on CRA, some risk assessment studies might have been disregarded. This limitation might be mitigate by the access to a wide international network that helped us finding and translating independent and/or GBD-linked BoD studies.

### Research implications

This review is part of a series of reviews [5, 9] that aims to compile BoD studies in Europe and to summarize methodological choices in the estimation of DALY. Each review focuses on the assessment of methodological design choices that were used in studies assessing the burden of non-communicable, injuries, infectious diseases, and risk factors. One of the main aims of the *burden-eu* network is to provide a standardized statement for reporting DALY calculations in BoD studies. The development and use of key standardized guidelines for reporting BoD methodological choices may help to have more accessible BoD estimates. Our literature review serves as a critical input for such developments since we underlined the necessity for transparency and uniformity in risk factor BoD studies.

## Conclusions

In this scoping literature review we examined independent studies that assessed the burden attributable to risk factors in the GBD European Region countries. When looking at the methodological choices applied in these studies, we observed considerable variation across countries and risk factors.

We identified a series of methodological design choices that hamper the comparability of results. Above all, the profound differences in the two most used methods, CRA and risk assessment. We also noticed a lack of transparency when reporting methods and a limited consideration of uncertainty and causality.

There is a strong need for the development and use of guidelines for performing and reporting BoD studies to help understand differences and avoid misinterpretations.

## Supplementary Information

**Additional file 1.** Search Strategy.

**Additional file 2.** Grey literature search and websites of targeted national public health agencies.

**Additional file 3.** Definitions of the data extraction items.

**Additional file 4.** Studycharacteristics, exposure assessment data sources and methodological choices ofthe 46 independent studies using a top-down approach.

**Additional file 5.** Reference list of the included disease burden studies.

**Additional file 6**. Reference list of the excluded studies, including reason for exclusion.

## Data Availability

All data generated or analysed during this study are included in this published article and its supplementary information files.
