# Supplementary material for: Burden of disease attributable to risk factors in European countries: a scoping literature review
Source: Arch Public Health. 2023 Jun 25;81:116. doi: 10.1186/s13690-023-01119-x (PMC10290804; doi:10.1186/s13690-023-01119-x)
Supplement: Supplementary file 1 — Additional file 1. Search Strategy. [file 13690_2023_1119_MOESM1_ESM.docx]

Additional file 1: Search Strategy

**Embase**

('attributable risk'/de OR (((attribut*) NEAR/3 (risk* OR fraction* OR burden* OR mortalit* OR death*)) OR ((comparat*) NEAR/3 (risk*) NEAR/3 (assessment*))):ab,ti,kw) AND ('Europe'/exp OR 'Yugoslavia'/de OR 'Israel'/de OR 'European Union'/de OR 'European'/de OR 'EU citizen'/de OR (europ* OR austria* OR belgium OR belgian* OR Denmark OR danish OR france OR french* OR german* OR ireland OR irish* OR italy OR italian* OR luxemb* OR netherlands OR dutch OR norway OR sweden OR switzerland OR swiss OR united-kingdom OR albania OR armenia OR bosnia* OR herzegovin* OR bulgar* OR croatia* OR cyprus OR czechoslovakia* OR estonia* OR finland OR georgia OR greece OR hungar* OR iceland* OR israel* OR kosov* OR latvia* OR lithuan* OR macedoni* OR malta OR montenegr* OR poland OR polish OR portug* OR romani* OR rumani* OR serbi* OR slovak* OR sloven* OR spain* OR spanish OR turkey* OR mediterran* OR czech* OR england* OR UK OR scotland OR wales OR britain* OR holland* OR scandinav* OR nordic-countr* OR yugoslov* OR baltic* OR flander* OR wallon* OR benelux* OR greek* OR andorra* OR azerbaijan* OR belarus* OR byelarus* OR byelorus* OR russia* OR monaco* OR moldova* OR moldovia* OR san-marin* OR ukrain*):ab,ti,kw) AND [1990-2030]/py

**Medline**

((((attribut*) ADJ3 (risk* OR fraction* OR burden* OR mortalit* OR death*)) OR ((comparat*) ADJ3 (risk*) ADJ3 (assessment*))).ab,ti,kf.) AND (exp Europe/ OR Yugoslavia/ OR Israel/ OR European Union/ OR (europ* OR austria* OR belgium OR belgian* OR Denmark OR danish OR france OR french* OR german* OR ireland OR irish* OR italy OR italian* OR luxemb* OR netherlands OR dutch OR norway OR sweden OR switzerland OR swiss OR united-kingdom OR albania OR armenia OR bosnia* OR herzegovin* OR bulgar* OR croatia* OR cyprus OR czechoslovakia* OR estonia* OR finland OR georgia OR greece OR hungar* OR iceland* OR israel* OR kosov* OR latvia* OR lithuan* OR macedoni* OR malta OR montenegr* OR poland OR polish OR portug* OR romani* OR rumani* OR serbi* OR slovak* OR sloven* OR spain* OR spanish OR turkey* OR mediterran* OR czech* OR england* OR UK OR scotland OR wales OR britain* OR holland* OR scandinav* OR nordic-countr* OR yugoslov* OR baltic* OR flander* OR wallon* OR benelux* OR greek* OR andorra* OR azerbaijan* OR belarus* OR byelarus* OR byelorus* OR russia* OR monaco* OR moldova* OR moldovia* OR san-marin* OR ukrain*).ab,ti,kf.) AND 1990:2030.(sa_year).

**Cochrane**

((((attribut*) NEAR/3 (risk* OR fraction* OR burden* OR mortalit* OR death*)) OR ((comparat*) NEAR/3 (risk*) NEAR/3 (assessment*))):ab,ti) AND ((europ* OR austria* OR belgium OR belgian* OR Denmark OR danish OR france OR french* OR german* OR ireland OR irish* OR italy OR italian* OR luxemb* OR netherlands OR dutch OR norway OR sweden OR switzerland OR swiss OR united-kingdom OR albania OR armenia OR bosnia* OR herzegovin* OR bulgar* OR croatia* OR cyprus OR czechoslovakia* OR estonia* OR finland OR georgia OR greece OR hungar* OR iceland* OR israel* OR kosov* OR latvia* OR lithuan* OR macedoni* OR malta OR montenegr* OR poland OR polish OR portug* OR romani* OR rumani* OR serbi* OR slovak* OR sloven* OR spain* OR spanish OR turkey* OR mediterran* OR czech* OR england* OR UK OR scotland OR wales OR britain* OR holland* OR scandinav* OR nordic-countr* OR yugoslov* OR baltic* OR flander* OR wallon* OR benelux* OR greek* OR andorra* OR azerbaijan* OR belarus* OR byelarus* OR byelorus* OR russia* OR monaco* OR moldova* OR moldovia* OR san-marin* OR ukrain*):ab,ti)

**Web of Science**

TS=(((((attribut*) NEAR/2 (risk* OR fraction* OR burden* OR mortalit* OR death*)) OR ((comparat*) NEAR/2 (risk*) NEAR/2 (assessment*)))) AND ((europ* OR austria* OR belgium OR belgian* OR Denmark OR danish OR france OR french* OR german* OR ireland OR irish* OR italy OR italian* OR luxemb* OR netherlands OR dutch OR norway OR sweden OR switzerland OR swiss OR united-kingdom OR albania OR armenia OR bosnia* OR herzegovin* OR bulgar* OR croatia* OR cyprus OR czechoslovakia* OR estonia* OR finland OR georgia OR greece OR hungar* OR iceland* OR israel* OR kosov* OR latvia* OR lithuan* OR macedoni* OR malta OR montenegr* OR poland OR polish OR portug* OR romani* OR rumani* OR serbi* OR slovak* OR sloven* OR spain* OR spanish OR turkey* OR mediterran* OR czech* OR england* OR UK OR scotland OR wales OR britain* OR holland* OR scandinav* OR nordic-countr* OR yugoslov* OR baltic* OR flander* OR wallon* OR benelux* OR greek* OR andorra* OR azerbaijan* OR belarus* OR byelarus* OR byelorus* OR russia* OR monaco* OR moldova* OR moldovia* OR san-marin* OR ukrain*)))

**Google Scholar (200 top ranked)**

"attributable risk|fraction|burden|mortality"|"comparative risk assessment" europe|france|germany|italy|netherlands|norway|sweden|switzerland|"united kingdom"|finland|greece|hungaria|israel|poland|portugal|romania|spain|turkey|england|britain|scandinavia
