# Supplementary material for: Burden of disease attributable to risk factors in European countries: a scoping literature review
Source: Arch Public Health. 2023 Jun 25;81:116. doi: 10.1186/s13690-023-01119-x (PMC10290804; doi:10.1186/s13690-023-01119-x)
Supplement: Supplementary file 2 — Additional file 2. Grey literature search and websites of targeted national public health agencies. [file 13690_2023_1119_MOESM2_ESM.docx]

Additional file 2: Grey literature search and websites of targeted national public health agencies

*Grey literature search engines*

| - OpenGrey: [www.opengrey.eu](http://www.opengrey.eu) | - **CABDirect:** [www.cabdirect.org](http://www.cabdirect.org) |
| --- | --- |
| - OAIster: http://oaister.worldcat.org | - **World Health Organization:** [www.who.int](http://www.who.int) |

*Websites of targeted national public health agencies*

| - Albania: [www.ishp.gov.al](http://www.ishp.gov.al) | - **Malta:** [www.deputyprimeminister.gov.mt/](http://www.deputyprimeminister.gov.mt/) |
| --- | --- |
| - Andorra: [www.salut.ad](http://www.salut.ad) | - **Montenegro:** [www.ijzcg.me/](http://www.ijzcg.me/) |
| - Austria: [goeg.at/](https://goeg.at/) | - **Netherlands:** [www.rivm.nl](http://www.rivm.nl/) |
| - Belarus: [minzdrav.gov.by/en/](http://minzdrav.gov.by/en/) | - **North Macedonia:** [www.iph.mk](http://www.iph.mk/) |
| - Belgium: [www.sciensano.be/en](http://www.sciensano.be/en) | - **Norway:** [www.fhi.no](http://www.fhi.no/) |
| - Bulgaria: [ncpha.government.bg](http://ncpha.government.bg) | - **Poland:** [www.pzh.gov.pl](http://www.pzh.gov.pl/) |
| - Cyprus: [www.moh.gov.cy/](http://www.moh.gov.cy/) | - **Portugal:** [www.dgs.pt](http://www.dgs.pt); [www.sns.gov.pt](http://www.sns.gov.pt) |
| - Czech Republic: [www.szu.cz](http://www.szu.cz) | - **Republic of Moldova:** [www.msmps.gov.md/](http://www.msmps.gov.md/) |
| - Denmark: [www.si-folkesundhed.dk](http://www.si-folkesundhed.dk) | - **Romania:** [www.insp.gov.ro/](https://www.insp.gov.ro/) |
| - Estonia: [www.tai.ee](http://www.tai.ee) | - **Russian Federation:** [www.minzdrav.gov.ru](http://www.minzdrav.gov.ru) |
| - Finland: [www.thl.fi/en/](http://www.thl.fi/en/) | - **Serbia:** [www.batut.org.rs/english.html](http://www.batut.org.rs/english.html) |
| - France: [www.santepubliquefrance.fr](http://www.santepubliquefrance.fr) | - **Slovakia:** [www.uvzsr.sk/en/](http://www.uvzsr.sk/en/) |
| - Germany: [www.rki.de](http://www.rki.de/EN) | - **Slovenia:** [www.nijz.si](http://www.nijz.si/) |
| - Greece: [www.statistics.gr](http://www.statistics.gr); [www.eody.gov.gr](http://www.eody.gov.gr) | - **Spain:** [www.isciii.es](http://www.isciii.es) |
| - Hungary: [www.nnk.gov.hu/](http://www.nnk.gov.hu/) | - **Sweden:** [www.folkhalsomyndigheten.se](http://www.folkhalsomyndigheten.se/) |
| - Iceland: [www.landlaeknir.is](http://www.landlaeknir.is/) | - **Switzerland:** [www.bag.admin.ch/bag/de/home.html](http://www.bag.admin.ch/bag/de/home.html) |
| - Ireland: [www.publichealth.ie](http://www.publichealth.ie/) | - **Turkey:** [www.saglik.gov.tr/?_Dil=2](http://www.saglik.gov.tr/?_Dil=2) |
| - Israel: [www.gov.il/](http://www.gov.il/) | - **Ukraine:** [en.moz.gov.ua/](https://en.moz.gov.ua/) |
| - Italy: [www.iss.it/](http://www.iss.it/)  - **Latvia:** [www.rsu.lv/en/institute-public-health](https://www.rsu.lv/en/institute-public-health)  - **Lithuania:** [sam.lrv.lt/en/](https://sam.lrv.lt/en/)  - **Luxembourg:** [www.lih.lu/](https://www.lih.lu/) | - **United Kingdom (Scotland):** [www.gov.uk/government/organisations/public-health-england](https://www.gov.uk/government/organisations/public-health-england); [www.scotpho.org.uk/](http://www.scotpho.org.uk/) |
