# Supplementary material for: Burden of disease attributable to risk factors in European countries: a scoping literature review
Source: Arch Public Health. 2023 Jun 25;81:116. doi: 10.1186/s13690-023-01119-x (PMC10290804; doi:10.1186/s13690-023-01119-x)
Supplement: Supplementary file 3 — Additional file 3. Definitions of the data extraction items. [file 13690_2023_1119_MOESM3_ESM.docx]

Additional file 3: Definitions of the data extraction items

| General Information | PMID | A unique identifier number which assigned to a specific reference/article in the PubMed website. |
| --- | --- | --- |
|  | Journal | The name of the journal that published the selected article. |
|  | Title | The full title of the selected article. |
|  | Author(s) | List of author(s) with the use of **Vancouver style**. |
|  | Year | The year that the selected burden of disease (BoD) study was published. |
|  | Funding body  *(optional)* | A type of funding source, i.e.: government, institutional administrators, private industry, foundations, professional organisation, etc. |
|  | Language | The written language of the BoD study |
| Study characteristics | Cause of ill health | A single disease related to communicable or non-communicable disease or injury or an aggregation of diseases and injuries.  i.e., Non-communicable diseases (NCDs) [Yes/No]; Communicable diseases (CDs) [Yes/No]; Injuries [Yes/No] |
|  | Risk factor | A risk factor or a group of risk factors analysed in the paper |
|  | Type of study | **Independent study** (i.e., single-country or multi-country studies that performed own calculations and analyses of years of life lost (YLL), years –lived with disability (YLD) and/or disability-adjusted life years (DALY)) *versus* **GBD-linked study** (i.e., single-country or multi-country studies in which YLLs, YLDs and/or DALYs were derived from the existing Global Burden of Disease (GBD) study estimates. |
|  | Reference population | Population whose health causes during some period of time is the source of the study data. |
|  | Reference year | The year for which an estimate of incidence/prevalence/BoD is reported. |
|  | Stratification | The specific cause(s), and/or disease(s), and/or risk factor(s) related to mortality or disability indicators stratified for each year, age, and sex. [Yes/No] |
| Data input sources  Data input sources  *(continued)* | Data source mortality/YLL | *Were data sources that were used to derive mortality/YLL data specified by the authors?* [Yes/No]  *Relevant data sources for mortality data:* National statistics, disease registries, registry of death, survey data, vital registration systems, verbal autopsies, death registration systems, published literature, etc. |
|  | Mortality/YLL:  data integration | *Were multiple data sources integrated to arrive at the mortality/YLL data?* [Yes/No] |
|  | Data source incidence/prevalence/YLD  Data source incidence/prevalence/YLD  *(continued)* | *Were data sources that were used to derive incidence/prevalence/YLD data specified by the authors?* [Yes/No]  *Relevant data sources for morbidity data:* Police records, health service encounter data, emergency department visits, hospital admissions, etc.  *Other relevant data sources for morbidity data:* Published literature, disease registries, routine administrative and survey datasets, surveillance systems, health facility data, etc |
|  | Incidence/prevalence/YLD: data integration | *Were multiple data sources integrated to arrive at the incidence/prevalence/YLD data?* [Yes/No] |
| Data adjustments | Mortality/YLL:  data adjustment | [Yes/No] |
|  | Incidence/prevalence/YLD: data adjustment | [Yes/No] |
|  | Internal consistency | *Were adjustments made to ensure that the sum of cause‐specific mortality or impairments equals all‐cause mortality or impairments?* |
|  | Use of DisMod | DisMod is s a software tool that may be used to check the consistency of estimates of incidence, prevalence, duration and case fatality for diseases.  *Did the authors mention the use of DisMoD?* [Yes/No] |
| CRA specific methods | Definition of the risk factor | 1. Was the risk factor clearly defined? (e.g. for air pollution: NO2, PM..) [Yes/No] |
|  |  | 1. Was the exposure to the risk factor clearly defined? (e.g. exposed by active smoking, by breathing dirty air) [Yes/No] |
|  |  | 1. Was the risk factor expressed as a categorical, dichotomous or continuous exposure? |
|  | Data sources of the exposure | 1. What was the data source for the risk factor exposure assessment? 2. *Relevant data sources for exposure assessment:* surveys, literature, registries, etc. |
|  | Risk-Outcome combinations | 1. List of the diseases that were considered in the paper as associated with the risk factor |
|  | Exposure-response function definition | 1. The exposure-response function is the relationship between the exposure to the risk factor and the disease. The relationship can be summarized with different metrics, e.g. relative risks, odds ratios, hazard ratios, etc. |
|  |  | 1. The relationship between exposure and disease can have different shapes, e.g. linear, exponential, non-linear, etc. |
|  | Data sources of the exposure-response function | 1. What was the data source for the exposure-response function? 2. *Relevant data sources for exposure-response function:* single study, multiple studies, meta-analysis or literature reviews, etc. 3. The authors might compute the exposure-response function within the study. |
|  | Causality | 1. Did the study explore the causal relationship between risk factor and outcome? If yes, was it included as part of the methods or only generally discussed? |
|  | Definition of theoretical minimum risk exposure level | 1. In the comparative risk assessment framework, the current level of exposure to the risk factor is compared to a counterfactual level. When the latter is set to 1, the absolute absence of the risk factor is assumed. Alternatively, the author can decide to set it to an alternative level (e.g. 10% reduction of air pollution, ideal calorie intake). It is also common that the counterfactual exposures correspond to the effect of different health interventions that targeted the risk factor of interest. |
|  | Computation of attributable burden | 1. In order to compute the burden of disease attributable to a risk factor, the total number of DALY/YLD/YLL will be multiplied by the population attributable fraction. The latter quantifies the proportion of the disease onset that can be attributed to a risk factor. 2. Alternatively, authors can opt for ad-hoc methods for the quantification of the attributable burden. |
|  | Stratification | The comparative risk assessment was stratified for age, and/or sex. [Yes/No] |
| DALY method  DALY method  *(continued)* | Perspective of YLD estimates | - *Prevalence-based* perspective takes point prevalence measures of disability, adjusted for seasonal variation. - *Incidence-based* perspective captures the BoD in new diagnostic cases during a reference time-period and links all possible sequelae in future through an outcome tree or disease progression model. |
|  | Life expectancy for YLL | The life table that was used to assess YLL  *Relevant life-tables*: Aspirational standard life tables, i.e., WHO standard life table, GBD standard life table OR National life tables or national life expectancy |
|  | Disease model | A disease model is a causal chain of a disease that describes health states and their transition probabilities over time. *Did the authors report the disease model that they have used to assess BoD?* [Yes/No] |
|  | DW: source | The source of the set(s) of disability weights (DWs) that were used to assess YLD.  *Relevant sources*: GBD DWs, Dutch DWs, Empirical DWs etc. |
|  | DW: elicitation method  (only if study developed own DWs) | Methods for eliciting health state valuations  *Relevant methods*: Visual Analogue Scale (VAS), Person Trade-Off (PTO), Time Trade-Off (TTO), etc. |
|  | DW: panel of judges  (only if study developed own DWs) | The panel of judges whose preferences were obtained to assess DWs  *Relevant panel composition*: medical experts, healthcare professionals, policymakers, patients or people with disabilities, patients’ families, etc. |
|  | DW: severity distribution | The proportion of cases with e.g., mild, moderate or severe health state^1^ of a specific outcome for which separate DWs are available.  *Was a severity distribution used/reported by the authors?* [Yes/No]; [Global/National]  ^1^: a health state reflects a combination of signs or symptoms that result in a certain amount of health loss |
|  | Comorbidity adjustment (YLD calculation) | Adjustment of YLD data for comorbidity^2^ [Yes/No]  ^2^: multiple conditions co-existing in one individual |
|  | Methods co-morbidity adjustment (YLD calculation) | - Approach(es) used to deal with the impact of comorbidity in a BoD study e.g., Standard simulation method, etc  - Approach(es) that can be used to adjust DW’ data for comorbidity  e.g., Additive approach, Multiplicative approach, Maximum limit approach etc |
|  | Social weighting:  age weighting | By incorporating age-weighting into DALY implies that the value of life depends on age; a lower weight of healthy life years lived is given at younger and at older ages – known as ‘non-uniform DALY’ [Yes/No] |
|  | Social weighting:  time discounting | Time-discounting discounts future years of healthy life lived using a rate of 3% or an alternative set of 0% [Yes/No] |
|  | Social weighting: discounting rate | ‘*uniform DALY’*; age-weighting and 3% time-discounting rate; ‘*non-uniform DALY’*; no age-weighting, no time-discounting rate; ‘*age-weighting DALY’*; age-weighting, no time-discounting rate; ‘*time-discounting DALY’*; no age-weighting, 3% time-discounting rate [%] |
| Uncertainty | Uncertainty analysis | An estimation of range or distribution of uncertainty in estimates based on an assessment of the uncertainty or confidence intervals for all data and parameter inputs [Yes/No] |
|  | Uncertainty analysis: method | *Relevant methods of uncertainty in DALY calculations:* Parameter uncertainty, Structural or model uncertainty, Methodological uncertainty |
|  | Sensitivity analysis | Analysis of how the impact of uncertainties of one or more input variables can lead to uncertainties in data inputs or assumptions [Yes/No] |
|  | Scenario analysis | The current or future disease burden is compared with the BoD if one element is changed (e.g., life expectancy, DW or severity distribution) [Yes/No] |
|  | Scenario analysis: element changed | Element that was changed for the scenario analysis (e.g., life expectancy, DW or severity distribution) |
| BoD: burden of disease; DALY: disability adjusted life years; DW: disability weight; GBD: Global Burden of Disease; PMID: PubMed Identifier; YLD: years lost due to disability; YLL: years of life lost due to premature mortality; WHO: World Health Organisation | | |
