# Supplementary material for: Burden of disease attributable to risk factors in European countries: a scoping literature review
Source: Arch Public Health. 2023 Jun 25;81:116. doi: 10.1186/s13690-023-01119-x (PMC10290804; doi:10.1186/s13690-023-01119-x)
Supplement: Supplementary file 4 — Additional file 4. Studycharacteristics, exposure assessment data sources and methodological choices ofthe 46 independent studies using a top-down approach. [file 13690_2023_1119_MOESM4_ESM.docx]

**Additional file 4: Study characteristics, exposure assessment data sources and methodological choices of the 46 independent studies using a top-down approach**

| **Author(s)** | **Year** | **Risk factor** | **Country(ies)/**  **region included** | **Exposure assessment source** | **Effect estimate** | **When ERF is used how was it defined?** | **Source of the ERF** | **Counterfactual value** | **Computation of attributable burden** |
| --- | --- | --- | --- | --- | --- | --- | --- | --- | --- |
| Adam et al | 2012 | Active smoking | Hungary | Literature, registry, National Statistics | RR | Linear | Meta-analysis and large scale cohort studies | 7 % reduction | PAF |
|  |  | Environmental tobacco smoke | Sweden | Registry | RR | Linear | Meta-analysis | 95 % decrease in the prevalence of ETS exposure in the hospitality sector, 70 % in the workplace and 5.9 % in households |  |
| Agardh et al | 2011 | Low socioeconomic position | Sweden | Registry | RR | Linear | meta-analysis | Category with the lowest risk | PAF |
| Begou et al | 2020 | Road traffic noise | Greece | Census data | RR | Log-linear | Meta-analysis | Category with the lowest risk | PAF |
| Bello et al | 2003 | Alcohol | Spain | Registry | RR | NR | Single study | NR | PAF |
| Borges et al | 2009 | Smoking | Portugal | Survey | RR | Linear | Single study | Category with the lowest risk | PAF |
| Cortez-Pinto et al | 2010 | Alcohol | Portugal | Survey | RR | Linear | Single study | Category with the lowest risk | PAF |
| Driscoll et al | 2005 | Occupational carcinogen | Global | Registry | RR | Linear | Literature review | Category with the lowest risk | PAF |
| Effertz et al | 2013 | Substance Use Disorder | Germany | Survey | RR | Linear | Multiple studies | NA | PAF |
| Gouveia et al | 2004 | Hypercholesterolemia | Portugal | National statistics, WHO | RR | Exponential | Single study | Category with the lowest risk | PAF |
| Guillois-Becel et al | 2007 | Air pollution | France, Nantes city | Registry | RR | Exponential | Single study | Reduction 3,5 ug/m3 | PAF (other) |
| Holnicki et al | 2017 | Air pollution | Poland | Metereological stations + model | RR and UR (unit risk) | non-linear | Multiple studies | Category with the lowest risk | PAF |
| Jarosinska et al | 2014 | Second-hand smoking | Poland | Survey | OR | Linear | Multiple studies | Category with the lowest risk | PAF |
| Kassomenos et al | 2013 | Air pollution | Greece | Registry and literature | RR | Linear | Meta-analyses | Category with the lowest risk | PAF (effect factor) |
| Keall et al | 2011 | Modifiable housing conditions | Global | Literature | RR | Linear | Literature review | NR | PAF |
| Knol et al | 2005 | Air pollution | The Netherlands | Modelling | RR | Non-linear | Multiple studies | NR | PAF |
|  |  | Noise |  | Modelling | RR | Non-linear | Multiple studies |  |  |
|  |  | Radiation |  | Literature | RR | Linear | Multiple studies |  |  |
|  |  | UV |  | Literature | NA | NA | NA |  |  |
|  |  | Indoor dampness |  | Registry | RR | Non-linear | Multiple studies |  |  |
| Lai et al | 2004 | Alcohol | Estonia | Cohort study | RR | NR | Multiple studies | Different scenarios | PAF |
|  |  | Smoking |  | Registry |  |  | Multiple studies |  |  |
|  |  | High BMI |  | Cohort study |  |  | Single study |  |  |
|  |  | Risky sexual behaviour |  | Literature |  |  | Single study |  |  |
|  |  | Drug use |  | Literature |  |  | Single study |  |  |
|  |  | Low fruit and vegetable intake |  | Cohort study |  |  | Single study |  |  |
|  |  | Physical inactivity |  | Cohort study |  |  | Single study |  |  |
| Lehtomäki et al | 2018 | Air pollution | Finland | Meteorological stations + model | RR | Exponential | Single study | Category with the lowest risk | PAF |
| Lelieveld et al | 2019 | Air pollution | Europe | Meteorological stations + model | Hazard ratio functions | Non-linear | Own calculation | NR | PAF |
| Lock et al | 2005 | Dietary risk | Global | Survey | RR | Non-Linear | Own calculation | 600 g person -1 day | PAF |
| Marmet et al | 2014 | Alcohol use | Switzerland | Survey | RR | Gamma function | GBD | Category with the lowest risk | PAF |
| Martín-Ramiro et al | 2014 | High BMI | Spain | Survey | RR | Linear | Meta-analysis | Category with the lowest risk | PAF |
| Möller et al | 2012 | Smoking | UK | Literature | RR | Linear | Meta-analysis | Different scenarios | PAF |
|  |  | High BMI |  | Literature | RR | Linear | Meta-analysis |  |  |
|  |  | Low fruit and vegetable intake |  | Literature | RR | Linear | Meta-analysis |  |  |
|  |  | Alcohol use |  | Literature | RR | Linear | Meta-analysis |  |  |
|  |  | Physical inactivity |  | Literature | RR | Linear | Meta-analysis |  |  |
| Öberg et al | 2010 | Second-hand smoking | Global | Survey, registry | RR | Linear | Meta-analysis | Category with the lowest risk | PAF |
| Öberg et al | 2011 | Second-hand smoking | Global | Survey/literature/modelling | RR/OR | Linear | Meta-analysis | NR | PAF |
| Oberoi et al | 2019 | Arsenic | Global | Literature | RR | Combination of functions | Multiple studies | Category with the lowest risk | PAF |
| Orru et al | 2011 | Air pollution | Estonia | Cohort study | RR | Linear | Meta-analysis | 0 (no PM_2,5_) PM_10_ increase by 10 µg/m^3^ | PAF |
| Papadimitriou et al | 2017 | Smoking | Europe, USA | Cohort study | HR | Cox model | Own calculation | Category with the lowest expected population risk | PAF |
|  |  | Alcohol use |  |  |  |  |  |  |  |
|  |  | High BMI |  |  |  |  |  |  |  |
|  |  | Type 2 diabetes |  |  |  |  |  |  |  |
|  |  | Physical inactivity |  |  |  |  |  |  |  |
|  |  | Use of hormone replacement therapy |  |  |  |  |  |  |  |
|  |  | Oral contraceptives |  |  |  |  |  |  |  |
| Paunovic et al | 2014 | Road traffic noise | Belgrade, Serbia | Cohort study | OR | Exponential | Own calculation | Category with the lowest risk | PAF |
| Pomerleau et al | 2006 | Dietary risk | EU-15 and EU-10 | Survey | RR | Linear | Meta-analysis | 400 g person -1 day -1 600 g person -1 day -1 | PAF |
| Rehm et al | 2007 | Alcohol | Switzerland | Survey | RR | Linear | Multiple studies | Category with the lowest risk | PAF |
| Rehm et al (53) | 2012 | Alcohol | Europe Union countries, plus Iceland, Norway, and Switzerland, plus Russia as an external comparison | Survey | RR | Exponential | Multiple studies | Different interventions | PAF |
| Rovira et al | 2020 | Air pollution | Catalonia, Spain | Registry | RR | NR | NR | Category with the lowest risk | PAF |
| Savolahti et al | 2019 | Air pollution | Finland | Modelling | RR | Log-linear | Own estimation | Category with the lowest risk | PAF |
| Schwingshackl et al | 2019 | Dietary risk | Europe | Meta-analysis | RR | Non-linear | Own estimation | Different optimal levels | PAF |
| Shield et al | 2013 | Alcohol | Italy | Survey | RR | Linear | Multiple studies | Category with the lowest risk | PAF (alcohol-attributable fraction) |
| Shield et al | 2015 | Alcohol | Russia | Literature | RR | Linear | Meta-analysis | Category with the lowest risk | PAF |
| Siddiqi et al (59) | 2015 | Smokeless tobacco | Global | Survey | RR | Linear | Systematic reviews and meta-analyses | Category with the lowest risk | PAF |
| Sifaki-Pistolla et al | 2017 | Smoking | Crete, Greece | Registry | RR | NR | NR | NA | PAF |
| Šipetić et al | 2013 | Alcohol use | Serbia without Kosovo and Metohia | Survey | RR | Linear | Multiple studies | Category with the lowest risk | PAF |
|  |  | Smoking |  |  |  |  |  |  |  |
|  |  | Physical inactivity |  |  |  |  |  |  |  |
|  |  | Dietary risk |  |  |  |  |  |  |  |
|  |  | High BMI |  |  |  |  |  |  |  |
|  |  | High blood cholesterol |  |  |  |  |  |  |  |
|  |  | Hypertension |  |  |  |  |  |  |  |
| Tobollik et al | 2019 | Road traffic noise | Germany | Literature | RR | Linear | Systematic review | 53 dB Lden | PAF |
|  |  | Aircraft noise |  |  |  | Exponential |  |  |  |
|  |  | Railway noise |  |  |  | Exponential |  |  |  |
| Tod et al | 2018 | Alcohol | Scotland | Survey | RR | Non-linear | Multiple studies | Category with the lowest risk | PAF |
| Tod et al | 2019 | Smoking | Scotland | Survey | HR | Cox model | Single study | Minimum health risk to a population | PAF |
|  |  | Alcohol use |  |  |  |  |  |  |  |
|  |  | High BMI |  |  |  |  |  |  |  |
|  |  | Low fruit and vegetable intake |  |  |  |  |  |  |  |
|  |  | Physical inactivity |  |  |  |  |  |  |  |
|  |  | High blood cholesterol |  |  |  |  |  |  |  |
|  |  | Hypertension |  |  |  |  |  |  |  |
| Tsilidis et al | 2016 | Smoking | Denmark, Greece, The Netherlands, Spain, Sweden, Germany, Norway and UK | Survey | RR and HR | Cox model | Own calculation | NR | PAF |
|  |  | High BMI |  |  |  |  |  |  |  |
|  |  | Physical inactivity |  |  |  |  |  |  |  |
|  |  | Alcohol use |  |  |  |  |  |  |  |
|  |  | Type 2 diabetes |  |  |  |  |  |  |  |
| Valent et al | 2004 | Outdoor air pollution | 51 countries WHO European region | Literature | RR | Linear | Single study | PM_10_ concentration: 10g/m^3^ and 20g/m^3^ | PAF (Impact fraction) |
|  |  | Indoor air pollution |  |  |  |  |  | No households used solid fuel |  |
|  |  | Inadequate water and sanitation |  |  |  |  |  | 100% have no transmission of diarrhoeal disease |  |
|  |  | Lead |  |  |  |  |  | Blood lead concentration in preindustrial people 0·16 g/L |  |
| Van Kreijl et al | 2006 | Dietary risk | The Netherlands | Multiple studies | RR | Linear | Multiple studies | Category with the lowest risk and different interventions | PAF |
| Vienneau et al | 2015 | Spatially resolved noise | Switzerland | Modelling | RR | Exponential | Multiple studies | Category with the lowest risk | PAF |
|  |  | Air pollution |  | Modelling |  | Exponential | Multiple studies | Category with the lowest risk |  |

RR: relative risk; OR: odds ratio; ERF: exposure-response function; PAF: population attributable fraction; NA: not applicable; NR: not reported

[1] B. Ádám, Á. Molnár, G. Gulis, and R. Ádány, “Integrating a quantitative risk appraisal in a health impact assessment: analysis of the novel smoke-free policy in Hungary,” *Eur. J. Public Health*, vol. 23, no. 2, pp. 211–217, Apr. 2013, doi: 10.1093/eurpub/cks018.

[2] E. E. Agardh *et al.*, “Burden of type 2 diabetes attributed to lower educational levels in Sweden,” *Popul. Health Metr.*, vol. 9, p. 60, Dec. 2011, doi: 10.1186/1478-7954-9-60.

[3] E. E. Agardh *et al.*, “Alcohol-attributed disease burden in four Nordic countries: a comparison using the Global Burden of Disease, Injuries and Risk Factors 2013 study,” *Addict. Abingdon Engl.*, vol. 111, no. 10, pp. 1806–1813, Oct. 2016, doi: 10.1111/add.13430.

[4] E. Agardh, T. Moradi, and P. Allebeck, “[The contribution of risk factors to the burden of disease in Sweden. A comparison between Swedish and WHO data],” *Lakartidningen*, vol. 105, no. 11, pp. 816–821, Mar. 2008.

[5] S. Allender and M. Rayner, “The burden of overweight and obesity-related ill health in the UK,” *Obes. Rev. Off. J. Int. Assoc. Study Obes.*, vol. 8, no. 5, pp. 467–473, Sep. 2007, doi: 10.1111/j.1467-789X.2007.00394.x.

[6] S. Allender, R. Balakrishnan, P. Scarborough, P. Webster, and M. Rayner, “The burden of smoking-related ill health in the UK,” *Tob. Control*, vol. 18, no. 4, pp. 262–267, Aug. 2009, doi: 10.1136/tc.2008.026294.

[7] S. Allender, C. Foster, P. Scarborough, and M. Rayner, “The burden of physical activity-related ill health in the UK,” *J. Epidemiol. Community Health*, vol. 61, no. 4, pp. 344–348, Apr. 2007, doi: 10.1136/jech.2006.050807.

[8] S. S. Babatola, “Global burden of diseases attributable to air pollution,” *J. Public Health Afr.*, vol. 9, no. 3, p. 813, Dec. 2018, doi: 10.4081/jphia.2018.813.

[9] R. Balakrishnan, S. Allender, P. Scarborough, P. Webster, and M. Rayner, “The burden of alcohol-related ill health in the United Kingdom,” *J. Public Health Oxf. Engl.*, vol. 31, no. 3, pp. 366–373, Sep. 2009, doi: 10.1093/pubmed/fdp051.

[10] P. Begou, P. Kassomenos, and A. Kelessis, “Effects of road traffic noise on the prevalence of cardiovascular diseases: The case of Thessaloniki, Greece,” *Sci. Total Environ.*, vol. 703, p. 134477, Feb. 2020, doi: 10.1016/j.scitotenv.2019.134477.

[11] L. M. Bello, P. Saavedra, and L. Serra, “[Trends in mortality and years of life lost related to alcohol in the Canary Islands, Spain [1980-1998]],” *Gac. Sanit.*, vol. 17, no. 6, pp. 466–473, 2003, doi: 10.1016/s0213-9111(03)71793-2.

[12] D. A. Bennett *et al.*, “The global burden of ischemic stroke: findings of the GBD 2010 study,” *Glob. Heart*, vol. 9, no. 1, pp. 107–112, Mar. 2014, doi: 10.1016/j.gheart.2014.01.001.

[13] M. Borges, M. Gouveia, J. Costa, L. Dos Santos Pinheiro, S. Paulo, and A. Vaz Carneiro, “The burden of disease attributable to smoking in Portugal,” *Rev. Port. Pneumol.*, vol. 15, no. 6, pp. 951–1004, 2009.

[14] B. Bowe, Y. Xie, T. Li, Y. Yan, H. Xian, and Z. Al-Aly, “Estimates of the 2016 global burden of kidney disease attributable to ambient fine particulate matter air pollution,” *BMJ Open*, vol. 9, no. 5, p. e022450, May 2019, doi: 10.1136/bmjopen-2018-022450.

[15] E. W. Butt *et al.*, “Global and regional trends in particulate air pollution and attributable health burden over the past 50 years,” *Environ. Res. Lett.*, vol. 12, no. 10, p. 104017, Oct. 2017, doi: 10.1088/1748-9326/aa87be.

[16] G. Carreras *et al.*, “Burden of disease from breast cancer attributable to smoking and second-hand smoke exposure in Europe,” *Int. J. Cancer*, vol. 147, no. 9, pp. 2387–2393, Nov. 2020, doi: 10.1002/ijc.33021.

[17] A. J. Cohen *et al.*, “Estimates and 25-year trends of the global burden of disease attributable to ambient air pollution: an analysis of data from the Global Burden of Diseases Study 2015,” *Lancet Lond. Engl.*, vol. 389, no. 10082, pp. 1907–1918, May 2017, doi: 10.1016/S0140-6736(17)30505-6.

[18] GBD 2016 Risk Factors Collaborators, “Global, regional, and national comparative risk assessment of 84 behavioural, environmental and occupational, and metabolic risks or clusters of risks, 1990-2016: a systematic analysis for the Global Burden of Disease Study 2016,” *Lancet Lond. Engl.*, vol. 390, no. 10100, pp. 1345–1422, Sep. 2017, doi: 10.1016/S0140-6736(17)32366-8.

[19] GBD 2015 Risk Factors Collaborators, “Global, regional, and national comparative risk assessment of 79 behavioural, environmental and occupational, and metabolic risks or clusters of risks, 1990-2015: a systematic analysis for the Global Burden of Disease Study 2015,” *Lancet Lond. Engl.*, vol. 388, no. 10053, pp. 1659–1724, Oct. 2016, doi: 10.1016/S0140-6736(16)31679-8.

[20] H. Cortez-Pinto, M. Gouveia, L. dos Santos Pinheiro, J. Costa, M. Borges, and A. Vaz Carneiro, “The burden of disease and the cost of illness attributable to alcohol drinking--results of a national study,” *Alcohol. Clin. Exp. Res.*, vol. 34, no. 8, pp. 1442–1449, Aug. 2010, doi: 10.1111/j.1530-0277.2010.01229.x.

[21] J. De Oliveira Mota, G. Boué, S. Guillou, F. Pierre, and J.-M. Membré, “Estimation of the burden of disease attributable to red meat consumption in France: Influence on colorectal cancer and cardiovascular diseases,” *Food Chem. Toxicol. Int. J. Publ. Br. Ind. Biol. Res. Assoc.*, vol. 130, pp. 174–186, Aug. 2019, doi: 10.1016/j.fct.2019.05.023.

[22] L. Degenhardt *et al.*, “The global epidemiology and burden of psychostimulant dependence: findings from the Global Burden of Disease Study 2010,” *Drug Alcohol Depend.*, vol. 137, pp. 36–47, Apr. 2014, doi: 10.1016/j.drugalcdep.2013.12.025.

[23] L. Degenhardt *et al.*, “Estimating the burden of disease attributable to injecting drug use as a risk factor for HIV, hepatitis C, and hepatitis B: findings from the Global Burden of Disease Study 2013,” *Lancet Infect. Dis.*, vol. 16, no. 12, pp. 1385–1398, Dec. 2016, doi: 10.1016/S1473-3099(16)30325-5.

[24] L. Degenhardt *et al.*, “Global burden of disease attributable to illicit drug use and dependence: findings from the Global Burden of Disease Study 2010,” *Lancet Lond. Engl.*, vol. 382, no. 9904, pp. 1564–1574, Nov. 2013, doi: 10.1016/S0140-6736(13)61530-5.

[25] Direção-Geral da Saúde, Institute for Health Metrics and Evaluation., “Portugal: The Nation’s Health 1990–2016: An overview of the Global Burden of Disease Study 2016 Results.,” *Seattle, WA: IHME, 2018*, Apr. 04, 2018. https://www.healthdata.org/policy-report/portugal-nation%E2%80%99s-health-1990%E2%80%932016 (accessed Feb. 05, 2023).

[26] GBD 2016 Occupational Chronic Respiratory Risk Factors Collaborators and GBD 2016 occupational chronic respiratory risk factors collaborators, “Global and regional burden of chronic respiratory disease in 2016 arising from non-infectious airborne occupational exposures: a systematic analysis for the Global Burden of Disease Study 2016,” *Occup. Environ. Med.*, vol. 77, no. 3, pp. 142–150, Mar. 2020, doi: 10.1136/oemed-2019-106013.

[27] T. Driscoll *et al.*, “The global burden of disease due to occupational carcinogens,” *Am. J. Ind. Med.*, vol. 48, no. 6, pp. 419–431, Dec. 2005, doi: 10.1002/ajim.20209.

[28] A. Dzhambov and D. Dimitrova, “Road traffic noise and annoyance: exposure-response relationship and burden of disease calculations in Bulgaria,” *Scr. Sci. Medica*, vol. 47, no. 2, p. 22, Jun. 2015, doi: 10.14748/ssm.v47i2.1153.

[29] T. Effertz and K. Mann, “The burden and cost of disorders of the brain in Europe with the inclusion of harmful alcohol use and nicotine addiction,” *Eur. Neuropsychopharmacol. J. Eur. Coll. Neuropsychopharmacol.*, vol. 23, no. 7, pp. 742–748, Jul. 2013, doi: 10.1016/j.euroneuro.2012.07.010.

[30] M. Ezzati, A. D. Lopez, A. Rodgers, S. Vander Hoorn, C. J. L. Murray, and Comparative Risk Assessment Collaborating Group, “Selected major risk factors and global and regional burden of disease,” *Lancet Lond. Engl.*, vol. 360, no. 9343, pp. 1347–1360, Nov. 2002, doi: 10.1016/S0140-6736(02)11403-6.

[31] GBD 2015 Neurological Disorders Collaborator Group, “Global, regional, and national burden of neurological disorders during 1990-2015: a systematic analysis for the Global Burden of Disease Study 2015,” *Lancet Neurol.*, vol. 16, no. 11, pp. 877–897, Nov. 2017, doi: 10.1016/S1474-4422(17)30299-5.

[32] A. J. Ferrari *et al.*, “Burden of depressive disorders by country, sex, age, and year: findings from the global burden of disease study 2010,” *PLoS Med.*, vol. 10, no. 11, p. e1001547, Nov. 2013, doi: 10.1371/journal.pmed.1001547.

[33] A. J. Ferrari *et al.*, “The burden attributable to mental and substance use disorders as risk factors for suicide: findings from the Global Burden of Disease Study 2010,” *PloS One*, vol. 9, no. 4, p. e91936, 2014, doi: 10.1371/journal.pone.0091936.

[34] GBD 2013 Risk Factors Collaborators *et al.*, “Global, regional, and national comparative risk assessment of 79 behavioural, environmental and occupational, and metabolic risks or clusters of risks in 188 countries, 1990-2013: a systematic analysis for the Global Burden of Disease Study 2013,” *Lancet Lond. Engl.*, vol. 386, no. 10010, pp. 2287–2323, Dec. 2015, doi: 10.1016/S0140-6736(15)00128-2.

[35] GBD 2016 Alcohol and Drug Use Collaborators, “The global burden of disease attributable to alcohol and drug use in 195 countries and territories, 1990-2016: a systematic analysis for the Global Burden of Disease Study 2016,” *Lancet Psychiatry*, vol. 5, no. 12, pp. 987–1012, Dec. 2018, doi: 10.1016/S2215-0366(18)30337-7.

[36] GBD 2016 Occupational Carcinogens Collaborators, “Global and regional burden of cancer in 2016 arising from occupational exposure to selected carcinogens: a systematic analysis for the Global Burden of Disease Study 2016,” *Occup. Environ. Med.*, vol. 77, no. 3, pp. 151–159, Mar. 2020, doi: 10.1136/oemed-2019-106012.

[37] GBD 2017 Disease and Injury Incidence and Prevalence Collaborators, “Global, regional, and national incidence, prevalence, and years lived with disability for 354 diseases and injuries for 195 countries and territories, 1990-2017: a systematic analysis for the Global Burden of Disease Study 2017,” *Lancet Lond. Engl.*, vol. 392, no. 10159, pp. 1789–1858, Nov. 2018, doi: 10.1016/S0140-6736(18)32279-7.

[38] M. Gouveia, M. Borges, J. Costa, and A. V. Carneiro, “Burden of disease from hypercholesterolemia in Portugal,” *Rev. Port. Cardiol. Orgao Of. Soc. Port. Cardiol. Port. J. Cardiol. Off. J. Port. Soc. Cardiol.*, vol. 23, no. 2, pp. 255–270, Feb. 2004.

[39] P. Grandjean and M. Bellanger, “Calculation of the disease burden associated with environmental chemical exposures: application of toxicological information in health economic estimation,” *Environ. Health Glob. Access Sci. Source*, vol. 16, no. 1, p. 123, Dec. 2017, doi: 10.1186/s12940-017-0340-3.

[40] Y. Guillois-Becel, D. Eilstein, Ph. Glorennec, and A. Lefranc, “Quantification of years of life lost attributable to chronic air pollution exposure in a health impact assessment: the case of Nantes,” *Environ. Risques Sante*, vol. 6, no. 3, pp. 189–197, 2007.

[41] O. Hänninen *et al.*, “Environmental burden of disease in Europe: assessing nine risk factors in six countries,” *Environ. Health Perspect.*, vol. 122, no. 5, pp. 439–446, May 2014, doi: 10.1289/ehp.1206154.

[42] P. Holnicki, M. Tainio, A. Kałuszko, and Z. Nahorski, “Burden of Mortality and Disease Attributable to Multiple Air Pollutants in Warsaw, Poland,” *Int. J. Environ. Res. Public. Health*, vol. 14, no. 11, p. E1359, Nov. 2017, doi: 10.3390/ijerph14111359.

[43] L. S. Jakobsen, K. Granby, V. K. Knudsen, M. Nauta, S. M. Pires, and M. Poulsen, “Burden of disease of dietary exposure to acrylamide in Denmark,” *Food Chem. Toxicol. Int. J. Publ. Br. Ind. Biol. Res. Assoc.*, vol. 90, pp. 151–159, Apr. 2016, doi: 10.1016/j.fct.2016.01.021.

[44] D. Jarosińska, K. Polańska, B. Wojtyniak, and W. Hanke, “Towards estimating the burden of disease attributable to second-hand smoke exposure in Polish children,” *Int. J. Occup. Med. Environ. Health*, vol. 27, no. 1, pp. 38–49, Jan. 2014, doi: 10.2478/s13382-014-0223-6.

[45] P. A. Kassomenos, K. Dimitriou, and A. K. Paschalidou, “Human health damage caused by particulate matter PM10 and ozone in urban environments: the case of Athens, Greece,” *Environ. Monit. Assess.*, vol. 185, no. 8, pp. 6933–6942, Aug. 2013, doi: 10.1007/s10661-013-3076-8.

[46] M. D. Keall, D. Ormandy, and M. G. Baker, “Injuries associated with housing conditions in Europe: a burden of disease study based on 2004 injury data,” *Environ. Health Glob. Access Sci. Source*, vol. 10, p. 98, Nov. 2011, doi: 10.1186/1476-069X-10-98.

[47] K. Kellerborg, A.-K. Danielsson, P. Allebeck, M. M. Coates, and E. Agardh, “Disease burden attributed to alcohol: How methodological advances in the Global Burden of Disease 2013 study have changed the estimates in Sweden,” *Scand. J. Public Health*, vol. 44, no. 6, pp. 604–610, Aug. 2016, doi: 10.1177/1403494816653512.

[48] AB Knol and BAM Staatsen, “Trends in the environmental burden of disease in the Netherlands 1980 – 2020,” 500029001/2005. [Online]. Available: https://www.rivm.nl/bibliotheek/rapporten/500029001.pdf

[49] Nordic Burden of Disease Collaborators, “Life expectancy and disease burden in the Nordic countries: results from the Global Burden of Diseases, Injuries, and Risk Factors Study 2017,” *Lancet Public Health*, vol. 4, no. 12, pp. e658–e669, Dec. 2019, doi: 10.1016/S2468-2667(19)30224-5.

[50] Ann Kristin Knudsen, Jonas Minet Kinge, Vegard Skirbekk, and Stein Emil Vollset, “Sykdomsbyrde i Norge 1990–2013,” Bergen/Oslo: Folkehelseinstituttet, 2016, 2016:1. [Online]. Available: fhi.no/publ/2016/sykdomsbyrde-i-norge-1990-2013/#:~:text=Til%20tross%20for%20at%20befolkningen,leveår%20som%20følge%20av%20hjertesykdom.

[51] Knudsen AK, Tollånes MC, Haaland ØA, Kinge JM, Skirbekk V, Vollset SE, “Sykdomsbyrde i Norge 2015. Resultater fra Global Burden of Diseases, Injuries, and Risk Factors Study 2015 (GBD 2015),” Bergen/Oslo: Folkehelseinstituttet, 2017., Rapport 2017. [Online]. Available: https://www.fhi.no/publ/2017/sykdomsbyrde-i-norge-2015/#:~:text=Forventet%20levealder%20ved%20f%C3%B8dsel%20i,%2Dd%C3%B8delig%20helsetap%20(sykelighet).

[52] T. R. Ülikool, “Haiguskoormuse tõttu kaotatud eluaastad Eestis: seosed riskifaktoritega ja riskide vähendamise kulutõhusus,” 2004.

[53] C. M. M. Lawes, S. Vander Hoorn, M. R. Law, P. Elliott, S. MacMahon, and A. Rodgers, “Blood pressure and the global burden of disease 2000. Part II: estimates of attributable burden,” *J. Hypertens.*, vol. 24, no. 3, pp. 423–430, Mar. 2006, doi: 10.1097/01.hjh.0000209973.67746.f0.

[54] H. Lehtomäki *et al.*, “Health Impacts of Ambient Air Pollution in Finland,” *Int. J. Environ. Res. Public. Health*, vol. 15, no. 4, p. 736, Apr. 2018, doi: 10.3390/ijerph15040736.

[55] J. Lelieveld *et al.*, “Cardiovascular disease burden from ambient air pollution in Europe reassessed using novel hazard ratio functions,” *Eur. Heart J.*, vol. 40, no. 20, pp. 1590–1596, May 2019, doi: 10.1093/eurheartj/ehz135.

[56] X. Li, X. Cao, M. Guo, M. Xie, and X. Liu, “Trends and risk factors of mortality and disability adjusted life years for chronic respiratory diseases from 1990 to 2017: systematic analysis for the Global Burden of Disease Study 2017,” *BMJ*, vol. 368, p. m234, Feb. 2020, doi: 10.1136/bmj.m234.

[57] S. S. Lim *et al.*, “A comparative risk assessment of burden of disease and injury attributable to 67 risk factors and risk factor clusters in 21 regions, 1990-2010: a systematic analysis for the Global Burden of Disease Study 2010,” *Lancet Lond. Engl.*, vol. 380, no. 9859, pp. 2224–2260, Dec. 2012, doi: 10.1016/S0140-6736(12)61766-8.

[58] K. Lock, J. Pomerleau, L. Causer, D. R. Altmann, and M. McKee, “The global burden of disease attributable to low consumption of fruit and vegetables: implications for the global strategy on diet,” *Bull. World Health Organ.*, vol. 83, no. 2, pp. 100–108, Feb. 2005.

[59] A. D. Lopez, C. D. Mathers, M. Ezzati, D. T. Jamison, and C. J. L. Murray, “Global and regional burden of disease and risk factors, 2001: systematic analysis of population health data,” *Lancet Lond. Engl.*, vol. 367, no. 9524, pp. 1747–1757, May 2006, doi: 10.1016/S0140-6736(06)68770-9.

[60] S. Marmet, J. Rehm, and G. Gmel, “The importance of age groups in estimates of alcohol-attributable mortality: impact on trends in Switzerland between 1997 and 2011,” *Addict. Abingdon Engl.*, vol. 111, no. 2, pp. 255–262, Feb. 2016, doi: 10.1111/add.13164.

[61] J. J. Martín-Ramiro, E. Alvarez-Martín, and R. Gil-Prieto, “[Disability attributable to excess weight in Spain],” *Med. Clin. (Barc.)*, vol. 143, no. 4, pp. 150–156, Aug. 2014, doi: 10.1016/j.medcli.2013.05.028.

[62] A. M. May *et al.*, “The impact of a healthy lifestyle on Disability-Adjusted Life Years: a prospective cohort study,” *BMC Med.*, vol. 13, p. 39, Feb. 2015, doi: 10.1186/s12916-015-0287-6.

[63] T. Meier, P. Deumelandt, O. Christen, G. I. Stangl, K. Riedel, and M. Langer, “Global Burden of Sugar-Related Dental Diseases in 168 Countries and Corresponding Health Care Costs,” *J. Dent. Res.*, vol. 96, no. 8, pp. 845–854, Jul. 2017, doi: 10.1177/0022034517708315.

[64] T. Meier *et al.*, “Cardiovascular mortality attributable to dietary risk factors in 51 countries in the WHO European Region from 1990 to 2016: a systematic analysis of the Global Burden of Disease Study,” *Eur. J. Epidemiol.*, vol. 34, no. 1, pp. 37–55, Jan. 2019, doi: 10.1007/s10654-018-0473-x.

[65] H. Meijerink *et al.*, “Modelling the burden of hepatitis C infection among people who inject drugs in Norway, 1973-2030,” *BMC Infect. Dis.*, vol. 17, no. 1, p. 541, Aug. 2017, doi: 10.1186/s12879-017-2631-2.

[66] T. Miazgowski, A. Taszarek, K. Widecka, B. Miazgowski, and K. Homa, “Deaths, disability-adjusted life years and years of life lost due to elevated systolic blood pressure in Poland: estimates for the Global Burden of Disease Study 2016,” *Arter. Hypertens.*, vol. 22, no. 2, pp. 95–103, Jun. 2018, doi: 10.5603/AH.a2018.0005.

[67] A. H. Mokdad *et al.*, “Global burden of diseases, injuries, and risk factors for young people’s health during 1990-2013: a systematic analysis for the Global Burden of Disease Study 2013,” *Lancet Lond. Engl.*, vol. 387, no. 10036, pp. 2383–2401, Jun. 2016, doi: 10.1016/S0140-6736(16)00648-6.

[68] H. Möller, M. Dherani, C. Harwood, T. Kinsella, and D. Pope, “Health planning for the future: comparative risk assessment of five major lifestyle risk factors: evidence from the Wirral, UK,” *J. Public Health Oxf. Engl.*, vol. 34, no. 3, pp. 430–437, Aug. 2012, doi: 10.1093/pubmed/fds005.

[69] GBD 2017 Italy Collaborators, “Italy’s health performance, 1990-2017: findings from the Global Burden of Disease Study 2017,” *Lancet Public Health*, vol. 4, no. 12, pp. e645–e657, Dec. 2019, doi: 10.1016/S2468-2667(19)30189-6.

[70] A. Murphy *et al.*, “Ischaemic heart disease in the former Soviet Union 1990-2015 according to the Global Burden of Disease 2015 Study,” *Heart Br. Card. Soc.*, vol. 104, no. 1, pp. 58–66, Jan. 2018, doi: 10.1136/heartjnl-2016-311142.

[71] C. J. L. Murray *et al.*, “UK health performance: findings of the Global Burden of Disease Study 2010,” *Lancet Lond. Engl.*, vol. 381, no. 9871, pp. 997–1020, Mar. 2013, doi: 10.1016/S0140-6736(13)60355-4.

[72] J. N. Newton *et al.*, “Changes in health in England, with analysis by English regions and areas of deprivation, 1990-2013: a systematic analysis for the Global Burden of Disease Study 2013,” *Lancet Lond. Engl.*, vol. 386, no. 10010, pp. 2257–2274, Dec. 2015, doi: 10.1016/S0140-6736(15)00195-6.

[73] M. Oberg, M. S. Jaakkola, A. Woodward, A. Peruga, and A. Prüss-Ustün, “Worldwide burden of disease from exposure to second-hand smoke: a retrospective analysis of data from 192 countries,” *Lancet Lond. Engl.*, vol. 377, no. 9760, pp. 139–146, Jan. 2011, doi: 10.1016/S0140-6736(10)61388-8.

[74] M. Öberg, M. S. Jaakkola, A. Prüss-Üstün, A. Peruga, A. Woodward, and World Health Organization, “Global estimate of the burden of disease from second-hand smoke / by Mattias Öberg ... [et al],” 2010, [Online]. Available: https://apps.who.int/iris/handle/10665/44426

[75] S. Oberoi, B. Devleesschauwer, H. J. Gibb, and A. Barchowsky, “Global burden of cancer and coronary heart disease resulting from dietary exposure to arsenic, 2015,” *Environ. Res.*, vol. 171, pp. 185–192, Apr. 2019, doi: 10.1016/j.envres.2019.01.025.

[76] H. Orru *et al.*, “Health impact assessment of particulate pollution in Tallinn using fine spatial resolution and modeling techniques,” *Environ. Health Glob. Access Sci. Source*, vol. 8, p. 7, Mar. 2009, doi: 10.1186/1476-069X-8-7.

[77] M. C. Tollånes, A. K. Knudsen, S. E. Vollset, J. M. Kinge, V. Skirbekk, and S. Øverland, “Sykdomsbyrden i Norge i 2016,” *Tidsskr. Den Nor. Legeforening*, 2018, doi: 10.4045/tidsskr.18.0274.

[78] N. Papadimitriou *et al.*, “Burden of hip fracture using disability-adjusted life-years: a pooled analysis of prospective cohorts in the CHANCES consortium,” *Lancet Public Health*, vol. 2, no. 5, pp. e239–e246, May 2017, doi: 10.1016/S2468-2667(17)30046-4.

[79] K. Paunovic and G. Belojević, “Burden of myocardial infarction attributable to road-traffic noise: a pilot study in Belgrade,” *Noise Health*, vol. 16, no. 73, pp. 374–379, Dec. 2014, doi: 10.4103/1463-1741.144415.

[80] J. Pomerleau, K. Lock, and M. McKee, “The burden of cardiovascular disease and cancer attributable to low fruit and vegetable intake in the European Union: differences between old and new Member States,” *Public Health Nutr.*, vol. 9, no. 5, pp. 575–583, Aug. 2006, doi: 10.1079/phn2005910.

[81] J. W. Powles, W. Zatonski, S. Vander Hoorn, and M. Ezzati, “The contribution of leading diseases and risk factors to excess losses of healthy life in Eastern Europe: burden of disease study,” *BMC Public Health*, vol. 5, p. 116, Nov. 2005, doi: 10.1186/1471-2458-5-116.

[82] M. Rayner and P. Scarborough, “The burden of food related ill health in the UK,” *J. Epidemiol. Community Health*, vol. 59, no. 12, pp. 1054–1057, Dec. 2005, doi: 10.1136/jech.2005.036491.

[83] J. Rehm, K. D. Shield, M. X. Rehm, G. Gmel, and U. Frick, “Alcohol consumption, alcohol dependence, and attributable burden of disease in Europe: Potential gains from effective interventions for alcohol dependence,” 2012, doi: 10.5167/UZH-64919.

[84] J. Rehm, B. Taylor, M. Roerecke, and J. Patra, “Alcohol consumption and alcohol-attributable burden of disease in Switzerland, 2002,” *Int. J. Public Health*, vol. 52, no. 6, pp. 383–392, Dec. 2007, doi: 10.1007/s00038-007-7010-0.

[85] J. Rehm, J. Manthey, K. D. Shield, and C. Ferreira-Borges, “Trends in substance use and in the attributable burden of disease and mortality in the WHO European Region, 2010-16,” *Eur. J. Public Health*, vol. 29, no. 4, pp. 723–728, Aug. 2019, doi: 10.1093/eurpub/ckz064.

[86] J. Rehm, C. Mathers, S. Popova, M. Thavorncharoensap, Y. Teerawattananon, and J. Patra, “Global burden of disease and injury and economic cost attributable to alcohol use and alcohol-use disorders,” *Lancet Lond. Engl.*, vol. 373, no. 9682, pp. 2223–2233, Jun. 2009, doi: 10.1016/S0140-6736(09)60746-7.

[87] GBD 2015 Tobacco Collaborators, “Smoking prevalence and attributable disease burden in 195 countries and territories, 1990-2015: a systematic analysis from the Global Burden of Disease Study 2015,” *Lancet Lond. Engl.*, vol. 389, no. 10082, pp. 1885–1906, May 2017, doi: 10.1016/S0140-6736(17)30819-X.

[88] J. Rovira, J. L. Domingo, and M. Schuhmacher, “Air quality, health impacts and burden of disease due to air pollution (PM10, PM2.5, NO2 and O3): Application of AirQ+ model to the Camp de Tarragona County (Catalonia, Spain),” *Sci. Total Environ.*, vol. 703, p. 135538, Feb. 2020, doi: 10.1016/j.scitotenv.2019.135538.

[89] GBD 2017 Colorectal Cancer Collaborators, “The global, regional, and national burden of colorectal cancer and its attributable risk factors in 195 countries and territories, 1990-2017: a systematic analysis for the Global Burden of Disease Study 2017,” *Lancet Gastroenterol. Hepatol.*, vol. 4, no. 12, pp. 913–933, Dec. 2019, doi: 10.1016/S2468-1253(19)30345-0.

[90] M. Savolahti *et al.*, “Residential Wood Combustion in Finland: PM2.5 Emissions and Health Impacts with and without Abatement Measures,” *Int. J. Environ. Res. Public. Health*, vol. 16, no. 16, p. E2920, Aug. 2019, doi: 10.3390/ijerph16162920.

[91] L. Schwingshackl *et al.*, “Intake of 12 food groups and disability-adjusted life years from coronary heart disease, stroke, type 2 diabetes, and colorectal cancer in 16 European countries,” *Eur. J. Epidemiol.*, vol. 34, no. 8, pp. 765–775, Aug. 2019, doi: 10.1007/s10654-019-00523-4.

[92] K. D. Shield, J. Rehm, G. Gmel, M. X. Rehm, and A. Allamani, “Alcohol consumption, alcohol dependence, and related mortality in Italy in 2004: effects of treatment-based interventions on alcohol dependence,” *Subst. Abuse Treat. Prev. Policy*, vol. 8, p. 21, Jun. 2013, doi: 10.1186/1747-597X-8-21.

[93] K. D. Shield and J. Rehm, “Russia-specific relative risks and their effects on the estimated alcohol-attributable burden of disease,” *BMC Public Health*, vol. 15, p. 482, May 2015, doi: 10.1186/s12889-015-1818-y.

[94] K. Shield *et al.*, “National, regional, and global burdens of disease from 2000 to 2016 attributable to alcohol use: a comparative risk assessment study,” *Lancet Public Health*, vol. 5, no. 1, pp. e51–e61, Jan. 2020, doi: 10.1016/S2468-2667(19)30231-2.

[95] K. D. Shield, G. Gmel, J. Patra, and J. Rehm, “Global burden of injuries attributable to alcohol consumption in 2004: a novel way of calculating the burden of injuries attributable to alcohol consumption,” *Popul. Health Metr.*, vol. 10, no. 1, p. 9, May 2012, doi: 10.1186/1478-7954-10-9.

[96] K. Siddiqi *et al.*, “Global burden of disease due to smokeless tobacco consumption in adults: analysis of data from 113 countries,” *BMC Med.*, vol. 13, p. 194, Aug. 2015, doi: 10.1186/s12916-015-0424-2.

[97] D. Sifaki-Pistolla *et al.*, “Lung cancer and tobacco smoking in Crete, Greece: reflections from a population-based cancer registry from 1992 to 2013,” *Tob. Induc. Dis.*, vol. 15, p. 6, 2017, doi: 10.1186/s12971-017-0114-2.

[98] S. Sipetić *et al.*, “The burden of disease preventable by risk factor reduction in Serbia,” *Vojnosanit. Pregl.*, vol. 70, no. 5, pp. 445–451, May 2013, doi: 10.2298/vsp111024049s.

[99] GBD 2017 Disease and Injury Incidence and Prevalence Collaborators, “Global, regional, and national incidence, prevalence, and years lived with disability for 354 diseases and injuries for 195 countries and territories, 1990-2017: a systematic analysis for the Global Burden of Disease Study 2017,” *Lancet Lond. Engl.*, vol. 392, no. 10159, pp. 1789–1858, Nov. 2018, doi: 10.1016/S0140-6736(18)32279-7.

[100] N. Steel *et al.*, “Changes in health in the countries of the UK and 150 English Local Authority areas 1990-2016: a systematic analysis for the Global Burden of Disease Study 2016,” *Lancet Lond. Engl.*, vol. 392, no. 10158, pp. 1647–1661, Nov. 2018, doi: 10.1016/S0140-6736(18)32207-4.

[101] M. Tobollik, M. Hintzsche, J. Wothge, T. Myck, and D. Plass, “Burden of Disease Due to Traffic Noise in Germany,” *Int. J. Environ. Res. Public. Health*, vol. 16, no. 13, p. 2304, Jun. 2019, doi: 10.3390/ijerph16132304.

[102] E. Tod *et al.*, *Hospital admissions, deaths and overall burden of disease attributable to alcohol consumption in Scotland*. 2018.

[103] E. Tod *et al.*, “What causes the burden of stroke in Scotland? A comparative risk assessment approach linking the Scottish Health Survey to administrative health data,” *PloS One*, vol. 14, no. 7, p. e0216350, 2019, doi: 10.1371/journal.pone.0216350.

[104] GBD 2016 Lower Respiratory Infections Collaborators, “Estimates of the global, regional, and national morbidity, mortality, and aetiologies of lower respiratory infections in 195 countries, 1990-2016: a systematic analysis for the Global Burden of Disease Study 2016,” *Lancet Infect. Dis.*, vol. 18, no. 11, pp. 1191–1210, Nov. 2018, doi: 10.1016/S1473-3099(18)30310-4.

[105] K. K. Tsilidis *et al.*, “Burden of Cancer in a Large Consortium of Prospective Cohorts in Europe,” *J. Natl. Cancer Inst.*, vol. 108, no. 10, p. djw127, Oct. 2016, doi: 10.1093/jnci/djw127.

[106] Global Burden of Disease 2016 Greece Collaborators, “The burden of disease in Greece, health loss, risk factors, and health financing, 2000-16: an analysis of the Global Burden of Disease Study 2016,” *Lancet Public Health*, vol. 3, no. 8, pp. e395–e406, Aug. 2018, doi: 10.1016/S2468-2667(18)30130-0.

[107] F. Valent, D. Little, R. Bertollini, L. E. Nemer, F. Barbone, and G. Tamburlini, “Burden of disease attributable to selected environmental factors and injury among children and adolescents in Europe,” *Lancet Lond. Engl.*, vol. 363, no. 9426, pp. 2032–2039, Jun. 2004, doi: 10.1016/S0140-6736(04)16452-0.

[108] K. van, K. AGAC, R. van, and CVG, “Our food, our health-Healthy diet and safe food in the Netherlands,” Jan. 2006.

[109] D. Vienneau *et al.*, “Years of life lost and morbidity cases attributable to transportation noise and air pollution: A comparative health risk assessment for Switzerland in 2010,” *Int. J. Hyg. Environ. Health*, vol. 218, no. 6, pp. 514–521, Aug. 2015, doi: 10.1016/j.ijheh.2015.05.003.

[110] H. A. Whiteford *et al.*, “Global burden of disease attributable to mental and substance use disorders: findings from the Global Burden of Disease Study 2010,” *Lancet Lond. Engl.*, vol. 382, no. 9904, pp. 1575–1586, Nov. 2013, doi: 10.1016/S0140-6736(13)61611-6.

[111] World Health Organization, “The global burden of disease : 2004 update,” World Health Organization, 2008. Accessed: Feb. 05, 2023. [Online]. Available: https://apps.who.int/iris/handle/10665/43942
