# Supplementary material for: Burden of disease attributable to risk factors in European countries: a scoping literature review
Source: Arch Public Health. 2023 Jun 25;81:116. doi: 10.1186/s13690-023-01119-x (PMC10290804; doi:10.1186/s13690-023-01119-x)
Supplement: Supplementary file 5 — Additional file 5. Reference list of the included disease burden studies. [file 13690_2023_1119_MOESM5_ESM.docx]

**Additional file 5: Reference list of the included disease burden studies (N=113)**

| Author(s) | Year | Title | Country(s) / Region | Study type  (Independent or GBD-linked study) |
| --- | --- | --- | --- | --- |
| Adam et al [1] | 2012 | Integrating a quantitative risk appraisal in a health impact assessment: analysis of the novel smoke-free policy in Hungary | Hungary | Independent BoD study |
| Agardh et al [2] | 2011 | Burden of type 2 diabetes attributed to lower educational levels in Sweden | Sweden | Independent BoD study |
| Agardh et al [3] | 2016 | Alcohol-attributed disease burden in four Nordic countries: a comparison using the Global Burden of Disease, Injuries and Risk Factors 2013 study | Denmark, Finland, Norway, Sweden | GBD-linked study |
| Agardh et al [4] | 2008 | [The contribution of risk factors to the burden of disease in Sweden. A comparison between Swedish and WHO data] | Sweden | GBD-linked study |
| Agardh et al [4] | 2018 | Hälsoutvecklingen i Stockholm bättre än i övriga landet - Jämförelse av hälsoläget utifrån globala sjukdomsbördeprojektet. | Sweden | GBD-linked study |
| Allender and Rayner [5] | 2007 | The burden of overweight and obesity-related ill health in the UK | UK | GBD-linked study |
| Allender et al [6] | 2009 | The burden of smoking-related ill health in the UK | UK | GBD-linked study |
| Allender et al [7] | 2007 | The burden of physical activity-related ill health in the UK | UK | GBD-linked study |
| Babatola [8] | 2018 | Global burden of diseases attributable to air pollution | Global | GBD-linked study |
| Balakrishnan et al [9] | 2009 | The burden of alcohol-related ill health in the United Kingdom | UK | GBD-linked study |
| Begou et al [10] | 2020 | Effects of road traffic noise on the prevalence of cardiovascular diseases: The case of Thessaloniki, Greece | Greece | Independent BoD study |
| Bello et al [11] | 2003 | Trends in mortality and years of life lost related to alcohol in the Canary Islands, Spain [1980-1998] | Spain | Independent BoD study |
| Bennet et al [12] | 2014 | The global burden of ischemic stroke: Findings of the GBD 2010 study | Global | GBD-linked study |
| Borges et al [13] | 2009 | The burden of disease attributable to smoking in Portugal | Portugal | Independent BoD study |
| Bowe et al [14] | 2019 | Estimates of the 2016 global burden of kidney disease attributable to ambient fine particulate matter air pollution | Global | GBD-linked study |
| Butt et al [15] | 2017 | Global and regional trends in particulate air pollution and attributable health burden over the past 50 years | Global | GBD-linked study |
| Carreras et al [16] | 2020 | Burden of disease from breast cancer attributable to smoking and second-hand smoke exposure in Europe | EU-28 | GBD-linked study |
| Cohen et al [17] | 2017 | Estimates and 25-year trends of the global burden of disease attributable to ambient air pollution: an analysis of data from the Global Burden of Diseases Study 2015 | Global | GBD-linked study |
| Collaborators, G. B. D. Risk Factor [18] | 2017 | Global, regional, and national comparative risk assessment of 84 behavioural, environmental and occupational, and metabolic risks or clusters of risks, 1990-2016; a systematic analysis for the Global Burden of Disease Study 2016 | Global | GBD-linked study |
| Collaborators, G. B. D. Risk Factors [19] | 2016 | Global, regional, and national comparative risk assessment of 79 behavioural, environmental and occupational, and metabolic risks or clusters of risks, 1990 2015; a systematic analysis for the Global Burden of Disease Study 2015 | Global | GBD-linked study |
| Cortez-Pinto et al [20] | 2010 | The burden of disease and the cost of illness attributable to alcohol drinking - Results of a national study | Portugal | Independent BoD study |
| De Oliviera et al [21] | 2019 | Estimation of the burden of disease attributable to red meat consumption in France: Influence on colorectal cancer and cardiovascular diseases | France | Independent BoD study |
| Degenhardt et al [22] | 2014 | The global epidemiology and burden of psychostimulant dependence: Findings from the Global Burden of Disease Study 2010 | Global | GBD-linked study |
| Degenhardt et al [23] | 2016 | Estimating the burden of disease attributable to injecting drug use as a risk factor for HIV, hepatitis C, and hepatitis B: findings from the Global Burden of Disease Study 2013 | Global | GBD-linked study |
| Degenhardt et al [24] | 2013 | Global burden of disease attributable to illicit drug use and dependence: Findings from the Global Burden of Disease Study 2010 | Global | GBD-linked study |
| Direção-Geral da Saúde [25] | 2018 | Portugal: The Nation’s Health 1990–2016 | Portugal | GBD-linked study |
| Driscoll et al [26] | 2020 | Global and regional burden of chronic respiratory disease in 2016 arising from non-infectious airborne occupational exposures: A systematic analysis for the Global Burden of Disease Study 2016 | Global | GBD-linked study |
| Driscoll et al [27] | 2005 | The global burden of-disease due to occupational carcinogens | Global | Independent BoD study |
| Dzhambov et al. [28] | 2015 | Road traffic noise and annoyance: exposure-response relationship and burden of disease calculations in Bulgaria | Bulgaria | Independent BoD study |
| Effertz et al [29] | 2013 | The burden and cost of disorders of the brain in Europe with the inclusion of harmful alcohol use and nicotine addiction | Germany | Independent BoD study |
| Ezzati et al [30] | 2002 | Comparative Risk Assessment Collaborating Group: Selected major risk factors and global and regional burden of disease. | Global | GBD-linked study |
| Feigin et al [31] | 2019 | Global, regional, and national burden of neurological disorders, 1990–2016: a systematic analysis for the Global Burden of Disease Study 2016 | Global | GBD-linked study |
| Ferrari et al [32] | 2013 | Burden of depressive disorders by country, sex, age, and year: findings from the global burden of disease study 2010. | Global | GBD-linked study |
| Ferrari et al [33] | 2014 | The burden attributable to mental and substance use disorders as risk factors for suicide: Findings from the Global Burden of Disease Study 2010 | Global | GBD-linked study |
| GBD 2013 Risk Factors Collaborators (Forouzanfar, M H et al) [34] | 2015 | Global, regional, and national comparative risk assessment of 79 behavioural, environmental and occupational, and metabolic risks or clusters of risks in 188 countries, 1990-2013: A systematic analysis for the Global Burden of Disease Study 2013 | Global | GBD-linked study |
| GBD 2016 Alcohol and Drug Use Collaborators [35] | 2018 | The global burden of disease attributable to alcohol and drug use in 195 countries and territories, 1990–2016: a systematic analysis for the Global Burden of Disease Study 2016 | Global | GBD-linked study |
| GBD 2016 Occupational Carcinogens Collaborators [36] | 2020 | Global and regional burden of cancer in 2016 arising from occupational exposure to selected carcinogens: a systematic analysis for the Global Burden of Disease Study 2016 | Global | GBD-linked study |
| GBD 2017 collaborators[37] | 2018 | Global, regional, and national incidence, prevalence, and years lived with disability for 354 diseases and injuries for 195 countries and territories, 1990–2017: a systematic analysis for the Global Burden of Disease Study 2017 | Global | GBD-linked study |
| Gouveia et al [38] | 2004 | Burden of disease from hypercholesterolemia in Portugal | Portugal | Independent BoD study |
| Grandjean et al [39] | 2017 | Calculation of the disease burden associated with environmental chemical exposures: Application of toxicological information in health economic estimation | Global | GBD-linked study |
| Guillois-Becel et al [40] | 2007 | Quantification of years of life lost attributable to chronic air pollution exposure in a health impact assessment: The case of Nantes | France, Nantes city | Independent BoD study |
| Hänninen et al [41] | 2014 | Environmental burden of disease in Europe: assessing nine risk factors in six countries | Belgium, Finland, France, Germany, Italy, and the Netherlands | GBD-linked study |
| Holnicki et al [42] | 2017 | Burden of mortality and disease attributable to multiple air pollutants in Warsaw, Poland | Poland | Independent BoD study |
| Jakobsen et al [43] | 2016 | Burden of disease of dietary exposure to acrylamide in Denmark | Denmark | Independent BoD study |
| Jarosinska et al [44] | 2014 | Towards estimating the burden of disease attributable to second-hand smoke exposure in Polish children | Poland | Independent BoD study |
| Kassomenos et al [45] | 2013 | Human health damage caused by particulate matter PM10 and ozone in urban environments: the case of Athens, Greece | Greece | Independent BoD study |
| Keall et al [46] | 2011 | Injuries associated with housing conditions in Europe: a burden of disease study based on 2004 injury data | Global | Independent BoD study |
| Kellerborg et al [47] | 2016 | Disease burden attributed to alcohol: How methodological advances in the Global Burden of Disease 2013 study have changed the estimates in Sweden | Sweden | GBD-linked study |
| Knol et al [48] | 2005 | Trends in the environmental burden of disease in the Netherlands 1980 – 2020 | The Netherlands | Independent BoD study |
| Knudsen et al [49] | 2019 | Life expectancy and disease burden in the Nordic countries: results from the Global Burden of Diseases, Injuries, and Risk Factors Study 2017. | Nordic countries (Denmark, Finland, Iceland, Norway, Sweden) and Greenland | GBD-linked study |
| Knudsen et al [50] | 2016 | Sykdomsbyrde i Norge 1990-2013 | Norway | GBD-linked study |
| Knudsen et al [51] | 2017 | Sykdomsbyrde i Norge 2015 | Norway | GBD-linked study |
| Lai et al [52] | 2004 | Haiguskoormuse tõttu kaotatud eluaastad Eestis: seosed riskifaktoritega ja riskide vähendamise kulutõhusus | Estonia | Independent BoD study |
| Lawes et al [53] | 2006 | Blood pressure and the global burden of disease 2000. Part II: estimates of attributable burden | Global | GBD-linked study |
| Lehtomäki et al [54] | 2018 | Health impacts of ambient air pollution in Finland | Finland | Independent BoD study |
| Lelieveld et al [55] | 2019 | Cardiovascular disease burden from ambient air pollution in Europe reassessed using novel hazard ratio functions | Europe | Independent BoD study |
| Li et al [56] | 2020 | Trends and risk factors of mortality and disability adjusted life years for chronic respiratory diseases from 1990 to 2017: systematic analysis for the Global Burden of Disease Study 2017 | Global | GBD-linked study |
| Lim et al [57] | 2012 | A comparative risk assessment of burden of disease and injury attributable to 67 risk factors and risk factor clusters in 21 regions, 1990-2010: A systematic analysis for the Global Burden of Disease Study 2010 | Global | GBD-linked study |
| Lock et al [58] | 2005 | The global burden of disease attributable to low consumption of fruit and vegetables: Implications for gobal strategy on diet | Global | Independent BoD study |
| Lopez et al [59] | 2006 | Global and regional burden of disease and risk factors, 2001: systematic analysis of population health data | Global | GBD-linked study |
| Marmet et al [60] | 2014 | Alcohol-attributable mortality in Switzerland in 2011 - Age-specific causes of death and impact of heavy versus non-heavy drinking | Switzerland | Independent BoD study |
| Martín-Ramiro et al [61] | 2014 | Disability attributable to excess weight in Spain | Spain | Independent BoD study |
| May et al [62] | 2015 | The impact of a healthy lifestyle on Disability-Adjusted Life Years: a prospective cohort study. | The Netherlands | Independent BoD study |
| Meier et al [63] | 2017 | Global Burden of Sugar-Related Dental Diseases in 168 Countries and Corresponding Health Care Costs | Global | GBD-linked study |
| Meier et al [64] | 2019 | Cardiovascular mortality attributable to dietary risk factors in 51 countries in the WHO European Region from 1990 to 2016: a systematic analysis of the Global Burden of Disease Study | WHO European region | GBD-linked study |
| Meijerink et al [65] | 2017 | Modelling the burden of hepatitis C infection among people who inject drugs in Norway | Norway | Independent BoD study |
| Miazgowski et al [66] | 2018 | Deaths, disability-adjusted life years and years of life lost due to elevated systolic blood pressure in Poland: Estimates for the global burden of disease study 2016 | Poland | GBD-linked study |
| Mokdad et al [67] | 2016 | Global burden of diseases, injuries, and risk factors for young people's health during 1990-2013: a systematic analysis for the Global Burden of Disease Study 2013 | Global | GBD-linked study |
| Möller et al [68] | 2012 | Health planning for the future: Comparative risk assessment of five major lifestyle risk factors: Evidence from the Wirral, UK | UK | Independent BoD study |
| Monasta et al [69] | 2019 | Italy's health performance, 1990-2017: findings from the Global Burden of Disease Study 2017 | Italy | GBD-linked study |
| Murphy et al [70] | 2018 | Ischaemic heart disease in the former Soviet Union 1990–2015 according to the Global Burden of Disease 2015 Study | Global | GBD-linked study |
| Murray et al [71] | 2013 | UK health performance: findings of the Global Burden of Disease Study 2010 | UK | GBD-linked study |
| Newton et al [72] | 2015 | Changes in health in England, with analysis by English regions and areas of deprivation, 1990–2013: a systematic analysis for the Global Burden of Disease Study 2013 | England | GBD-linked study |
| Oberg et al [73] | 2011 | Worldwide burden of disease from exposure to second-hand smoke: a retrospective analysis of data from 192 countries | Global | Independent BoD study |
| Öberg et al [74] | 2010 | Global estimate of the burden of disease from second-hand smoke | Global | Independent BoD study |
| Oberoi et al [75] | 2019 | Global burden of cancer and coronary heart disease resulting from dietary exposure to arsenic, 2015 | Global | Independent BoD study |
| Orru et al [76] | 2009 | Health impact assessment of particulate pollution in Tallinn using fine spatial resolution and modeling techniques | Estonia | Independent BoD study |
| Orru et al | 2011 | Välisõhu kvaliteedi mõju inimeste tervisele – peentest osakestest tuleneva mõju hindamine kogu Eesti lõikes | Estonia | Independent BoD study |
| Øverland et al [77] | 2018 | Sykdomsbyrden i Norge 2016 | Norway | GBD-linked study |
| Papadimitriou et al [78] | 2017 | Burden of hip fracture using disability-adjusted life-years: a pooled analysis of prospective cohorts in the CHANCES consortium | Europe, USA | Independent BoD study |
| Paunovic et al [79] | 2014 | Burden of myocardial infarction attributable to road-traffic noise: a pilot study in Belgrade | Belgrade, Serbia | Independent BoD study |
| Pomerleau et al [80] | 2006 | The burden of cardiovascular disease and cancer attributable to low fruit and vegetable intake in the European Union: Differences between old and new Member States | EU-15 and EU-10 | Independent BoD study |
| Powles et al [81] | 2005 | The contribution of leading diseases and risk factors to excess losses of healthy life in eastern Europe: Burden of disease study | Three epidemiologically-defined subregions of the WHO region for Europe | GBD-linked study |
| Rayner et al [82] | 2005 | The burden of food related ill health in the UK | Global | GBD-linked study |
| Rehm et al [83] | 2012 | Alcohol consumption, alcohol dependence and attributable burden of disease in Europe: Potential gains from effective interventions for alcohol dependence | Europe Union countries, plus Iceland, Norway, and Switzerland, plus Russia as an external comparison | GBD-linked study |
| Rehm et al [84] | 2007 | Alcohol consumption and alcohol-attributable burden of disease in Switzerland, 2002 | Switzerland | Independent BoD study |
| Rehm et al [85] | 2019 | Trends in substance use and in the attributable burden of disease and mortality in the WHO European Region, 2010-16 | Europe | GBD-linked study |
| Rehm et al [86] | 2009 | Global burden of disease and injury and economic cost attributable to alcohol use and alcohol-use disorders | Global | GBD-linked study |
| Reitsma et al [87] | 2017 | Smoking prevalence and attributable disease burden in 195 countries and territories, 1990-2015: A systematic analysis from the global burden of disease study 2015 | Global | GBD-linked study |
| Rovira et al [88] | 2020 | Air quality, health impacts and burden of disease due to air pollution (PM10, PM2.5, NO2 and O3): Application of AirQ+ model to the Camp de Tarragona County (Catalonia, Spain) | Catalonia, Spain | Independent BoD study |
| Safiri et al [89] | 2019 | The global, regional, and national and its attributable risk factors in 195 countries and territories, 1990-2017: a systematic analysis for the Global Burden of Disease Study 2017 | Global | GBD-linked study |
| Savolahti et al [90] | 2019 | Residential wood combustion in Finland: PM2.5 emissions and health impacts with and without abatement measures | Finland | Independent BoD study |
| Schwingshackl et al [91] | 2019 | Intake of 12 food groups and disability-adjusted life years from coronary heart disease, stroke, type 2 diabetes, and colorectal cancer in 16 European countries | Europe | Independent BoD study |
| Shield et al [92] | 2013 | Alcohol consumption, alcohol dependence, and related mortality in Italy in 2004: effects of treatment-based interventions on alcohol dependence. | Italy | Independent BoD study |
| Shield et al [93] | 2015 | Russia-specific relative risks and their effects on the estimated alcohol-attributable burden of disease | Russia | Independent BoD study |
| Shield et al [94] | 2020 | National, regional, and global burdens of disease from 2000 to 2016 attributable to alcohol use: a comparative risk assessment study | Global | GBD-linked study |
| Shield et al [95] | 2012 | Global burden of injuries attributable to alcohol consumption in 2004: a novel way of calculating the burden of injuries attributable to alcohol consumption | Global | GBD-linked study |
| Siddiqi et al [96] | 2015 | Global burden of disease due to smokeless tobacco consumption in adults: analysis of data from 113 countries. | Global | Independent BoD study |
| Sifaki-Pistolla et al [97] | 2017 | Lung cancer and tobacco smoking in Crete, Greece: reflections from a population-based cancer registry from 1992 to 2013 | Crete, Greece | Independent BoD study |
| Šipetić et al [98] | 2013 | The burden of disease preventable by risk factor reduction in Serbia | Serbia without Kosovo and Metohia | Independent BoD study |
| Stanaway et al [99] | 2018 | Global, regional, and national comparative risk assessment of 84 behavioural, environmental and occupational, and metabolic risks or clusters of risks for 195 countries and territories, 1990-2017: A systematic analysis for the Global Burden of Disease Study 2017 | Global | GBD-linked study |
| Steel et al [100] | 2018 | Changes in health in the countries of the UK and 150 English Local Authority areas 1990–2016: a systematic analysis for the Global Burden of Disease Study 2016 | UK | GBD-linked study |
| Tobollik et al [101] | 2019 | Burden of Disease Due to Traffic Noise in Germany | Germany | Independent BoD study |
| Tod et al [102] | 2018 | Hospital admissions, deaths and overall burden of disease attributable to alcohol consumption in Scotland | Scotland | Independent BoD study |
| Tod et al [103] | 2019 | What causes the burden of stroke in Scotland? A comparative risk assessment approach linking the Scottish Health Survey to administrative health data | Scotland | Independent BoD study |
| Troeger et al [104] | 2018 | Estimates of the global, regional, and national morbidity, mortality, and aetiologies of lower respiratory infections in 195 countries, 1990-2016: a systematic analysis for the Global Burden of Disease Study 2016 | Global | GBD-linked study |
| Tsilidis et al [105] | 2016 | Burden of Cancer in a Large Consortium of Prospective Cohorts in Europe | Denmark, Greece, The Netherlands, Spain, Sweden, Germany, Norway and UK | Independent BoD study |
| Tyrovolas et al [106] | 2018 | The burden of disease in Greece, health loss, risk factors, and health financing, 2000-16: an analysis of the Global Burden of Disease Study 2016 | Greece | GBD-linked study |
| Valent et al [107] | 2004 | Burden of disease attributable to selected environmental factors and injury among children and adolescents in Europe | 51 countries WHO European region | Independent BoD study |
| Van Kreijl et al [108] | 2006 | Our food, our health-Healthy diet and safe food in the Netherlands | The Netherlands | Independent BoD study |
| Vienneau et al [109] | 2015 | Years of life lost and morbidity cases attributable to transportation noise and air pollution: A comparative health risk assessment for Switzerland in 2010 | Switzerland | Independent BoD study |
| Whiteford et al [110] | 2013 | Global burden of disease attributable to mental and substance use disorders: Findings from the Global Burden of Disease Study 2010 | Global | GBD-linked study |
| WHO [111] | 2008 | The Global Burden of Disease: 2004 Update. | WHO region | GBD-linked study |

[1] B. Ádám, Á. Molnár, G. Gulis, and R. Ádány, “Integrating a quantitative risk appraisal in a health impact assessment: analysis of the novel smoke-free policy in Hungary,” *Eur. J. Public Health*, vol. 23, no. 2, pp. 211–217, Apr. 2013, doi: 10.1093/eurpub/cks018.

[2] E. E. Agardh *et al.*, “Burden of type 2 diabetes attributed to lower educational levels in Sweden,” *Popul. Health Metr.*, vol. 9, p. 60, Dec. 2011, doi: 10.1186/1478-7954-9-60.

[3] E. E. Agardh *et al.*, “Alcohol-attributed disease burden in four Nordic countries: a comparison using the Global Burden of Disease, Injuries and Risk Factors 2013 study,” *Addict. Abingdon Engl.*, vol. 111, no. 10, pp. 1806–1813, Oct. 2016, doi: 10.1111/add.13430.

[4] E. Agardh, T. Moradi, and P. Allebeck, “[The contribution of risk factors to the burden of disease in Sweden. A comparison between Swedish and WHO data],” *Lakartidningen*, vol. 105, no. 11, pp. 816–821, Mar. 2008.

[5] S. Allender and M. Rayner, “The burden of overweight and obesity-related ill health in the UK,” *Obes. Rev. Off. J. Int. Assoc. Study Obes.*, vol. 8, no. 5, pp. 467–473, Sep. 2007, doi: 10.1111/j.1467-789X.2007.00394.x.

[6] S. Allender, R. Balakrishnan, P. Scarborough, P. Webster, and M. Rayner, “The burden of smoking-related ill health in the UK,” *Tob. Control*, vol. 18, no. 4, pp. 262–267, Aug. 2009, doi: 10.1136/tc.2008.026294.

[7] S. Allender, C. Foster, P. Scarborough, and M. Rayner, “The burden of physical activity-related ill health in the UK,” *J. Epidemiol. Community Health*, vol. 61, no. 4, pp. 344–348, Apr. 2007, doi: 10.1136/jech.2006.050807.

[8] S. S. Babatola, “Global burden of diseases attributable to air pollution,” *J. Public Health Afr.*, vol. 9, no. 3, p. 813, Dec. 2018, doi: 10.4081/jphia.2018.813.

[9] R. Balakrishnan, S. Allender, P. Scarborough, P. Webster, and M. Rayner, “The burden of alcohol-related ill health in the United Kingdom,” *J. Public Health Oxf. Engl.*, vol. 31, no. 3, pp. 366–373, Sep. 2009, doi: 10.1093/pubmed/fdp051.

[10] P. Begou, P. Kassomenos, and A. Kelessis, “Effects of road traffic noise on the prevalence of cardiovascular diseases: The case of Thessaloniki, Greece,” *Sci. Total Environ.*, vol. 703, p. 134477, Feb. 2020, doi: 10.1016/j.scitotenv.2019.134477.

[11] L. M. Bello, P. Saavedra, and L. Serra, “[Trends in mortality and years of life lost related to alcohol in the Canary Islands, Spain [1980-1998]],” *Gac. Sanit.*, vol. 17, no. 6, pp. 466–473, 2003, doi: 10.1016/s0213-9111(03)71793-2.

[12] D. A. Bennett *et al.*, “The global burden of ischemic stroke: findings of the GBD 2010 study,” *Glob. Heart*, vol. 9, no. 1, pp. 107–112, Mar. 2014, doi: 10.1016/j.gheart.2014.01.001.

[13] M. Borges, M. Gouveia, J. Costa, L. Dos Santos Pinheiro, S. Paulo, and A. Vaz Carneiro, “The burden of disease attributable to smoking in Portugal,” *Rev. Port. Pneumol.*, vol. 15, no. 6, pp. 951–1004, 2009.

[14] B. Bowe, Y. Xie, T. Li, Y. Yan, H. Xian, and Z. Al-Aly, “Estimates of the 2016 global burden of kidney disease attributable to ambient fine particulate matter air pollution,” *BMJ Open*, vol. 9, no. 5, p. e022450, May 2019, doi: 10.1136/bmjopen-2018-022450.

[15] E. W. Butt *et al.*, “Global and regional trends in particulate air pollution and attributable health burden over the past 50 years,” *Environ. Res. Lett.*, vol. 12, no. 10, p. 104017, Oct. 2017, doi: 10.1088/1748-9326/aa87be.

[16] G. Carreras *et al.*, “Burden of disease from breast cancer attributable to smoking and second-hand smoke exposure in Europe,” *Int. J. Cancer*, vol. 147, no. 9, pp. 2387–2393, Nov. 2020, doi: 10.1002/ijc.33021.

[17] A. J. Cohen *et al.*, “Estimates and 25-year trends of the global burden of disease attributable to ambient air pollution: an analysis of data from the Global Burden of Diseases Study 2015,” *Lancet Lond. Engl.*, vol. 389, no. 10082, pp. 1907–1918, May 2017, doi: 10.1016/S0140-6736(17)30505-6.

[18] GBD 2016 Risk Factors Collaborators, “Global, regional, and national comparative risk assessment of 84 behavioural, environmental and occupational, and metabolic risks or clusters of risks, 1990-2016: a systematic analysis for the Global Burden of Disease Study 2016,” *Lancet Lond. Engl.*, vol. 390, no. 10100, pp. 1345–1422, Sep. 2017, doi: 10.1016/S0140-6736(17)32366-8.

[19] GBD 2015 Risk Factors Collaborators, “Global, regional, and national comparative risk assessment of 79 behavioural, environmental and occupational, and metabolic risks or clusters of risks, 1990-2015: a systematic analysis for the Global Burden of Disease Study 2015,” *Lancet Lond. Engl.*, vol. 388, no. 10053, pp. 1659–1724, Oct. 2016, doi: 10.1016/S0140-6736(16)31679-8.

[20] H. Cortez-Pinto, M. Gouveia, L. dos Santos Pinheiro, J. Costa, M. Borges, and A. Vaz Carneiro, “The burden of disease and the cost of illness attributable to alcohol drinking--results of a national study,” *Alcohol. Clin. Exp. Res.*, vol. 34, no. 8, pp. 1442–1449, Aug. 2010, doi: 10.1111/j.1530-0277.2010.01229.x.

[21] J. De Oliveira Mota, G. Boué, S. Guillou, F. Pierre, and J.-M. Membré, “Estimation of the burden of disease attributable to red meat consumption in France: Influence on colorectal cancer and cardiovascular diseases,” *Food Chem. Toxicol. Int. J. Publ. Br. Ind. Biol. Res. Assoc.*, vol. 130, pp. 174–186, Aug. 2019, doi: 10.1016/j.fct.2019.05.023.

[22] L. Degenhardt *et al.*, “The global epidemiology and burden of psychostimulant dependence: findings from the Global Burden of Disease Study 2010,” *Drug Alcohol Depend.*, vol. 137, pp. 36–47, Apr. 2014, doi: 10.1016/j.drugalcdep.2013.12.025.

[23] L. Degenhardt *et al.*, “Estimating the burden of disease attributable to injecting drug use as a risk factor for HIV, hepatitis C, and hepatitis B: findings from the Global Burden of Disease Study 2013,” *Lancet Infect. Dis.*, vol. 16, no. 12, pp. 1385–1398, Dec. 2016, doi: 10.1016/S1473-3099(16)30325-5.

[24] L. Degenhardt *et al.*, “Global burden of disease attributable to illicit drug use and dependence: findings from the Global Burden of Disease Study 2010,” *Lancet Lond. Engl.*, vol. 382, no. 9904, pp. 1564–1574, Nov. 2013, doi: 10.1016/S0140-6736(13)61530-5.

[25] Direção-Geral da Saúde, Institute for Health Metrics and Evaluation., “Portugal: The Nation’s Health 1990–2016: An overview of the Global Burden of Disease Study 2016 Results.,” *Seattle, WA: IHME, 2018*, Apr. 04, 2018. https://www.healthdata.org/policy-report/portugal-nation%E2%80%99s-health-1990%E2%80%932016 (accessed Feb. 05, 2023).

[26] GBD 2016 Occupational Chronic Respiratory Risk Factors Collaborators and GBD 2016 occupational chronic respiratory risk factors collaborators, “Global and regional burden of chronic respiratory disease in 2016 arising from non-infectious airborne occupational exposures: a systematic analysis for the Global Burden of Disease Study 2016,” *Occup. Environ. Med.*, vol. 77, no. 3, pp. 142–150, Mar. 2020, doi: 10.1136/oemed-2019-106013.

[27] T. Driscoll *et al.*, “The global burden of disease due to occupational carcinogens,” *Am. J. Ind. Med.*, vol. 48, no. 6, pp. 419–431, Dec. 2005, doi: 10.1002/ajim.20209.

[28] A. Dzhambov and D. Dimitrova, “Road traffic noise and annoyance: exposure-response relationship and burden of disease calculations in Bulgaria,” *Scr. Sci. Medica*, vol. 47, no. 2, p. 22, Jun. 2015, doi: 10.14748/ssm.v47i2.1153.

[29] T. Effertz and K. Mann, “The burden and cost of disorders of the brain in Europe with the inclusion of harmful alcohol use and nicotine addiction,” *Eur. Neuropsychopharmacol. J. Eur. Coll. Neuropsychopharmacol.*, vol. 23, no. 7, pp. 742–748, Jul. 2013, doi: 10.1016/j.euroneuro.2012.07.010.

[30] M. Ezzati, A. D. Lopez, A. Rodgers, S. Vander Hoorn, C. J. L. Murray, and Comparative Risk Assessment Collaborating Group, “Selected major risk factors and global and regional burden of disease,” *Lancet Lond. Engl.*, vol. 360, no. 9343, pp. 1347–1360, Nov. 2002, doi: 10.1016/S0140-6736(02)11403-6.

[31] GBD 2015 Neurological Disorders Collaborator Group, “Global, regional, and national burden of neurological disorders during 1990-2015: a systematic analysis for the Global Burden of Disease Study 2015,” *Lancet Neurol.*, vol. 16, no. 11, pp. 877–897, Nov. 2017, doi: 10.1016/S1474-4422(17)30299-5.

[32] A. J. Ferrari *et al.*, “Burden of depressive disorders by country, sex, age, and year: findings from the global burden of disease study 2010,” *PLoS Med.*, vol. 10, no. 11, p. e1001547, Nov. 2013, doi: 10.1371/journal.pmed.1001547.

[33] A. J. Ferrari *et al.*, “The burden attributable to mental and substance use disorders as risk factors for suicide: findings from the Global Burden of Disease Study 2010,” *PloS One*, vol. 9, no. 4, p. e91936, 2014, doi: 10.1371/journal.pone.0091936.

[34] GBD 2013 Risk Factors Collaborators *et al.*, “Global, regional, and national comparative risk assessment of 79 behavioural, environmental and occupational, and metabolic risks or clusters of risks in 188 countries, 1990-2013: a systematic analysis for the Global Burden of Disease Study 2013,” *Lancet Lond. Engl.*, vol. 386, no. 10010, pp. 2287–2323, Dec. 2015, doi: 10.1016/S0140-6736(15)00128-2.

[35] GBD 2016 Alcohol and Drug Use Collaborators, “The global burden of disease attributable to alcohol and drug use in 195 countries and territories, 1990-2016: a systematic analysis for the Global Burden of Disease Study 2016,” *Lancet Psychiatry*, vol. 5, no. 12, pp. 987–1012, Dec. 2018, doi: 10.1016/S2215-0366(18)30337-7.

[36] GBD 2016 Occupational Carcinogens Collaborators, “Global and regional burden of cancer in 2016 arising from occupational exposure to selected carcinogens: a systematic analysis for the Global Burden of Disease Study 2016,” *Occup. Environ. Med.*, vol. 77, no. 3, pp. 151–159, Mar. 2020, doi: 10.1136/oemed-2019-106012.

[37] GBD 2017 Disease and Injury Incidence and Prevalence Collaborators, “Global, regional, and national incidence, prevalence, and years lived with disability for 354 diseases and injuries for 195 countries and territories, 1990-2017: a systematic analysis for the Global Burden of Disease Study 2017,” *Lancet Lond. Engl.*, vol. 392, no. 10159, pp. 1789–1858, Nov. 2018, doi: 10.1016/S0140-6736(18)32279-7.

[38] M. Gouveia, M. Borges, J. Costa, and A. V. Carneiro, “Burden of disease from hypercholesterolemia in Portugal,” *Rev. Port. Cardiol. Orgao Of. Soc. Port. Cardiol. Port. J. Cardiol. Off. J. Port. Soc. Cardiol.*, vol. 23, no. 2, pp. 255–270, Feb. 2004.

[39] P. Grandjean and M. Bellanger, “Calculation of the disease burden associated with environmental chemical exposures: application of toxicological information in health economic estimation,” *Environ. Health Glob. Access Sci. Source*, vol. 16, no. 1, p. 123, Dec. 2017, doi: 10.1186/s12940-017-0340-3.

[40] Y. Guillois-Becel, D. Eilstein, Ph. Glorennec, and A. Lefranc, “Quantification of years of life lost attributable to chronic air pollution exposure in a health impact assessment: the case of Nantes,” *Environ. Risques Sante*, vol. 6, no. 3, pp. 189–197, 2007.

[41] O. Hänninen *et al.*, “Environmental burden of disease in Europe: assessing nine risk factors in six countries,” *Environ. Health Perspect.*, vol. 122, no. 5, pp. 439–446, May 2014, doi: 10.1289/ehp.1206154.

[42] P. Holnicki, M. Tainio, A. Kałuszko, and Z. Nahorski, “Burden of Mortality and Disease Attributable to Multiple Air Pollutants in Warsaw, Poland,” *Int. J. Environ. Res. Public. Health*, vol. 14, no. 11, p. E1359, Nov. 2017, doi: 10.3390/ijerph14111359.

[43] L. S. Jakobsen, K. Granby, V. K. Knudsen, M. Nauta, S. M. Pires, and M. Poulsen, “Burden of disease of dietary exposure to acrylamide in Denmark,” *Food Chem. Toxicol. Int. J. Publ. Br. Ind. Biol. Res. Assoc.*, vol. 90, pp. 151–159, Apr. 2016, doi: 10.1016/j.fct.2016.01.021.

[44] D. Jarosińska, K. Polańska, B. Wojtyniak, and W. Hanke, “Towards estimating the burden of disease attributable to second-hand smoke exposure in Polish children,” *Int. J. Occup. Med. Environ. Health*, vol. 27, no. 1, pp. 38–49, Jan. 2014, doi: 10.2478/s13382-014-0223-6.

[45] P. A. Kassomenos, K. Dimitriou, and A. K. Paschalidou, “Human health damage caused by particulate matter PM10 and ozone in urban environments: the case of Athens, Greece,” *Environ. Monit. Assess.*, vol. 185, no. 8, pp. 6933–6942, Aug. 2013, doi: 10.1007/s10661-013-3076-8.

[46] M. D. Keall, D. Ormandy, and M. G. Baker, “Injuries associated with housing conditions in Europe: a burden of disease study based on 2004 injury data,” *Environ. Health Glob. Access Sci. Source*, vol. 10, p. 98, Nov. 2011, doi: 10.1186/1476-069X-10-98.

[47] K. Kellerborg, A.-K. Danielsson, P. Allebeck, M. M. Coates, and E. Agardh, “Disease burden attributed to alcohol: How methodological advances in the Global Burden of Disease 2013 study have changed the estimates in Sweden,” *Scand. J. Public Health*, vol. 44, no. 6, pp. 604–610, Aug. 2016, doi: 10.1177/1403494816653512.

[48] AB Knol and BAM Staatsen, “Trends in the environmental burden of disease in the Netherlands 1980 – 2020,” 500029001/2005. [Online]. Available: https://www.rivm.nl/bibliotheek/rapporten/500029001.pdf

[49] Nordic Burden of Disease Collaborators, “Life expectancy and disease burden in the Nordic countries: results from the Global Burden of Diseases, Injuries, and Risk Factors Study 2017,” *Lancet Public Health*, vol. 4, no. 12, pp. e658–e669, Dec. 2019, doi: 10.1016/S2468-2667(19)30224-5.

[50] Ann Kristin Knudsen, Jonas Minet Kinge, Vegard Skirbekk, and Stein Emil Vollset, “Sykdomsbyrde i Norge 1990–2013,” Bergen/Oslo: Folkehelseinstituttet, 2016, 2016:1. [Online]. Available: fhi.no/publ/2016/sykdomsbyrde-i-norge-1990-2013/#:~:text=Til%20tross%20for%20at%20befolkningen,leveår%20som%20følge%20av%20hjertesykdom.

[51] Knudsen AK, Tollånes MC, Haaland ØA, Kinge JM, Skirbekk V, Vollset SE, “Sykdomsbyrde i Norge 2015. Resultater fra Global Burden of Diseases, Injuries, and Risk Factors Study 2015 (GBD 2015),” Bergen/Oslo: Folkehelseinstituttet, 2017., Rapport 2017. [Online]. Available: https://www.fhi.no/publ/2017/sykdomsbyrde-i-norge-2015/#:~:text=Forventet%20levealder%20ved%20f%C3%B8dsel%20i,%2Dd%C3%B8delig%20helsetap%20(sykelighet).

[52] T. R. Ülikool, “Haiguskoormuse tõttu kaotatud eluaastad Eestis: seosed riskifaktoritega ja riskide vähendamise kulutõhusus,” 2004.

[53] C. M. M. Lawes, S. Vander Hoorn, M. R. Law, P. Elliott, S. MacMahon, and A. Rodgers, “Blood pressure and the global burden of disease 2000. Part II: estimates of attributable burden,” *J. Hypertens.*, vol. 24, no. 3, pp. 423–430, Mar. 2006, doi: 10.1097/01.hjh.0000209973.67746.f0.

[54] H. Lehtomäki *et al.*, “Health Impacts of Ambient Air Pollution in Finland,” *Int. J. Environ. Res. Public. Health*, vol. 15, no. 4, p. 736, Apr. 2018, doi: 10.3390/ijerph15040736.

[55] J. Lelieveld *et al.*, “Cardiovascular disease burden from ambient air pollution in Europe reassessed using novel hazard ratio functions,” *Eur. Heart J.*, vol. 40, no. 20, pp. 1590–1596, May 2019, doi: 10.1093/eurheartj/ehz135.

[56] X. Li, X. Cao, M. Guo, M. Xie, and X. Liu, “Trends and risk factors of mortality and disability adjusted life years for chronic respiratory diseases from 1990 to 2017: systematic analysis for the Global Burden of Disease Study 2017,” *BMJ*, vol. 368, p. m234, Feb. 2020, doi: 10.1136/bmj.m234.

[57] S. S. Lim *et al.*, “A comparative risk assessment of burden of disease and injury attributable to 67 risk factors and risk factor clusters in 21 regions, 1990-2010: a systematic analysis for the Global Burden of Disease Study 2010,” *Lancet Lond. Engl.*, vol. 380, no. 9859, pp. 2224–2260, Dec. 2012, doi: 10.1016/S0140-6736(12)61766-8.

[58] K. Lock, J. Pomerleau, L. Causer, D. R. Altmann, and M. McKee, “The global burden of disease attributable to low consumption of fruit and vegetables: implications for the global strategy on diet,” *Bull. World Health Organ.*, vol. 83, no. 2, pp. 100–108, Feb. 2005.

[59] A. D. Lopez, C. D. Mathers, M. Ezzati, D. T. Jamison, and C. J. L. Murray, “Global and regional burden of disease and risk factors, 2001: systematic analysis of population health data,” *Lancet Lond. Engl.*, vol. 367, no. 9524, pp. 1747–1757, May 2006, doi: 10.1016/S0140-6736(06)68770-9.

[60] S. Marmet, J. Rehm, and G. Gmel, “The importance of age groups in estimates of alcohol-attributable mortality: impact on trends in Switzerland between 1997 and 2011,” *Addict. Abingdon Engl.*, vol. 111, no. 2, pp. 255–262, Feb. 2016, doi: 10.1111/add.13164.

[61] J. J. Martín-Ramiro, E. Alvarez-Martín, and R. Gil-Prieto, “[Disability attributable to excess weight in Spain],” *Med. Clin. (Barc.)*, vol. 143, no. 4, pp. 150–156, Aug. 2014, doi: 10.1016/j.medcli.2013.05.028.

[62] A. M. May *et al.*, “The impact of a healthy lifestyle on Disability-Adjusted Life Years: a prospective cohort study,” *BMC Med.*, vol. 13, p. 39, Feb. 2015, doi: 10.1186/s12916-015-0287-6.

[63] T. Meier, P. Deumelandt, O. Christen, G. I. Stangl, K. Riedel, and M. Langer, “Global Burden of Sugar-Related Dental Diseases in 168 Countries and Corresponding Health Care Costs,” *J. Dent. Res.*, vol. 96, no. 8, pp. 845–854, Jul. 2017, doi: 10.1177/0022034517708315.

[64] T. Meier *et al.*, “Cardiovascular mortality attributable to dietary risk factors in 51 countries in the WHO European Region from 1990 to 2016: a systematic analysis of the Global Burden of Disease Study,” *Eur. J. Epidemiol.*, vol. 34, no. 1, pp. 37–55, Jan. 2019, doi: 10.1007/s10654-018-0473-x.

[65] H. Meijerink *et al.*, “Modelling the burden of hepatitis C infection among people who inject drugs in Norway, 1973-2030,” *BMC Infect. Dis.*, vol. 17, no. 1, p. 541, Aug. 2017, doi: 10.1186/s12879-017-2631-2.

[66] T. Miazgowski, A. Taszarek, K. Widecka, B. Miazgowski, and K. Homa, “Deaths, disability-adjusted life years and years of life lost due to elevated systolic blood pressure in Poland: estimates for the Global Burden of Disease Study 2016,” *Arter. Hypertens.*, vol. 22, no. 2, pp. 95–103, Jun. 2018, doi: 10.5603/AH.a2018.0005.

[67] A. H. Mokdad *et al.*, “Global burden of diseases, injuries, and risk factors for young people’s health during 1990-2013: a systematic analysis for the Global Burden of Disease Study 2013,” *Lancet Lond. Engl.*, vol. 387, no. 10036, pp. 2383–2401, Jun. 2016, doi: 10.1016/S0140-6736(16)00648-6.

[68] H. Möller, M. Dherani, C. Harwood, T. Kinsella, and D. Pope, “Health planning for the future: comparative risk assessment of five major lifestyle risk factors: evidence from the Wirral, UK,” *J. Public Health Oxf. Engl.*, vol. 34, no. 3, pp. 430–437, Aug. 2012, doi: 10.1093/pubmed/fds005.

[69] GBD 2017 Italy Collaborators, “Italy’s health performance, 1990-2017: findings from the Global Burden of Disease Study 2017,” *Lancet Public Health*, vol. 4, no. 12, pp. e645–e657, Dec. 2019, doi: 10.1016/S2468-2667(19)30189-6.

[70] A. Murphy *et al.*, “Ischaemic heart disease in the former Soviet Union 1990-2015 according to the Global Burden of Disease 2015 Study,” *Heart Br. Card. Soc.*, vol. 104, no. 1, pp. 58–66, Jan. 2018, doi: 10.1136/heartjnl-2016-311142.

[71] C. J. L. Murray *et al.*, “UK health performance: findings of the Global Burden of Disease Study 2010,” *Lancet Lond. Engl.*, vol. 381, no. 9871, pp. 997–1020, Mar. 2013, doi: 10.1016/S0140-6736(13)60355-4.

[72] J. N. Newton *et al.*, “Changes in health in England, with analysis by English regions and areas of deprivation, 1990-2013: a systematic analysis for the Global Burden of Disease Study 2013,” *Lancet Lond. Engl.*, vol. 386, no. 10010, pp. 2257–2274, Dec. 2015, doi: 10.1016/S0140-6736(15)00195-6.

[73] M. Oberg, M. S. Jaakkola, A. Woodward, A. Peruga, and A. Prüss-Ustün, “Worldwide burden of disease from exposure to second-hand smoke: a retrospective analysis of data from 192 countries,” *Lancet Lond. Engl.*, vol. 377, no. 9760, pp. 139–146, Jan. 2011, doi: 10.1016/S0140-6736(10)61388-8.

[74] M. Öberg, M. S. Jaakkola, A. Prüss-Üstün, A. Peruga, A. Woodward, and World Health Organization, “Global estimate of the burden of disease from second-hand smoke / by Mattias Öberg ... [et al],” 2010, [Online]. Available: https://apps.who.int/iris/handle/10665/44426

[75] S. Oberoi, B. Devleesschauwer, H. J. Gibb, and A. Barchowsky, “Global burden of cancer and coronary heart disease resulting from dietary exposure to arsenic, 2015,” *Environ. Res.*, vol. 171, pp. 185–192, Apr. 2019, doi: 10.1016/j.envres.2019.01.025.

[76] H. Orru *et al.*, “Health impact assessment of particulate pollution in Tallinn using fine spatial resolution and modeling techniques,” *Environ. Health Glob. Access Sci. Source*, vol. 8, p. 7, Mar. 2009, doi: 10.1186/1476-069X-8-7.

[77] M. C. Tollånes, A. K. Knudsen, S. E. Vollset, J. M. Kinge, V. Skirbekk, and S. Øverland, “Sykdomsbyrden i Norge i 2016,” *Tidsskr. Den Nor. Legeforening*, 2018, doi: 10.4045/tidsskr.18.0274.

[78] N. Papadimitriou *et al.*, “Burden of hip fracture using disability-adjusted life-years: a pooled analysis of prospective cohorts in the CHANCES consortium,” *Lancet Public Health*, vol. 2, no. 5, pp. e239–e246, May 2017, doi: 10.1016/S2468-2667(17)30046-4.

[79] K. Paunovic and G. Belojević, “Burden of myocardial infarction attributable to road-traffic noise: a pilot study in Belgrade,” *Noise Health*, vol. 16, no. 73, pp. 374–379, Dec. 2014, doi: 10.4103/1463-1741.144415.

[80] J. Pomerleau, K. Lock, and M. McKee, “The burden of cardiovascular disease and cancer attributable to low fruit and vegetable intake in the European Union: differences between old and new Member States,” *Public Health Nutr.*, vol. 9, no. 5, pp. 575–583, Aug. 2006, doi: 10.1079/phn2005910.

[81] J. W. Powles, W. Zatonski, S. Vander Hoorn, and M. Ezzati, “The contribution of leading diseases and risk factors to excess losses of healthy life in Eastern Europe: burden of disease study,” *BMC Public Health*, vol. 5, p. 116, Nov. 2005, doi: 10.1186/1471-2458-5-116.

[82] M. Rayner and P. Scarborough, “The burden of food related ill health in the UK,” *J. Epidemiol. Community Health*, vol. 59, no. 12, pp. 1054–1057, Dec. 2005, doi: 10.1136/jech.2005.036491.

[83] J. Rehm, K. D. Shield, M. X. Rehm, G. Gmel, and U. Frick, “Alcohol consumption, alcohol dependence, and attributable burden of disease in Europe: Potential gains from effective interventions for alcohol dependence,” 2012, doi: 10.5167/UZH-64919.

[84] J. Rehm, B. Taylor, M. Roerecke, and J. Patra, “Alcohol consumption and alcohol-attributable burden of disease in Switzerland, 2002,” *Int. J. Public Health*, vol. 52, no. 6, pp. 383–392, Dec. 2007, doi: 10.1007/s00038-007-7010-0.

[85] J. Rehm, J. Manthey, K. D. Shield, and C. Ferreira-Borges, “Trends in substance use and in the attributable burden of disease and mortality in the WHO European Region, 2010-16,” *Eur. J. Public Health*, vol. 29, no. 4, pp. 723–728, Aug. 2019, doi: 10.1093/eurpub/ckz064.

[86] J. Rehm, C. Mathers, S. Popova, M. Thavorncharoensap, Y. Teerawattananon, and J. Patra, “Global burden of disease and injury and economic cost attributable to alcohol use and alcohol-use disorders,” *Lancet Lond. Engl.*, vol. 373, no. 9682, pp. 2223–2233, Jun. 2009, doi: 10.1016/S0140-6736(09)60746-7.

[87] GBD 2015 Tobacco Collaborators, “Smoking prevalence and attributable disease burden in 195 countries and territories, 1990-2015: a systematic analysis from the Global Burden of Disease Study 2015,” *Lancet Lond. Engl.*, vol. 389, no. 10082, pp. 1885–1906, May 2017, doi: 10.1016/S0140-6736(17)30819-X.

[88] J. Rovira, J. L. Domingo, and M. Schuhmacher, “Air quality, health impacts and burden of disease due to air pollution (PM10, PM2.5, NO2 and O3): Application of AirQ+ model to the Camp de Tarragona County (Catalonia, Spain),” *Sci. Total Environ.*, vol. 703, p. 135538, Feb. 2020, doi: 10.1016/j.scitotenv.2019.135538.

[89] GBD 2017 Colorectal Cancer Collaborators, “The global, regional, and national burden of colorectal cancer and its attributable risk factors in 195 countries and territories, 1990-2017: a systematic analysis for the Global Burden of Disease Study 2017,” *Lancet Gastroenterol. Hepatol.*, vol. 4, no. 12, pp. 913–933, Dec. 2019, doi: 10.1016/S2468-1253(19)30345-0.

[90] M. Savolahti *et al.*, “Residential Wood Combustion in Finland: PM2.5 Emissions and Health Impacts with and without Abatement Measures,” *Int. J. Environ. Res. Public. Health*, vol. 16, no. 16, p. E2920, Aug. 2019, doi: 10.3390/ijerph16162920.

[91] L. Schwingshackl *et al.*, “Intake of 12 food groups and disability-adjusted life years from coronary heart disease, stroke, type 2 diabetes, and colorectal cancer in 16 European countries,” *Eur. J. Epidemiol.*, vol. 34, no. 8, pp. 765–775, Aug. 2019, doi: 10.1007/s10654-019-00523-4.

[92] K. D. Shield, J. Rehm, G. Gmel, M. X. Rehm, and A. Allamani, “Alcohol consumption, alcohol dependence, and related mortality in Italy in 2004: effects of treatment-based interventions on alcohol dependence,” *Subst. Abuse Treat. Prev. Policy*, vol. 8, p. 21, Jun. 2013, doi: 10.1186/1747-597X-8-21.

[93] K. D. Shield and J. Rehm, “Russia-specific relative risks and their effects on the estimated alcohol-attributable burden of disease,” *BMC Public Health*, vol. 15, p. 482, May 2015, doi: 10.1186/s12889-015-1818-y.

[94] K. Shield *et al.*, “National, regional, and global burdens of disease from 2000 to 2016 attributable to alcohol use: a comparative risk assessment study,” *Lancet Public Health*, vol. 5, no. 1, pp. e51–e61, Jan. 2020, doi: 10.1016/S2468-2667(19)30231-2.

[95] K. D. Shield, G. Gmel, J. Patra, and J. Rehm, “Global burden of injuries attributable to alcohol consumption in 2004: a novel way of calculating the burden of injuries attributable to alcohol consumption,” *Popul. Health Metr.*, vol. 10, no. 1, p. 9, May 2012, doi: 10.1186/1478-7954-10-9.

[96] K. Siddiqi *et al.*, “Global burden of disease due to smokeless tobacco consumption in adults: analysis of data from 113 countries,” *BMC Med.*, vol. 13, p. 194, Aug. 2015, doi: 10.1186/s12916-015-0424-2.

[97] D. Sifaki-Pistolla *et al.*, “Lung cancer and tobacco smoking in Crete, Greece: reflections from a population-based cancer registry from 1992 to 2013,” *Tob. Induc. Dis.*, vol. 15, p. 6, 2017, doi: 10.1186/s12971-017-0114-2.

[98] S. Sipetić *et al.*, “The burden of disease preventable by risk factor reduction in Serbia,” *Vojnosanit. Pregl.*, vol. 70, no. 5, pp. 445–451, May 2013, doi: 10.2298/vsp111024049s.

[99] GBD 2017 Disease and Injury Incidence and Prevalence Collaborators, “Global, regional, and national incidence, prevalence, and years lived with disability for 354 diseases and injuries for 195 countries and territories, 1990-2017: a systematic analysis for the Global Burden of Disease Study 2017,” *Lancet Lond. Engl.*, vol. 392, no. 10159, pp. 1789–1858, Nov. 2018, doi: 10.1016/S0140-6736(18)32279-7.

[100] N. Steel *et al.*, “Changes in health in the countries of the UK and 150 English Local Authority areas 1990-2016: a systematic analysis for the Global Burden of Disease Study 2016,” *Lancet Lond. Engl.*, vol. 392, no. 10158, pp. 1647–1661, Nov. 2018, doi: 10.1016/S0140-6736(18)32207-4.

[101] M. Tobollik, M. Hintzsche, J. Wothge, T. Myck, and D. Plass, “Burden of Disease Due to Traffic Noise in Germany,” *Int. J. Environ. Res. Public. Health*, vol. 16, no. 13, p. 2304, Jun. 2019, doi: 10.3390/ijerph16132304.

[102] E. Tod *et al.*, *Hospital admissions, deaths and overall burden of disease attributable to alcohol consumption in Scotland*. 2018.

[103] E. Tod *et al.*, “What causes the burden of stroke in Scotland? A comparative risk assessment approach linking the Scottish Health Survey to administrative health data,” *PloS One*, vol. 14, no. 7, p. e0216350, 2019, doi: 10.1371/journal.pone.0216350.

[104] GBD 2016 Lower Respiratory Infections Collaborators, “Estimates of the global, regional, and national morbidity, mortality, and aetiologies of lower respiratory infections in 195 countries, 1990-2016: a systematic analysis for the Global Burden of Disease Study 2016,” *Lancet Infect. Dis.*, vol. 18, no. 11, pp. 1191–1210, Nov. 2018, doi: 10.1016/S1473-3099(18)30310-4.

[105] K. K. Tsilidis *et al.*, “Burden of Cancer in a Large Consortium of Prospective Cohorts in Europe,” *J. Natl. Cancer Inst.*, vol. 108, no. 10, p. djw127, Oct. 2016, doi: 10.1093/jnci/djw127.

[106] Global Burden of Disease 2016 Greece Collaborators, “The burden of disease in Greece, health loss, risk factors, and health financing, 2000-16: an analysis of the Global Burden of Disease Study 2016,” *Lancet Public Health*, vol. 3, no. 8, pp. e395–e406, Aug. 2018, doi: 10.1016/S2468-2667(18)30130-0.

[107] F. Valent, D. Little, R. Bertollini, L. E. Nemer, F. Barbone, and G. Tamburlini, “Burden of disease attributable to selected environmental factors and injury among children and adolescents in Europe,” *Lancet Lond. Engl.*, vol. 363, no. 9426, pp. 2032–2039, Jun. 2004, doi: 10.1016/S0140-6736(04)16452-0.

[108] K. van, K. AGAC, R. van, and CVG, “Our food, our health-Healthy diet and safe food in the Netherlands,” Jan. 2006.

[109] D. Vienneau *et al.*, “Years of life lost and morbidity cases attributable to transportation noise and air pollution: A comparative health risk assessment for Switzerland in 2010,” *Int. J. Hyg. Environ. Health*, vol. 218, no. 6, pp. 514–521, Aug. 2015, doi: 10.1016/j.ijheh.2015.05.003.

[110] H. A. Whiteford *et al.*, “Global burden of disease attributable to mental and substance use disorders: findings from the Global Burden of Disease Study 2010,” *Lancet Lond. Engl.*, vol. 382, no. 9904, pp. 1575–1586, Nov. 2013, doi: 10.1016/S0140-6736(13)61611-6.

[111] World Health Organization, “The global burden of disease : 2004 update,” World Health Organization, 2008. Accessed: Feb. 05, 2023. [Online]. Available: https://apps.who.int/iris/handle/10665/43942
