# Supplementary material for: Burden of disease attributable to risk factors in European countries: a scoping literature review
Source: Arch Public Health. 2023 Jun 25;81:116. doi: 10.1186/s13690-023-01119-x (PMC10290804; doi:10.1186/s13690-023-01119-x)
Supplement: Supplementary file 6 — Additional file 6. Reference list of the excluded studies, including reason for exclusion. [file 13690_2023_1119_MOESM6_ESM.docx]

**Additional file 6: Reference list of the excluded studies, including reason for exclusion**

| Authors | Year | Title | Reason for exclusion |  | Authors | Year | Title | Reason for exclusion |
| --- | --- | --- | --- | --- | --- | --- | --- | --- |
| 3C study group | 2003 | Vascular factors and risk of dementia: Design of the Three-City Study and baseline characteristics of the study population | Wrong study design |  | Iglesias, C. and Taboada, J. | 2014 | Radon in Galicia | wrong outcome |
| Abaci et al | 2012 | Management of cardiovascular risk factors for primary prevention: evaluation of Turkey results of the EURIKA study | Wrong outcome |  | Imamura et al | 2015 | Consumption of sugar sweetened beverages, and fruit juice and incidence of type 2 diabetes: systematic review, meta-analysis, and estimation of population attributable fraction | wrong outcome |
| Adamson et al | 2004 | Is stroke the most common cause of disability? | Wrong outcome |  | Imamura et al | 2016 | Consumption of sugar sweetened beverages, artificially sweetened beverages, and fruit juice and incidence of type 2 diabetes: systematic review, meta-analysis, and estimation of population attributable fraction | duplicate |
| Adjibadeet al | 2017 | Combined healthy lifestyles and risk of depressive symptoms in the nutrinet-Santé cohort | Duplicate |  | Irigaray et al | 2007 | Lifestyle-related factors and environmental agents causing cancer: an overview | wrong study design |
| Adjibadeet al | 2018 | Prospective association between combined healthy lifestyles and risk of depressive symptoms in the French NutriNet-SantÃ© cohort | Wrong outcome |  | Islami et al | 2011 | A cross-sectional study of cardiovascular disease and associated factors | wrong outcome |
| Adliene et al | 2020 | Occupational radiation exposure of health professionals and cancer risk assessment for Lithuanian nuclear medicine workers | wrong outcome |  | Islami et al | 2014 | A systematic review and meta-analysis of tobacco use and prostate cancer mortality and incidence in prospective cohort studies | wrong outcome |
| Adovasio et al | 2015 | Cancer registries underestimate both the type of disease and also number of cases due to pollution | Wrong outcome |  | Jaakkola, M. S. and Jaakkola, J. J. | 2012 | Assessment of public health impact of work-related asthma | wrong study design |
| Agabito et al | 1999 | The impact of parental smoking on asthma and wheezing | Wrong outcome |  | Jacobi, W. | 1999 | Radiation exposure and attributable cancer risk in former miners of the WISMUT uranium mining company | wrong outcome |
| Agudo et al | 2012 | Impact of cigarette smoking on cancer risk in the European prospective investigation into cancer and nutrition study | Wrong outcome |  | Jarvholm et al | 1993 | Quantitative importance of asbestos as a cause of lung cancer in a Swedish industrial city: A case-referent study | wrong outcome |
| Ahlgren et al | 2004 | Growth patterns and the risk of breast cancer in women | Wrong outcome |  | Jarvholm et al | 2011 | Mortality in Sweden related to occupational exposures | wrong outcome |
| Aigner et al | 2018 | Low diet quality and the risk of stroke mortality: The multiethnic cohort study | Wrong outcome |  | Jedrychowski et al | 1992 | Effect of tobacco smoking on various histological types of lung cancer | wrong study design |
| Aigner et al | 2016 | To what extent do established risk factors explain? A case-control study | Wrong outcome |  | Jha et al | 2006 | Social inequalities in male mortality, and in male mortality from smoking: indirect estimation from national death rates in England and Wales, Poland, and North America | wrong outcome |
| Aigner et al | 2016 | To what extent do established risk factors explain stroke in the young? A case-control study | Wrong outcome |  | Jiménez et al | 2009 | Short-term impact of particulate matter (PM2.5) on daily mortality among the over-75 age group in Madrid (Spain) | wrong outcome |
| Aigner et al | 2017 | Contribution of Established Stroke Risk Factors to the Burden of Stroke in Young Adults | Wrong outcome |  | Jimenez, J. D. and Gil, C. L. | 2015 | Efectos en salud del ruido de tráfico: Más allá de las “molestias”. (Health effects of noise trafic: Beyond 'discomfort') | wrong outcome |
| Ajrouche et al | 2017 | Quantitative Health Risk Assessment of Indoor Radon: A Systematic Review | Wrong outcome |  | Jöckel et al | 1997 | Occupationally-induced lung cancer--a quantitative evaluation for the North Germany area | wrong outcome |
| Ajrouche et al | 2018 | Quantitative health impact of indoor radon in France | wrong outcome |  | John, U. and Hanke, M. | 2001 | Mortality attributable to tobacco smoking in german federal states | wrong outcome |
| Akdas et ak | 1990 | Epidemiological case-control study on the etiology of bladder cancer in Turkey | Wrong study design |  | John, U. and Hanke, M. | 2002 | Alcohol attributable mortality in a high per capita consumption country - Germany | wrong outcome |
| Akerkar et al | 2017 | Educational inequalities in mortality of patients with atrial fibrillation in Norway | Wrong outcome |  | John, U. and Hanke, M. | 2002 | Tobacco smoking- and alcohol drinking-attributable cancer mortality in Germany | wrong outcome |
| Akesson et al | 2014 | Low-risk diet and lifestyle habits in the primary prevention of myocardial infarction in men: a population-based prospective cohort study | Wrong study design |  | John, U. and Hanke, M. | 2003 | Tobacco- and alcohol-attributable mortality and years of potential life lost in Germany | wrong outcome |
| Al Tunaiji et al | 2014 | Population attributable fraction of type 2 diabetes due to physical inactivity in adults: a systematic review | Wrong outcome |  | Johnson et al | 2005 | The impact of the 2003 heat wave on daily mortality in England and Wales and the use of rapid weekly mortality estimates | wrong outcome |
| Alavi et al | 2018 | The contribution of alcohol use disorder to decompensated cirrhosis among people with hepatitis C: An international study | Wrong outcome |  | Juel, K. | 2001 | Impact of tobacco, alcohol overconsumption and drug abuse on mortality in Denmark. Trends over 25 years, 1973-1997 | wrong outcome |
| Albareda et al | 2003 | Diabetes and abnormal glucose tolerance in women with previous gestational diabetes | Wrong study design |  | Jürgens et al | 2015 | Using lung cancer mortality to indirectly approximate smoking patterns in space | wrong study design |
| Albin et al | 1999 | Asbestos and cancer: An overview of current trends in Europe | Wrong outcome |  | Kaila-Kangas et al | 2016 | Alcohol-induced morbidity and mortality by occupation: a population-based follow-up study of working Finns | wrong outcome |
| Aleksandri at al | 2003 | Alcohol consumption and its connection with mortality from cardiovascular diseases in 40-59 years old men (data from 21.5 year prospective study) | Wrong outcome |  | Kallberg et al | 2011 | Smoking is a major preventable risk factor for rheumatoid arthritis: estimations of risks after various exposures to cigarette smoke | wrong outcome |
| Aleksandri at al | 2014 | Combined impact of healthy lifestyle factors on colorectal cancer: A large European cohort study | Wrong study design |  | Kark et al | 1995 | Are lean smokers at increased risk of lung cancer? The Israel civil servant cancer study | wrong outcome |
| Almasi at al | 2019 | Investigation of Climatic, Health and Economic Factors Affecting on Mortality in the Eastern Mediterranean Region | Wrong outcome |  | Kazakos et al | 2020 | Quantifying the health burden misclassification from the use of different PM2.5 exposure tier models: A case study of London | wrong outcome |
| Almeida at al | 2014 | Effects of exposure to particles and ozone on hospital admissions for cardiorespiratory diseases in SetÃºBal, Portugal | Wrong outcome |  | Kearns et al | 2014 | Chronic disease burden associated with overweight and obesity in Ireland: the effects of a small BMI reduction at population level | wrong outcome |
| Altieri at al | 2004 | Occupational and leisure time physical activity and the risk of nonfatal acute myocardial infarction in Italy | Wrong outcome |  | Kelly et al | 2009 | Mortality attributable to excess adiposity in England and Wales in 2003 and 2015: Explorations with a spreadsheet implementation of the Comparative Risk Assessment methodology | Wrong outcome |
| Anantharaman et al | 2011 | Population attributable risk of tobacco and alcohol for upper aerodigestive tract cancer | Wrong outcome |  | Kendall et al | 2011 | Numbers and proportions of leukemias in young people and adults induced by radiation of natural origin | wrong study design |
| Ancellin, R. and Bessette, D. | 2013 | Overweight, obesity and cancer risks | Wrong outcome |  | Key et al | 2006 | Meta-analysis of studies of alcohol and breast cancer with consideration of the methodological issues | Wrong study design |
| Anderson et al | 2003 | Particulate air pollution and hospital admissions for card iorespiratory diseases: are the elderly at greater risk? | Wrong outcome |  | Khaniabadi et al | 2017 | Human health risk assessment due to ambient PM10 and SO2 by an air quality modeling technique | Wrong outcome |
| Andersson et al | 2018 | Tackling the tobacco epidemic in the Nordic countries and lower cancer incidence by 1/5 in a 30-year periodâ€”The effect of envisaged scenarios changing smoking prevalence | Wrong outcome |  | Khreis et al | 2018 | Traffic-related air pollution and the local burden of childhood asthma in Bradford, UK | Wrong outcome |
| Andersson et al | 2019 | Avoidable cancers in the Nordic countries-the potential impact of increased physical activity on postmenopausal breast, colon and endometrial cancer | Wrong outcome |  | Kihal-Talantikite et al | 2019 | Premature Adult Death and Equity Impact of a Reduction of NO2, PM10, and PM2.5 Levels in Paris-A Health Impact Assessment Study Conducted at the Census Block Level | Wrong outcome |
| Andersson et al | 2018 | Avoidable cancers in the Nordic countries-The impact of alcohol consumption | Wrong outcome |  | Kikkenborg Berg et al | 2017 | Anxiety, depression and risk behaviour in cardiac patients. Findings from the national DenHeart survey | wrong study design |
| Andersson et al | 2017 | Avoidable cancer cases in the Nordic countries - The impact of overweight and obesity | Wrong outcome |  | Kim et al | 2013 | Influence of life-style choices on locomotor disability, arthritis and cardiovascular disease in older women: prospective cohort study | wrong study design |
| Andreasson et al | 1997 | Mortality and morbidity related to alcohol | Wrong outcome |  | Kim et al | 2016 | Attributable risk of lung cancer deaths due to indoor radon exposure | Wrong outcome |
| Andriolo et al | 2019 | Traditional risk factors for essential hypertension: analysis of their specific combinations in the EPIC-Potsdam cohort | Wrong study design |  | Kim et al | 2018 | Indoor radon and lung cancer: estimation of attributable risk, disease burden, and effects of mitigation | Wrong population |
| Antonsen et al | 2020 | Exposure to air pollution during childhood and risk of developing schizophrenia: a national cohort study | Wrong outcome |  | Kirk R. Smith, Sumi Mehta | 2003 | The burden of disease from indoor air pollution in developingcountries: comparison of estimates | wrong study design |
| Apte et al | 2015 | Addressing Global Mortality from Ambient PM2.5 | wrong outcome |  | Kivimäki et al | 2012 | Job strain as a risk factor for coronary heart disease: A collaborative meta-analysis of individual participant data | Wrong outcome |
| Arnedo et al | 2007 | Incidence of asthma and risk factors in a cohort of schoolchildren aged from 6-7 years old to 14-15 years old in Castellón (Spain) following the International Study of Asthma and Allergies in Childhood (ISAAC) | Wrong outcome |  | Knoops et al | 2004 | Mediterranean diet, lifestyle factors, and 10-year mortality in elderly European men and women: The HALE project | Wrong outcome |
| Arnesen et al | 2004 | Can the value choices in DALYs influence global priority-setting? | Wrong study design |  | Kocić et al | 1996 | Some insufficiently recognized risk factors for breast cancer | wrong study design |
| Arnold et al | 2018 | Global burden of cutaneous melanoma attributable to ultraviolet radiation in 2012 | Wrong outcome |  | Kogevinas et al | 2003 | Occupation and bladder cancer among men in Western Europe | wrong study design |
| Arnold et al | 2018 | Cutaneous melanoma in France in 2015 attributable to solar ultraviolet radiation and the use of sunbeds | Wrong outcome |  | Kogevinas et al | 1996 | The risk of asthma attributable to occupational exposures: A population- based study in Spain | wrong study design |
| Arnold et al | 2015 | Global burden of cancer attributable to high body-mass index in 2012: a population-based study | Wrong outcome |  | Kogevinas et al | 2007 | Exposure to substances in the workplace and new-onset asthma: an international prospective population-based study (ECRHS-II) | wrong study design |
| Arnold et al | 2018 | Cancers in France in 2015 attributable to high body mass index | Wrong outcome |  | Konnopka et al | 2011 | Health burden and costs of obesity and overweight in Germany | Wrong outcome (YPLL) |
| Arroyo et al | 2016 | Impact of air pollution and temperature on adverse birth outcomes: Madrid, 2001-2009 | Wrong outcome |  | Konnopka et al | 2009 | The health and economic consequences of moderate alcohol consumption in Germany 2002 | Wrong outcome (YPLL) |
| Asma et al | 2004 | Addressing the chronic disease burden with tobacco control programs | Wrong outcome |  | Kopel et al | 2013 | Mediterranean diet for primary prevention of cardiovascular disease[1] | No methodological information |
| Aune et al | 2016 | Nut consumption and risk of cardiovascular disease, total cancer, all-cause and cause-specific mortality: A systematic review and dose-response meta-analysis of prospective studies | wrong study design |  | Kraus et al | 2018 | Quantification of environmental burden of disease related to nitrogen dioxide exposure in Germany | No methodological information |
| Axelson, O. | 2002 | Alternative for estimating the burden of lung cancer from occupational exposures - Some calculations based on data from Swedish men | Wrong outcome |  | Kuijer et al | 2015 | Annual incidence of non-specific low back pain as an occupational disease attributed to whole-body vibration according to the National Dutch Register 2005-2012 | Wrong outcome |
| Baccini et al | 2011 | Health impact assessment of fine particle pollution at the regional level | Wrong outcome |  | Kulhánová et al | 2016 | The role of three lifestyle risk factors in reducing educational differences in ischaemic heart disease mortality in Europe | Wrong outcome |
| Baccini et al | 2015 | Commuting-adjusted short-term health impact assessment of airborne fine particles with uncertainty quantification via Monte Carlo simulation | wrong outcome |  | Kulhánová et al | 2018 | The fraction of lung cancer incidence attributable to fine particulate air pollution in France: Impact of spatial resolution of air pollution models | Wrong outcome |
| Bacevičienė et al | 2013 | Estimation of all-cause and cardiovascular mortality risk in relation to leisure-time physical activity: A cohort study | Wrong outcome |  | Kulhánová et al | 2020 | Proportion of cancers attributable to major lifestyle and environmental risk factors in the Eastern Mediterranean region | Wrong outcome |
| Badyda et al | 2018 | Cardiopulmonary diseases and lung cancer mortality due to PM2.5 exposure in 11 Polish Agglomerations in 20062015 | Wrong outcome |  | Kunze et al | 1993 | Outdoor air temperature and mortality in the Netherlands: A time-series analysis | wrong study design |
| Badyda et al | 2014 | Screening assessment of the burden of disease due to air pollution in eleven Polish agglomerations | Wrong outcome |  | Kunze et al | 1992 | Life style and occupational risk factors for bladder cancer in Germany: A case-control study | wrong study design |
| Badyda et al | 2017 | Ambient PM2.5 exposure and mortality due to lung cancer and cardiopulmonary diseases in polish cities | Wrong outcome |  | La Vecchia et al | 2001 | Vegetables, fruit, antioxidants and cancer: a review of Italian studies | wrong study design |
| Baecker et al | 2018 | Worldwide incidence of hepatocellular carcinoma cases attributable to major risk factors | Wrong outcome |  | La Vecchia et al | 2001 | Nutrition and health: epidemiology of diet, cancer and cardiovascular disease in Italy | wrong study design |
| Banegas Banegas et al | 2001 | Smoking-attributable deaths in Spain in 1998 | Wrong outcome |  | La Vecchia et al | 1995 | Attributable risks for stomach cancer in Northern Italy | Wrong outcome |
| Banegas Banegas et al | 1993 | Projections of the impact of the smoking habit on the health of the Spanish population and on the potential benefits from its control | Wrong outcome |  | La Vecchia et al | 1996 | Attributable risks for colorectal cancer in northern Italy | Wrong outcome |
| Banegas et al | 2005 | Recent decrease in smoking-attributable mortality in Spain | Wrong outcome |  | La Vecchia et al | 1997 | Body mass index and post-menopausal breast cancer: An age-specific analysis | wrong study design |
| Banegas et al | 2011 | Smoking-attributable deaths in Spain, 2006 | Wrong outcome |  | La Vecchia, C. | 2004 | Mediterranean diet and cancer | wrong study design |
| Banegas et al | 2010 | Prevalence and control of traditional cardiovascular risk factors and anticipated avoidable coronary mortality in primary prevention in Europe: The EURIKA study | No methodological information |  | La Vecchia, C. | 2013 | Fruit, vegetables and cancer risk | wrong study design |
| Banegas et al | 2003 | A simple estimate of mortality attributable to excess weight in the European Union | Wrong outcome |  | La Vecchia, C. and Bosetti, C. | 2006 | Diet and cancer risk in Mediterranean countries: open issues | wrong study design |
| Banegas et al | 2003 | Mortality attributable to cardiovascular risk factors in Spain | Wrong outcome |  | La Vecchia, C. and Tavani, A. | 1998 | Fruit and vegetables, and human cancer | wrong study design |
| Barbaglia et al | 2013 | The impact of common health conditions on disability in Europe | Wrong outcome |  | Laaksonen et al | 2010 | Estimation of population attributable fraction (PAF) for disease occurrence in a cohort study design | wrong outcome |
| Barbieri et al | 1999 | Incidence of malignant mesothelioma (1977-1996) and exposure to asbestos in a population of a lakeside area (Lake Iseo, Northern Italy) | Wrong study design |  | Lachenmeier, D. W. and Przybylski, M. C. | 2012 | Comparative risk assessment of carcinogens in alcoholic beverages using the margin of exposure approach | Wrong outcome |
| Barone-Adesi et al | 2005 | Population attributable risk for occupational cancer in Italy | Wrong outcome |  | Lachenmeier, D. W. and Rehm, J. | 2015 | Comparative risk assessment of alcohol, tobacco, cannabis and other illicit drugs using the margin of exposure approach | Wrong outcome |
| Barrera et al | 1999 | Female lung cancer & attributable risk to smoking in the Czech Rep., 1970-1995 | Wrong outcome |  | Lacourt et al | 2014 | Occupational and non-occupational attributable risk of asbestos exposure for malignant pleural mesothelioma | Wrong outcome |
| Bartosińska, M. and Ejsmont, J. | 2002 | Health condition of employees exposed to noise--extra auditory health effects | Wrong outcome |  | Lacourt et al | 2017 | Dose-time-response association between occupational asbestos exposure and pleural mesothelioma | Wrong outcome |
| Battisti et al | 2017 | Estimates of cancer deaths attributable to behavioural risk factors in Italy, 2013 | Wrong outcome |  | Lacourt et al | 2010 | Attributable risk in men in two French case-control studies on mesothelioma and asbestos | wrong study design |
| Bauman, A. | 1998 | Use of population attributable risk (PAR) in understanding the health benefits of physical activity | Wrong outcome |  | Laffoy et al | 2013 | Cancer incidence and mortality due to alcohol: An analysis of 10-year data | Wrong outcome |
| Becher et al | 2018 | Estimating lung cancer mortality attributable to second hand smoke exposure in Germany | Wrong outcome |  | Lanting et al | 2009 | Clustering of socioeconomic, behavioural, and neonatal risk factors for infant health in pregnant smokers | wrong study design |
| Behrens et al | 2018 | Cancers Due to Excess Weight, Low Physical Activity, and Unhealthy Diet | Wrong outcome |  | Law et al | 2016 | Cumulative radiation exposure and associated cancer risk estimates for scoliosis patients: Impact of repetitive full spine radiography | wrong study design |
| Bello et al | 2001 | Trend in the mortality attributable to tobacco on the Canary Islands (1975-1994) | Wrong outcome |  | Lawder et al | 2019 | Impact of maternal smoking on early childhood health: A retrospective cohort linked dataset analysis of 697 003 children born in Scotland 1997-2009 | Wrong outcome |
| Bendinelli et al | 2020 | Alcohol, smoking and rectal cancer risk in a Mediterranean cohort of adults: The European Prospective Investigation into Cancer and Nutrition (EPIC)-Italy cohort | Wrong outcome |  | Lear et al | 2017 | The effect of physical activity on mortality and cardiovascular disease in 130â€ˆ000 people from 17 high-income, middle-income, and low-income countries: the PURE study | Wrong outcome |
| Bergstrom et al | 2001 | Overweight as an avoidable cause of cancer in Europe | Wrong outcome |  | Lee et al | 2012 | Effect of physical inactivity on major non-communicable diseases worldwide: An analysis of burden of disease and life expectancy | Wrong outcome |
| Bernal et al | 2007 | Risk factors for suicidality in Europe: Results from the ESEMED study | Wrong outcome |  | Lee et al | 2009 | Systematic review of the relation between smokeless tobacco and cancer in Europe and North America | Wrong study design |
| Bernardo et al | 2019 | Relationships between air pollutants and mortality in Portugal-an environmental health assessment | No methodological information |  | Legrand Cattan et al | 2000 | Evaluation of occupational exposures in lung cancer | Wrong outcome |
| Bjørnelv et al | 2021 | Modelling childhood obesity in Norway - The MOON study | No methodological information |  | Lelieveld et al | 2020 | Loss of life expectancy from air pollution compared to other risk factors: a worldwide perspective | duplicate |
| Blanc et al | 2019 | The occupational burden of nonmalignant respiratory diseases an official American thoracic society and european respiratory society statement | Wrong outcome |  | Levi et al | 1996 | Alcohol and breast cancer in the Swiss Canton of Vaud | wrong study design |
| Blanc et al | 1999 | How much adult asthma can be attributed to occupational factors? | Wrong outcome |  | Levi et al | 1999 | Occupational and leisure-time physical activity and the risk of colorectal cancer | wrong study design |
| Blankenberg, S. | - | Large Scale Analysis of Lifetime Risk of Cardiovascular Disease in Europe and Population Attributable Risk of Cardiovascular Risk Factors for the Biomarkers for Cardiovascular Risk Assessment in Europe (BiomarCaRE) Investigators | Wrong study design |  | Licaj et al | 2016 | Epithelial ovarian cancer subtypes attributable to smoking in the Norwegian Women and Cancer Study, 2012 | Wrong outcome |
| Bobylev, N. | 2004 | Comparative risk assessment and environmental impact assessment: Similarity in quantitative methods | Wrong outcome |  | Lima et al | 2012 | Burden of disease attributable to risk factors in the northern region of Portugal | No methodological information |
| Bochicchio et al | 2013 | Quantitative evaluation of the lung cancer deaths attributable to residential radon: A simple method and results for all the 21 Italian Regions | Wrong outcome |  | Lindberg et al | 2006 | Prevalence and underdiagnosis of COPD by disease severity and attributable fraction of smoking. Report from the Obstructive Lung Disease in Northern Sweden Studies (vol 100, pg 264, 2006) | Wrong outcome |
| Bodenant et al | 2012 | Comparison of coronary heart disease and stroke risks attributable to vascular risk factors: Results from the prime study | No methodological information |  | Lissowska et al | 2003 | Smoking, alcohol, diet, dentition and sexual practices in the epidemiology of oral cancer in Poland | wrong outcome |
| Boffetta, P. | 2012 | The burden of lung cancer in non-smokers | No methodological information |  | Little et al | 2009 | Updated estimates of the proportion of childhood leukaemia incidence in Great Britain that may be caused by natural background ionising radiation | wrong study design |
| Boffetta, P. | 2010 | An estimate of cancers attributable to occupational exposures in France | Wrong outcome |  | Liutkute et al | 2017 | Burden of smoking in Lithuania: attributable mortality and years of potential life lost | Wrong outcome (YPLL) |
| Boffetta, P. | 2006 | The burden of cancer attributable to alcohol drinking | Wrong outcome |  | Lock, K. | 2005 | The Global Burden of Disease attributable to low fruit and vegetable intake - What does this mean for public health policy in Europe? | duplicate |
| Bonaldi et al | 2019 | Hospitalizations for cardiovascular diseases attributable to tobacco smoking in France in 2015 | Wrong outcome |  | López et al | 2016 | Mortality attributable to secondhand smoke exposure in Spain (2011) | wrong outcome |
| Borch et al | 2011 | Physical activity and mortality among Norwegian women - the Norwegian Women and Cancer study | Wrong outcome |  | Maag et al | 2013 | Direct estimation of death attributable to smoking in Switzerland based on record linkage of routine and observational data | wrong outcome |
| Borges et al | 2007 | Smoking attributable mortality in Portugal | Duplicate |  | Macera, C. A. and Powell, K. E. | 2001 | Population attributable risk: implications of physical activity dose | wrong outcome |
| Bosetti et al | 2000 | Fraction of prostate cancer incidence attributed to diet in Athens, Greece | Wrong outcome |  | Machii, R. and Saika, K. | 2012 | Mortality attributable to tobacco by region based on the WHO global report | wrong outcome |
| Boshuizen et al | 2017 | Taking multi-morbidity into account when attributing DALYs to risk factors: comparing dynamic modeling with the GBD2010 calculation method | Wrong study design |  | Magnani, C. and Leporati, M. | 1998 | Mortality from lung cancer and population risk attributable to asbestos in an asbestos cement manufacturing town in Italy | wrong outcome |
| Bovenzi et al | 1992 | Lung cancer and occupation: attributable risk in the province of Trieste | Wrong outcome |  | Manczuk et al | 2017 | Time trends in tobacco-Attributable cancer mortality in Poland - Direct estimation method | wrong outcome |
| Brát et al | 2015 | Dietary changes in relationship to risk factors and coronary heart disease mortality | Wrong outcome |  | Manczuk, M. | 2011 | Direct Estimation of Tobacco-Attributable Cancer Mortality in Poland | wrong outcome |
| Brauer et al | 2012 | Exposure assessment for estimation of the global burden of disease attributable to outdoor air pollution | Wrong outcome |  | Manthey et al | 2017 | Quantifying the global contribution of alcohol consumption to cardiomyopathy | wrong outcome |
| Briggs et al | 2017 | Health impact assessment of the UK soft drinks industry levy: a comparative risk assessment modelling study | Wrong outcome |  | Marant Micallef et al | 2019 | Cancers in France in 2015 attributable to occupational exposures | wrong outcome |
| Britton, A. and McPherson, K. | 2001 | Mortality in England and Wales attributable to current alcohol consumption | Wrong outcome |  | Marczak et al | 2018 | Global deaths attributable to high systolic blood pressure, 1990-2016 | wrong outcome |
| Brønnum-Hansen et al | 2001 | Abstention from smoking extends life and compresses morbidity: a population based study of health expectancy among smokers and never smokers in Denmark | Wrong study design |  | Marmet et al | 2016 | The importance of age groups in estimates of alcohol-attributable mortality: impact on trends in Switzerland between 1997 and 2011 | No methodological information |
| Brønnum-Hansen et al | 2002 | Healthy life years lost due to smoking | Duplicate |  | Marques-Vidal et al | 2011 | Burden of disease attributable to obesity and overweight in Switzerland | No methodological information |
| Brønnum-Hansen H. | 2000 | Predicted effect of smoking cessation of tobacco-related mortality | Wrong outcome |  | Martin et al | 2010 | Alcohol-attributable mortality in Ireland | Wrong outcome (PYLL) |
| Brown et al | 2018 | What proportion of cancers in the UK and its constituent countries could be prevented? An updated analysis | Wrong outcome |  | Martín-Ramiro et al | 2014 | Mortality attributable to excess weight in Spain | Wrong outcome |
| Bruffaerts et al | 2015 | Examination of the population attributable risk of different risk factor domains for suicidal thoughts and behaviors | Wrong outcome |  | Martins et al | 2019 | Burden of disease attributable to exposure to aflatoxins in Portugal using Human biomonitoring data | No methodological information |
| Campbell-Lendrum, D. and Woodruff, R. | 2006 | Comparative risk assessment of the burden of disease from climate change | Wrong outcome |  | Maugeri et al | 2020 | The association of social and behavioral factors with dietary risks in adults: Evidence from the Kardiovize Brno 2030 study | wrong study design |
| Cao et al | 2018 | Cancers attributable to tobacco smoking in France in 2015 | Wrong outcome |  | Mazloumi et al | 2019 | Avoidable Burden of Cardiovascular Diseases in the Eastern Mediterranean Region: Contribution of Selected Risk Factors for Cardiovascular-Related Deaths | Wrong population |
| Cao et al | 2019 | Mortality trends of colorectal cancer among overweight patients at the global and national levels | Wrong outcome |  | McKenzie et al | 2016 | Healthy lifestyle and risk of cancer in the European prospective investigation into cancer and nutrition cohort study | Wrong outcome |
| Cardis et al | 2006 | Estimates of the cancer burden in Europe from radioactive fallout from the Chernobyl accident | Wrong outcome |  | Medrano et al | 2007 | Coronary disease risk attributable to cardiovascular risk factors in the Spanish population | wrong outcome |
| Carreras et al | 2019 | Deaths from noncommunicable diseases attributable to behavioral risk factors in Italy and Italian regions, 2016 | Wrong outcome |  | Mehta et al | 2020 | Excess body weight, cigarette smoking, and type II diabetes incidence in the national FINRISK studies | Wrong outcome |
| Carreras et al | 2011 | Prediction of future smoking attributable deaths for lung cancer in Italy under primary and secondary prevention scenarios | Wrong study design |  | Mele et al | 1997 | Incidence of and risk factors for hepatitis A in Italy: Public health indications from a 10-year surveillance | Wrong outcome |
| Castro et al | 2020 | Comparing the lung cancer burden of ambient particulate matter using scenarios of air quality standards versus acceptable risk levels | Wrong outcome |  | Menvielle et al | 2018 | Tobacco-attributable burden of cancer according to socioeconomic position in France | Wrong outcome |
| Catalá-López, F. and Gènova-Maleras, R. | 2013 | Disease burden attributable to major risk factors in western European Countries: The challenge of controlling cardiovascular risk factors | No methodological information |  | Menzler et al | 2008 | Population attributable fraction for lung cancer due to residential radon in Switzerland and Germany | wrong outcome |
| Catelinois et al | 2006 | Lung cancer attributable to indoor radon exposure in France: Impact of the risk models and uncertainty analysis | Wrong outcome |  | Mezzetti et al | 1998 | Population attributable risk for breast cancer: Diet, nutrition, and physical exercise | Wrong outcome |
| Cerveri at al | 2010 | Women and smoking-related diseases: Mortality and morbidity in Italy and in the rest of the world | Wrong outcome |  | Micha et al | 2012 | Estimating the global and regional burden of suboptimal nutrition on chronic disease: methods and inputs to the analysis | wrong study design |
| Chakraborty et al | 2017 | Impact of high-BMI (body mass index) on non-communicable diseases in the European Union | No methodological information |  | Mir, L. | 2007 | The impact of major heat waves on all-cause and cause-specific mortality in France from 1971 to 2003 | Wrong outcome |
| Chakraborty et al | 2016 | Selected Non-Communicable Disease (NCD) burden attributable to dietary risks and low physical activity for the Republic of Ireland in 1990 and 2013 | No methodological information |  | Mons et al | 2018 | Cancers Due to Smoking and High Alcohol Consumption | Wrong outcome |
| Chakraborty et al | 2016 | Burden of cancer attributable to high Body Mass Index (BMI) in the Republic of Ireland for 1990 and 2013 | No methodological information |  | Mons et al | 2019 | Recalculation of Tobacco-Attributable Mortality: National and Regional Data for Germany | Wrong outcome |
| Chakraborty et al | 2016 | Contribution of tobacco use and dietary risks to the Cardio Vascular Disease (CVD) deaths in Ireland between 1990 and 2013 | No methodological information |  | Mons, U. | 2016 | Demographic ageing and the evolution of smokingattributable mortality: The example of Germany | Wrong outcome |
| Chambliss et al | 2014 | Estimating source-attributable health impacts of ambient fine particulate matter exposure: global premature mortality from surface transportation emissions in 2005 | Wrong outcome |  | Morfeld, P. and Erren, T. C. | 2017 | Premature deaths attributed to ambient air pollutants: let us interpret the Robins-Greenland theorem correctly | No methodological information |
| Chan-Yeung, M. | 2003 | Occupational asthma - Global perspective | Wrong outcome |  | Morgenstern et al | 2000 | Comparative risk assessment: An international comparison of methodologies and results | Wrong outcome |
| Cheng et al | 2013 | Current and Former Smoking and Risk for Venous Thromboembolism: A Systematic Review and Meta-Analysis | Wrong outcome |  | Mozaffarian et al | 2013 | The global impact of sodium consumption on cardiovascular mortality: A global, regional, and national comparative risk assessment | No methodological information |
| Cipriani et al | 1998 | Alcohol-related mortality in Italy | Wrong outcome (PYLL) |  | Mozaffarian et al | 2014 | Global sodium consumption and death from cardiovascular causes | wrong outcome |
| Coeurjolly et al | 2012 | Attributable risk estimation for adjusted disability multistate models: Application to nosocomial infections | Wrong outcome |  | Muñoz et al | 2002 | Evolution of the mortality attributable to alcohol consumption in Catalonia, 1988-1997 | Wrong outcome (PYLL) |
| Collaborators, G. B. D. Risk Factor | 2018 | Global, regional, and national comparative risk assessment of 84 behavioural, environmental and occupational, and metabolic risks or clusters of risks for 195 â€¦ | Duplicate |  | Nagelhout et al | 2012 | The effect of tobacco control policies on smoking prevalence and smoking-attributable deaths. Findings from the Netherlands SimSmoke Tobacco Control Policy Simulation Model | wrong study design |
| Collins et al | 2017 | Cancer incidence and mortality due to excess body weight in Ireland: An analysis of 10-year data | Wrong outcome |  | Nambiema et al | 2020 | Proportion of upper extremity musculoskeletal disorders attributable to personal and occupational factors: results from the French Pays de la Loire study | Wrong outcome |
| Continente et al | 2019 | Burden of respiratory disease attributable to secondhand smoke exposure at home in children in Spain (2015) | Wrong outcome |  | Näyhä et al | 2007 | Heat mortality in Finland in the 2000s | Wrong outcome |
| Continente et al | 2018 | Morbidity attributable to secondhand smoke exposure in children under 5 years old in Spain, 2015 | Duplicate |  | Negri et al | 1993 | Attributable risk for oral cancer in northern Italy | Wrong outcome |
| Coombs et al | 2015 | Physical inactivity among older adults: Implications for life expectancy among non-overweight and overweight or obese individuals | Wrong outcome |  | Negri et al | 1995 | Attributable risks for nonfatal myocardial infarction in Italy | Wrong outcome |
| Corrao et al | 1998 | Risk of inflammatory bowel disease attributable to smoking, oral contraception and breastfeeding in Italy: A nationwide case-control study | Wrong study design |  | Negri et al | 1992 | Attributable Risks for Esophageal Cancer in Northern Italy | Wrong outcome |
| Corrao et al | 1998 | Attributable risk for symptomatic liver cirrhosis in Italy | Wrong outcome |  | Neilson, A. and Schneider, H. | 2005 | Obesity and its comorbidities: Present and future importance on health status in Switzerland | Wrong outcome |
| Cortez-Pinto et al | 2009 | Liver disease is the main burden of illness attributable to alcohol drinking: Results of a national study | No methodological information |  | Nerriere et al | 2005 | Lung cancer risk assessment in relation with personal exposure to airborne particles in four French metropolitan areas | Wrong outcome |
| Cowie et al | 2013 | The global burden of liver disease attributable to hepatitis B, hepatitis C, and alcohol: Increasing mortality, differing causes | No methodological information |  | Nethery, R. C. and Dominici, F. | 2019 | Estimating pollution-attributable mortality at the regional and global scales: Challenges in uncertainty estimation and causal inference | No methodological information |
| Criado-Ãlvarez et al | 2002 | Mortality attributable to tobacco consumption in the years 1987 and 1997 in Castilla la Mancha, Spain | Wrong outcome |  | Neubauer et al | 2006 | Mortality, morbidity and costs attributable to smoking in Germany: Update and a 10-year comparison | Wrong outcome (YPLL) |
| Crosignani, P. | 2010 | [An estimate of health impacts of air pollution reduction] | Wrong outcome |  | Newson, R. B. | 2013 | Attributable and unattributable risks and fractions and other scenario comparisons | wrong study design |
| Cruise et al | 2019 | The Impact of Risk Factors for Coronary Heart Disease on Related Disability in Older Irish Adults | Wrong outcome |  | Nilsson et al | 2006 | Population-attributable risk of coronary heart disease risk factors during long-term follow-up: the Malmö Preventive Project | Wrong outcome |
| Cruts et al | 2008 | Morbidity due to smoking in the Netherlands: An estimated 90,000 clinical hospital admissions in 2005 | Wrong outcome |  | Nizard, A. and Munozperez, F. | 1994 | Alcohol, Tobacco, and Mortality in France since 1950 - an Estimate of the Annual Numbers of Deaths | Wrong outcome |
| Currie et al | 2013 | The effect of tobacco control policies on smoking prevalence and smoking-attributable deaths in Ireland using the IrelandSS simulation model | Wrong outcome |  | Nordahl et al | 2013 | Assessment of mediation by behavioral risk factors on educational-related gradients in cause-specific mortality using additive hazards modeling: A multicenter cohort study | No methodological information |
| Danaei et al | 2006 | Global and regional mortality from ischaemic heart disease and stroke attributable to higher-than-optimum blood glucose concentration: comparative risk assessment | Wrong outcome |  | Nurminen, M. M. and Jaakkola, M. S. | 2001 | Mortality from occupational exposure to environmental tobacco smoke in Finland | wrong outcome |
| Danaei et al | 2014 | Cardiovascular disease, chronic kidney disease, and diabetes mortality burden of cardiometabolic risk factors from 1980 to 2010: A comparative risk assessment | Wrong outcome |  | Odlaug et al | 2015 | Alcohol dependence, co-occurring conditions and attributable burden | Wrong outcome |
| Danaei et al | 2005 | Causes of cancer in the world: Comparative risk assessment of nine behavioural and environmental risk factors | Wrong outcome |  | O'Donnell et al | 2016 | Global and regional effects of potentially modifiable risk factors associated with acute stroke in 32 countries (INTERSTROKE): a case-control study | Wrong outcome |
| D'Avanzo et al | 1995 | Attributable risks for bladder cancer in Northern Italy | Wrong outcome |  | O'Keeffe et al | 2013 | Modelling the impact of specific food policy options on Coronary heart disease and stroke deaths in Ireland | Wrong outcome |
| Davin et al | 2012 | Cardiovascular risk factors attributable to obesity and overweight in Switzerland | Wrong outcome |  | Oliveira et al | 2008 | Aspects of tobacco attributable mortality: systematic review | wrong outcome |
| De Vries et al | 2010 | Lifestyle changes and reduction of colon cancer incidence in Europe: A scenario study of physical activity promotion and weight reduction | Wrong outcome |  | Oliveira et al | 2009 | Impact of risk factors for non-fatal acute myocardial infarction | wrong outcome |
| Decković-Vukres et al | 2009 | Incidence and prevalence of asbestos-related diseases in Croatia | Wrong outcome |  | Olsson et al | 2011 | Lung cancer risk attributable to occupational exposures in a multicenter case-control study in central and Eastern Europe | Wrong outcome |
| Degenhardt et al | 2014 | The global epidemiology and burden of opioid dependence: results from the global burden of disease 2010 study | Wrong study design |  | Onat, A. | 2003 | The impact of obesity on cardiovascular diseases in Turkey | Wrong outcome |
| Degenhardt et al | 2013 | Global burden of disease attributable to illicit drug use and dependence: Findings from the Global Burden of Disease Study 2010 | Wrong study design |  | O'Neill et al | 2017 | Impact of sugar sweetened beverages on incidence of type 2 diabetes in Ireland | No methodological information |
| Di Maso et al | 2020 | Attributable fraction for multiple risk factors: Methods, interpretations, and examples | wrong outcome |  | O'Reilly et al | 2014 | Cancer incidence and mortality due to alcohol in Ireland (2001-2010) | No methodological information |
| Diaz et al | 2019 | Mortality attributable to high temperatures over the 2021-2050 and 2051-2100 time horizons in Spain: Adaptation and economic estimate | wrong outcome |  | Ortiz et al | 2017 | Evaluation of short-term mortality attributable to particulate matter pollution in Spain | wrong outcome |
| Dinu et al | 2009 | Lung Cancer Attributable to Indoor Radon Exposures in Two Radon - Prone Areas, Stei (Romania) and Torrelodones (Spain) | wrong outcome |  | Oudin Åström et al | 2018 | Investigating changes in mortality attributable to heat and cold in Stockholm, Sweden | Wrong outcome |
| Doidge et al | 2012 | Attributable risk analysis reveals potential healthcare savings from increased consumption of dairy products | wrong outcome |  | Öztürk et al | 2010 | Epidemiology of cerebrovascular diseases and risk factors-perspectives of the world and Turkey | Wrong outcome |
| Dunlop et al | 2013 | The fraction of cancers attributable to tobacco in Wales, in 2012 | wrong outcome |  | Palazzo et al | 2012 | Respective Contribution of Chronic Conditions to Disability in France: Results from the National Disability-Health Survey | wrong outcome |
| Eatough, J. P. and Henshaw, D. L. | 1995 | The theoretical risk of non-melanoma skin cancer from environmental radon exposure | wrong outcome |  | Palazzo et al | 2019 | Methods to assess the contribution of diseases to disability using cross-sectional studies: Comparison of different versions of the attributable fraction and the attribution method | Wrong outcome |
| Effertz et al | 2016 | The costs and consequences of obesity in Germany: a new approach from a prevalence and life-cycle perspective | wrong outcome |  | Palmer et al | 2002 | Occupational exposure to noise and the attributable burden of hearing difficulties in Great Britain | Wrong outcome |
| Ekelund et al | 2013 | Physical activity, general and abdominal obesity and mortality in European men and women | wrong outcome |  | Palmer et al | 2003 | The relative importance of whole body vibration and occupational lifting as risk factors for low-back pain | wrong outcome |
| Ekelund et al | 2015 | Physical activity and all-cause mortality across levels of overall and abdominal adiposity in European men and women: The European prospective investigation into cancer and nutrition study (EPIC) | wrong outcome |  | Parazzini et al | 2000 | Population attributable risk for ovarian cancer | wrong outcome |
| El Fray, I. | 2012 | A Comparative Study of Risk Assessment Methods, MEHARI & CRAMM with a New Formal Model of Risk Assessment (FoMRA) in Information Systems | Wrong outcome |  | Parazzini, F. | 1993 | Italian infant mortality attributable to low birthweight | No methodological information |
| Eliasen et al | 2013 | Alcohol-attributable and alcohol-preventable mortality in Denmark: An analysis of the impact of different intake levels on mortality | wrong outcome |  | Parkin, D. M. | 2011 | 1. The fraction of cancer attributable to lifestyle and environmental factors in the UK in 2010 | wrong outcome |
| Eliasen et al | 2003 | Diabetes and obesity in Northern Sweden: occurrence and risk factors for stroke and myocardial infarction | wrong outcome |  | Parkin, D. M. | 2011 | Tobacco-attributable cancer burden in the UK in 2010 | wrong outcome |
| Eriksen et al | 2015 | The impact of health behaviours on incident cardiovascular disease in Europeans and South Asians - A prospective analysis in the UK SABRE study | wrong study design |  | Pascal et al | 2013 | Assessing the public health impacts of urban air pollution in 25 European cities: Results of the Aphekom project | wrong study design |
| Erren, T. C. and Morfeld, P. | 2011 | Attributing the burden of cancer at work: Three areas of concern when examining the example of shift-work | Wrong study design |  | Patel et al | 2014 | Global and regional trends in mortality from chronic obstructive pulmonary disease: Their relation to poverty, smoking and population change | wrong outcome |
| Evlampidou et al | 2020 | Trihalomethanes in drinking water and bladder cancer burden in the European Union | wrong outcome |  | Patz et al | 2008 | Health impact assessment of global climate change: expanding on comparative risk assessment approaches for policy making | Wrong study design |
| Faeh et al | 2011 | Obesity but not overweight is associated with increased mortality risk | wrong outcome |  | Peleteiro et al | 2015 | Worldwide Burden of Gastric Cancer Attributable to Tobacco Smoking in 2012 and Predictions for 2020 | Wrong outcome |
| Fakhfakh | 2011 | Mortality due to smoking in Tunisia in 1997 | Wrong population |  | Peltonen et al | 2017 | Contribution of smoking-attributable mortality to life expectancy differences by marital status among Finnish men and women, 1971-2010 | Wrong outcome |
| Fernandez et al | 1996 | Attributable risks for pancreatic cancer in Northern Italy | wrong outcome |  | Pérez-Ríos et al | 2009 | Mortality associated to tobacco consumption in Galicia, Spain, 2001-2006 | Wrong outcome |
| Fierro et al | 2010 | Premature death and potential years of life lost due to alcohol consumption in Spain and the different autonomous communities in 2004 | wrong outcome |  | Pomerleau et al | 2003 | The burden of disease attributable to nutrition in Europe | wrong study design |
| Fihel, A. and Muszyńska, M. M. | 2015 | The regional variation in tobacco smoking - attributable mortality in Poland, 2006-2010 | wrong outcome |  | Praud et al | 2016 | Cancer incidence and mortality attributable to alcohol consumption | Wrong outcome |
| Fischer et al | 2004 | Air pollution related deaths during the 2003 heat wave in the Netherlands | wrong outcome |  | Preston et al | 2010 | A new method for estimating smoking-attributable mortality in high-income countries | Wrong outcome |
| Fleri-Soler et al | 2018 | Pollution and cardiovascular health in Malta - A review | wrong outcome |  | Radoï et al | 2013 | Tobacco smoking, alcohol drinking and risk of oral cavity cancer by subsite: Results of a French population-based case-control study, the ICARE study | Wrong outcome |
| Forastiere et al | 2005 | The proportion of respiratory disorders in childhood attributable to preventable and not preventable risk factors | wrong outcome |  | Redon et al | 2016 | Impact of hypertension on mortality and cardiovascular disease burden in patients with cardiovascular risk factors from a general practice setting: The ESCARVAL-risk study | wrong outcome |
| Forastiere et al | 2020 | Assessing short-term impact of PM10 on mortality using a semiparametric generalized propensity score approach | wrong outcome |  | Rehm et al | 2013 | The comparative risk assessment for alcohol as part of the Global Burden of Disease 2010 Study: What changed from the last study? | wrong study design |
| Foreman et al | 2018 | Forecasting life expectancy, years of life lost, and all-cause and cause-specific mortality for 250 causes of death: reference and alternative scenarios for 2016â€“40 for 195 countries and territories | Wrong study design |  | Rehm et al | 2013 | Global burden of alcoholic liver diseases | Wrong outcome (PYLL) |
| Frentzel-Beyme, R. | 2005 | The burden to health due to Diesel emissions in the EU. A survey of the current situation | Wrong outcome |  | Rehm et al | 2013 | Modeling the impact of alcohol dependence on mortality burden and the effect of available treatment interventions in the European Union | wrong study design |
| Frost et al | 2011 | The effect of smoking on the risk of lung cancer mortality for asbestos workers in Great Britain (1971-2005) | Wrong outcome |  | Rehm et al | 2016 | Modelling the impact of alcohol consumption on cardiovascular disease mortality for comparative risk assessments: an overview | Wrong outcome |
| Furtunescu et al | 2009 | Alcohol consumption impact on premature mortality in Romania | Wrong outcome |  | Rehm et al | 2018 | Alcohol dependence and very high risk level of alcohol consumption: a life-threatening and debilitating disease | Wrong outcome |
| Gaizauskiene et al | 2003 | Risk factors of perinatal mortality in Lithuania, 1997-1998 | Wrong outcome |  | Remy et al | 2011 | Health impact of urban air pollution in Belgium | Wrong outcome |
| Gajalakshmi et al | 2000 | Global patterns of smoking and smoking-attributable mortality | Wrong outcome |  | Renehan et al | 2010 | Interpreting the epidemiological evidence linking obesity and cancer: A framework for population-attributable risk estimations in Europe | wrong outcome |
| Gallus et al | 2011 | Smoking prevalence and smoking attributable mortality in Italy, 2010 | Wrong outcome |  | Renehan et al | 2010 | Incident cancer burden attributable to excess body mass index in 30 European countries | Wrong outcome |
| Gandini et al | 2014 | Melanoma attributable to sunbed use and tan seeking behaviours: An Italian survey | Wrong outcome |  | Rentería et al | 2016 | The impact of cigarette smoking on life expectancy between 1980 and 2010: a global perspective | wrong outcome |
| Ganne-Carrié et al | 2018 | Estimate of hepatocellular carcinoma incidence in patients with alcoholic cirrhosis | Wrong outcome |  | Rey et al | 2010 | Estimating the number of alcohol-attributable deaths: Methodological issues and illustration with French 2006 data | Wrong outcome (YPLL) |
| García García et al | 2007 | Estimate of the mortality rate attributable to occupational diseases in Spain, 2004 | Wrong outcome |  | Rivera et al | 2019 | Associations of major depressive disorder with chronic physical conditions, obesity and medication use: Results from the PISMA-ep study | wrong outcome |
| Gaskin et al | 2018 | Global estimate of lung cancer mortality attributable to residential radon | Wrong outcome |  | Rodriguez Tapioles et al | 1997 | Morbidity, mortality and the potential years of life lost attributable to tobacco | Wrong outcome (PYLL) |
| Gasparrini et al | 2012 | The effect of high temperatures on cause-specific mortality in England and Wales | Wrong outcome |  | Rodu, B. and Cole, P. | 2004 | The burden of mortality from smoking: Comparing Sweden with other countries in the European Union | wrong outcome |
| Gasparrini et al | 2015 | Mortality risk attributable to high and low ambient temperature: A multicountry observational study | Wrong outcome |  | Roerecke et al | 2007 | Alcohol and burden of disease in Switzerland: Implications for policy | duplicate |
| Gasparrini, A. and Leone, M. | 2014 | Attributable risk from distributed lag models | Wrong outcome |  | Roglic et al | 2005 | The burden of mortality attributable to diabetes: realistic estimates for the year 2000 | wrong outcome |
| Gdalevich et al | 2008 | Designing a Methodology to Measure the Health Effects of Air Pollution in Southern Israel: Estimation of Attributable Morbidity, Mortality and Healthcare Consumption | wrong study design |  | Romanens et al | 2011 | Population attributable coronary risk is mainly driven by LDL-cholesterol: Similar observations in two distinct healthy populations | No methodological information |
| Gefeller, O. | 1992 | An annotated bibliography on the attributable risk | wrong study design |  | Romanens et al | 2011 | Population attributable stroke risk is usually low: Similar observations in two distinct healthy populations | No methodological information |
| Gefeller, O. and Windeler, J. | 1991 | Risk factors for cervical cancer: comments on attributable risk calculations and the evaluation of screening in case-control studies | wrong study design |  | Rosato et al | 2015 | Population attributable risk for pancreatic cancer in Northern Italy | wrong outcome |
| Geleijnse et al | 2004 | Impact of dietary and lifestyle factors on the prevalence of hypertension in Western populations | Wrong outcome |  | Rožeková et al | 2019 | Estimating the impact of overweight and obesity on cancer risk in the Czech and Slovak populations | Wrong outcome |
| Georgousopoulou et al | 2014 | The role of dietary patterns' assessment in the predictive ability of cardiovascular disease risk estimation models: A review | Wrong outcome |  | Rushton et al | 2008 | The burden of cancer at work: Estimation as the first step to prevention | wrong outcome |
| Giannini et al | 2017 | Estimating deaths attributable to airborne particles: sensitivity of the results to different exposure assessment approaches | Wrong outcome |  | Rushton et al | 2012 | Occupational cancer burden in Great Britain | wrong outcome |
| Gillies et al | 2017 | Mortality from circulatory diseases and other non-cancer outcomes among nuclear workers in France, the United Kingdom and the United States (inworks) | Wrong outcome |  | Rushton et al | 2011 | The British occupational cancer burden study | No methodological information |
| Ginsberg et al | 2016 | Mortality, hospital days and expenditures attributable to ambient air pollution from particulate matter in Israel | Wrong outcome |  | Rushton et al | 2012 | The number of cancers caused by occupation in the UK | No methodological information |
| Ginsberg et al | 2010 | Issues in estimating smoking attributable mortality in Israel | Wrong outcome |  | Rushton, L. and Hutchings, S. J. | 2017 | The burden of occupationally-related cutaneous malignant melanoma in Britain due to solar radiation | wrong outcome |
| Ginsberg, G. M. and Geva, H. | 2014 | The burden of smoking in Israel-attributable mortality and costs (2014) | Wrong outcome |  | Sadetzki, S. | 2007 | Excess lifetime cancer mortality risk attributed to radiation exposure from pediatric computed tomography scan | wrong study design |
| Ginsberg, G. M. and Geva, H. | 2016 | Erratum to: The burden of smoking in Israel-attributable mortality and costs (2014). Isr J Health Policy Res. 2014;3:28 | duplicate |  | Saika, K. and Machii, R. | 2012 | Cancer mortality attributable to tobacco by region based on the who global report | wrong outcome |
| Glorennec et al | 2002 | Health impact assessment of brief exposures to urban air pollution in northwestern France | Wrong outcome |  | Samet, J. M. | 2016 | The burden of disease from air pollution in Israel: How do we use burden estimates to advance public health | No methodological information |
| Glorennec, P. and Monroux, F. | 2007 | Health impact assessment of PM10 exposure in the city of Caen, France | Wrong outcome |  | Schottenfeld Et al | 2013 | Current perspective on the global and United States cancer burden attributable to lifestyle and environmental risk factors | wrong outcome |
| Gmel et al | 2013 | The effects of capping the alcohol consumption distribution and relative risk functions on the estimated number of deaths attributable to alcohol consumption in the European Union in 2004 | Wrong outcome |  | Schutze et al | 2010 | Alcohol-Attributable Burden of Cancer Incidence in 8 European Countries | duplicate |
| Goldbourt et al | 1997 | Isolated low HDL cholesterol as a risk factor for coronary heart disease mortality: A 21-year follow-up of 8000 men | Wrong outcome |  | Schütze et al | 2011 | Alcohol attributable burden of incidence of cancer in eight European countries based on results from prospective cohort study | wrong outcome |
| Gorini et al | 2003 | Impact of smoking in Italy in 1998: deaths and years of potential life lost | Wrong outcome |  | Schwarzinger et al | 2017 | Alcohol use disorders and associated chronic disease - a national retrospective cohort study from France | wrong outcome |
| Graciani et al | 2008 | Cardiovascular mortality attributable to high blood pressure in Spanish population over 50 | Wrong outcome |  | Shield et al | 2018 | New cancer cases attributable to diet among adults aged 30-84 years in France in 2015 | wrong outcome |
| Graells, M. A. and Garcia, P. G. | 1998 | Alcohol related mortality in Catalonia: 1994 | Wrong outcome |  | Shield et al | 2012 | An assessment of methodologies to estimate the burden of injuries attributable to alcohol consumption | wrong study design |
| Gram et al | 2009 | Cigarette smoking and risk of colorectal cancer among Norwegian women | Wrong outcome |  | Sipetic-Grujicic et al | 2012 | Burden of ischaemic heart disease and stroke in Serbia | No methodological information |
| Gram et al | 2016 | The smoking related risk of breast cancer and proportion of avoidable breast cancer cases due to passive and active smoking in middle-aged women in Norway in 2012: The Norwegian women and cancer study 1991-2012 | No methodological information |  | Smith et al | 2002 | The global burden of disease from indoor air pollution: results from comparative risk assessment | wrong study design |
| Grau et al | 2010 | Why should population attributable fractions be periodically recalculated?. An example from cardiovascular risk estimation in southern Europe | Wrong outcome |  | Soerjomataram et al | 2018 | Cancers related to lifestyle and environmental factors in France in 2015 | wrong outcome |
| Gredner et al | 2018 | Cancers Due to Infection and Selected Environmental Factors | Wrong outcome |  | Soler et al | 1998 | Diet, alcohol, coffee and pancreatic cancer: Final results from an Italian study | wrong outcome |
| Gross, A. J. | 1998 | The risk of coronary heart disease in non-smokers exposed to environmental tobacco smoke | Wrong outcome |  | Sovinová et al | 2008 | Smoking-attributable mortality in the Czech Republic | wrong outcome |
| Guallar, E. | 2011 | Excess risk attributable to traditional cardiovascular risk factors in clinical practice settings across Europe. The EURIKA Study | Wrong outcome |  | Spirtas et al | 1994 | Malignant mesothelioma: attributable risk of asbestos exposure | wrong outcome |
| Guérin et al | 2013 | Alcohol-attributable mortality in France | Wrong outcome |  | Stefler et al | 2018 | Smoking, alcohol and cancer mortality in Eastern European men: Findings from the PrivMort retrospective cohort study | wrong outcome |
| Guida et al | 2011 | Risk of lung cancer and occupational history: Results of a french population-based case-control study, the ICARE study | Wrong outcome |  | Steindorf et al | 1995 | Lung cancer deaths attributable to indoor radon exposure in West Germany | Wrong outcome |
| Gupta et al | 2019 | Risk of coronary heart disease among smokeless tobacco users: Results of systematic review and meta-analysis of global data | Wrong outcome |  | T Mannetje et al | 2011 | Occupational exposure to metal compounds and lung cancer. Results from a multi-center case-control study in Central/Eastern Europe and UK | Wrong outcome |
| Gustavsson et al | 2003 | Calculation of fractions of lung cancer incidence attributable to occupational exposure to asbestos and combustion products in Stockholm, Sweden | Wrong outcome |  | T Mannetje et al | 1999 | Occupation and bladder cancer in European women | Wrong outcome |
| Gutiérrez-Abejón et al | 2015 | Smoking impact on mortality in Spain in 2012 | Wrong outcome |  | Tavani et al | 2006 | Consumption of sweet foods and breast cancer risk in Italy | wrong outcome |
| Gutjahr, E. and Gmel, G. | 2005 | Association of alcohol consumption to mortality and person-years of life lost in Switzerland - Measuring the impact of some methodological options | Wrong outcome (PYLL) |  | Tavani, A. and La Vecchia, C. | 1995 | Fruit and vegetable consumption and cancer risk in a Mediterranean population | wrong outcome |
| Gvinianidze, K. and Tsereteli, D. | 2012 | Tobacco smoking attributable mortality and years of potential life lost in Georgia | Wrong outcome (PYLL) |  | Testino, G. | 2011 | The burden of cancer attributable to alcohol consumption | wrong study design |
| Haapanen-Niemi et al | 1999 | Public health burden of coronary heart disease risk factors among middle-aged and elderly men | Wrong outcome |  | Tobollik et al | 2018 | [Environmental burden of disease in Germany] | wrong study design |
| Hajat et al | 2004 | Ethnic differences in risk factors for ischemic stroke: A European case-control study | Wrong outcome |  | Tobollik et al | 2018 | [The Environmental Burden of Disease Concept] | No methodological information |
| Hammami et al | 2018 | The burden of hypertension and associated risk for cardiovascular mortality in the UK biobank | Wrong outcome |  | Torén, K. and Blanc, P. D. | 2009 | Asthma caused by occupational exposures is common-a systematic analysis of estimates of the population-attributable fraction | Wrong outcome |
| Harriss et al | 2009 | Lifestyle factors and colorectal cancer risk (2): A systematic review and meta-analysis of associations with leisure-time physical activity | Wrong outcome |  | Toschke et al | 2007 | Adjusted population attributable fractions and preventable potential of risk factors for childhood obesity | Wrong outcome |
| Harriss et al | 2009 | Lifestyle factors and colorectal cancer risk (1): Systematic review and meta-analysis of associations with body mass index | Wrong outcome |  | Touillaud et al | 2019 | Cancers in France in 2015 attributable to insufficient physical activity | Wrong outcome |
| Hashibe et al | 2007 | Contribution of tobacco and alcohol to the high rates of squamous cell carcinoma of the supraglottis and glottis in Central Europe | Wrong outcome |  | Van Der Bij et al | 2016 | Expected number of asbestos-related lung cancers in the Netherlands in the next two decades: A comparison of methods | Wrong outcome |
| Hashim, D. and Boffetta, P. | 2014 | Occupational and Environmental Exposures and Cancers in Developing Countries | Wrong outcome |  | Van Der Molen et al | 2019 | How to improve the assessment of the impact of occupational diseases at a national level? The Netherlands as an example | Wrong outcome |
| Hedlund et al | 2012 | Occupational air pollutants-More hazardous for respiratory health than smoking? Report from the obstructive lung disease in northern Sweden studies | Wrong outcome |  | van Gemert et al | 2015 | The proportion of postmenopausal breast cancer cases in the Netherlands attributable to lifestyle-related risk factors | Wrong outcome |
| Heidemann et al | 2007 | Potentially modifiable classic risk factors and their impact on incident myocardial infarction: Results from the EPIC-Potsdam study | Wrong outcome |  | Vidra et al | 2018 | Impact of different estimation methods on obesity-attributable mortality levels and trends: The case of the Netherlands | Wrong outcome |
| Heidrich et al | 2006 | Estimate of deaths attributable to passive smoking in Germany - A sensitivity analysis | No methodological information |  | Vineis et al | 2007 | Lung cancers attributable to environmental tobacco smoke and air pollution in non-smokers in different European countries: A prospective study | wrong outcome |
| Heidrich et al | 2003 | Classical risk factors for myocardial infarction and total mortality in the community - 13-Year follow-up of the MONICA Augsburg cohort study | wrong study design |  | Walker et al | 2009 | Global and regional child mortality and burden of disease attributable to zinc deficiency | Wrong population |
| Heidrich et al | 2007 | Mortality and morbidity from coronary heart disease attributable to passive smoking | Wrong outcome |  | Walter et al | 2012 | The burden of disease att ributable to physical inactivity in the Austrian region of burgenland | No methodological information |
| Hein et al | 1996 | Alcohol consumption, serum low density lipoprotein cholesterol concentration, and risk of ischaemic heart disease: Six year follow up in the Copenhagen male study | wrong study design |  | Wichmann et al | 2006 | Lung cancer risk in Germany attributable to radon in homes | No methodological information |
| Hein et al | 2001 | Lewis phenotypes, leisure time physical activity, and risk of ischaemic heart disease: An 11 year follow up in the Copenhagen male study | wrong study design |  | Wienecke et al | 2014 | Changes in cancer incidence attributable to tobacco smoking in Germany, 1999-2008 | wrong outcome |
| Heintjes et al | 2011 | Population attributable risk (PAR) of macrovascular events associated with hba1c, blood pressure or weight in patients with type 2 diabetes mellitus: Evidence from a Dutch cohort | wrong outcome |  | Wienecke et al | 2013 | Preventability estimates for colorectal and breast cancer in Germany: A methodological evaluation of the risk factors alcohol and overweight | Wrong outcome |
| Helleberg et al | 2014 | Risk of cancer among HIV patients compared to the background population: Impact of smoking and HIV | wrong outcome |  | Wienecke et al | 2018 | Cancers Potentially Preventable through Excess Weight Reduction in Germany in 2010 | wrong outcome |
| Henneberger et al | 2010 | The occupational contribution to severe exacerbation of asthma | wrong outcome |  | Yang et al | 2018 | Global mortality burden of cirrhosis and liver cancer attributable to injection drug use, 1990â€“2016: An age-period-cohort and spatial autocorrelation analysis | wrong outcome |
| Hernandez et al | 2011 | Low-to-moderate levels of overweight predict the incidence of cardiovascular events: The Spanish SUN cohort | wrong study design |  | Zorrilla-Torras et al | 2005 | Smoking attributable mortality in the community of Madrid: 1992-1998 | wrong outcome |
| Hernandez-Garcia et al | 2010 | Mortality attributable to smoking in Spain in 2006 | wrong outcome |  |  | 2011 | 20th European Stroke Conference, ESC 2011 | No methodological information |
| Heuschmann et al | 2007 | Stroke mortality and morbidity attributable to passive smoking in Germany | wrong outcome |  |  | 2015 | Update of mortality attributable to diabetes for the IDF Diabetes Atlas: Estimates for the year 2013 | Wrong outcome |
| Hildrum et al | 2009 | Metabolic syndrome and risk of mortality in middle-aged versus elderly individuals: The Nord-Trøndelag Health Study (HUNT) | wrong study design |  |  | 2018 | Burden of diarrhea in the Eastern Mediterranean Region, 1990-2015: Findings from the Global Burden of Disease 2015 study | Wrong population |
| Hill, C. | 1993 | Mortality from tobacco use | wrong outcome |  |  | 2018 | Burden of obesity in the Eastern Mediterranean Region: findings from the Global Burden of Disease 2015 study | Wrong population |
| Hill, C. | 1998 | Trends in tobacco smoking and consequences on health in France | wrong outcome |  |  | 2018 | Burden of lower respiratory infections in the Eastern Mediterranean Region between 1990 and 2015: findings from the Global Burden of Disease 2015 study | Wrong population |
| Hill, C. | 2012 | Tobacco epidemiology | wrong study design |  |  |  |  |  |
| Hjort et al | 2018 | Overweight, obesity and the risk of LADA: results from a Swedish caseâ€“control study and the Norwegian HUNT Study | wrong study design |  |  |  |  |  |
| Hjort et al | 2013 | Overweight is associated with LADA among women but not in men: Results from ESTRID, a Swedish case-control study | wrong study design |  |  |  |  |  |
| Hoffmeister et al | 2010 | Male sex and smoking have a larger impact on the prevalence of colorectal neoplasia than family history of colorectal cancer | wrong outcome |  |  |  |  |  |
| Hu et al | 2005 | The effects of physical activity and body mass index on cardiovascular, cancer and all-cause mortality among 47 212 middle-aged Finnish men and women | wrong outcome |  |  |  |  |  |
| Hubbard et al | 1996 | Occupational exposure to metal or wood dust and aetiology of cryptogenic fibrosing alveolitis | wrong outcome |  |  |  |  |  |
| Huber et al | 2020 | Temperature-related excess mortality in German cities at 2 degreeC and higher degrees of global warming | wrong outcome |  |  |  |  |  |
| Huerta et al | 2010 | Cardiovascular risk estimated after 13 years of follow-up in a low-incidence Mediterranean region with high-prevalence of cardiovascular risk factors | wrong study design |  |  |  |  |  |
| Hughes et al | 2014 | Chronic obstructive pulmonary disease (COPD) case-finding and tobacco dependence on long stay psychiatric wards | wrong study design |  |  |  |  |  |
| Hutchings et al | 2014 | Estimating the burden of occupational cancer taking into account age0261 Estimating the burden of occupational cancer taking into account age | wrong outcome |  |  |  |  |  |
| Hutchings et al | 2013 | Estimating the burden of occupational Chronic Obstructive Pulmonary Disease (COPD) in the UK | wrong outcome |  |  |  |  |  |
| Hutchings, S. and Rushton, L. | 2011 | Toward risk reduction: predicting the future burden of occupational cancer | wrong outcome |  |  |  |  |  |
| Hutchings, S. J. and Rushton, L. | 2012 | Occupational cancer in Britain: Statistical methodology | wrong outcome |  |  |  |  |  |
| Huynen, M. M. T. E. and Martens, P. | 2015 | Climate change effects on heat- and cold-related mortality in the Netherlands: A scenario-based integrated environmental health impact assessment | wrong outcome |  |  |  |  |  |

[1] B. Ádám, Á. Molnár, G. Gulis, and R. Ádány, “Integrating a quantitative risk appraisal in a health impact assessment: analysis of the novel smoke-free policy in Hungary,” *Eur. J. Public Health*, vol. 23, no. 2, pp. 211–217, Apr. 2013, doi: 10.1093/eurpub/cks018.

[2] E. E. Agardh *et al.*, “Burden of type 2 diabetes attributed to lower educational levels in Sweden,” *Popul. Health Metr.*, vol. 9, p. 60, Dec. 2011, doi: 10.1186/1478-7954-9-60.

[3] E. E. Agardh *et al.*, “Alcohol-attributed disease burden in four Nordic countries: a comparison using the Global Burden of Disease, Injuries and Risk Factors 2013 study,” *Addict. Abingdon Engl.*, vol. 111, no. 10, pp. 1806–1813, Oct. 2016, doi: 10.1111/add.13430.

[4] E. Agardh, T. Moradi, and P. Allebeck, “[The contribution of risk factors to the burden of disease in Sweden. A comparison between Swedish and WHO data],” *Lakartidningen*, vol. 105, no. 11, pp. 816–821, Mar. 2008.

[5] S. Allender and M. Rayner, “The burden of overweight and obesity-related ill health in the UK,” *Obes. Rev. Off. J. Int. Assoc. Study Obes.*, vol. 8, no. 5, pp. 467–473, Sep. 2007, doi: 10.1111/j.1467-789X.2007.00394.x.

[6] S. Allender, R. Balakrishnan, P. Scarborough, P. Webster, and M. Rayner, “The burden of smoking-related ill health in the UK,” *Tob. Control*, vol. 18, no. 4, pp. 262–267, Aug. 2009, doi: 10.1136/tc.2008.026294.

[7] S. Allender, C. Foster, P. Scarborough, and M. Rayner, “The burden of physical activity-related ill health in the UK,” *J. Epidemiol. Community Health*, vol. 61, no. 4, pp. 344–348, Apr. 2007, doi: 10.1136/jech.2006.050807.

[8] S. S. Babatola, “Global burden of diseases attributable to air pollution,” *J. Public Health Afr.*, vol. 9, no. 3, p. 813, Dec. 2018, doi: 10.4081/jphia.2018.813.

[9] R. Balakrishnan, S. Allender, P. Scarborough, P. Webster, and M. Rayner, “The burden of alcohol-related ill health in the United Kingdom,” *J. Public Health Oxf. Engl.*, vol. 31, no. 3, pp. 366–373, Sep. 2009, doi: 10.1093/pubmed/fdp051.

[10] P. Begou, P. Kassomenos, and A. Kelessis, “Effects of road traffic noise on the prevalence of cardiovascular diseases: The case of Thessaloniki, Greece,” *Sci. Total Environ.*, vol. 703, p. 134477, Feb. 2020, doi: 10.1016/j.scitotenv.2019.134477.

[11] L. M. Bello, P. Saavedra, and L. Serra, “[Trends in mortality and years of life lost related to alcohol in the Canary Islands, Spain [1980-1998]],” *Gac. Sanit.*, vol. 17, no. 6, pp. 466–473, 2003, doi: 10.1016/s0213-9111(03)71793-2.

[12] D. A. Bennett *et al.*, “The global burden of ischemic stroke: findings of the GBD 2010 study,” *Glob. Heart*, vol. 9, no. 1, pp. 107–112, Mar. 2014, doi: 10.1016/j.gheart.2014.01.001.

[13] M. Borges, M. Gouveia, J. Costa, L. Dos Santos Pinheiro, S. Paulo, and A. Vaz Carneiro, “The burden of disease attributable to smoking in Portugal,” *Rev. Port. Pneumol.*, vol. 15, no. 6, pp. 951–1004, 2009.

[14] B. Bowe, Y. Xie, T. Li, Y. Yan, H. Xian, and Z. Al-Aly, “Estimates of the 2016 global burden of kidney disease attributable to ambient fine particulate matter air pollution,” *BMJ Open*, vol. 9, no. 5, p. e022450, May 2019, doi: 10.1136/bmjopen-2018-022450.

[15] E. W. Butt *et al.*, “Global and regional trends in particulate air pollution and attributable health burden over the past 50 years,” *Environ. Res. Lett.*, vol. 12, no. 10, p. 104017, Oct. 2017, doi: 10.1088/1748-9326/aa87be.

[16] G. Carreras *et al.*, “Burden of disease from breast cancer attributable to smoking and second-hand smoke exposure in Europe,” *Int. J. Cancer*, vol. 147, no. 9, pp. 2387–2393, Nov. 2020, doi: 10.1002/ijc.33021.

[17] A. J. Cohen *et al.*, “Estimates and 25-year trends of the global burden of disease attributable to ambient air pollution: an analysis of data from the Global Burden of Diseases Study 2015,” *Lancet Lond. Engl.*, vol. 389, no. 10082, pp. 1907–1918, May 2017, doi: 10.1016/S0140-6736(17)30505-6.

[18] GBD 2016 Risk Factors Collaborators, “Global, regional, and national comparative risk assessment of 84 behavioural, environmental and occupational, and metabolic risks or clusters of risks, 1990-2016: a systematic analysis for the Global Burden of Disease Study 2016,” *Lancet Lond. Engl.*, vol. 390, no. 10100, pp. 1345–1422, Sep. 2017, doi: 10.1016/S0140-6736(17)32366-8.

[19] GBD 2015 Risk Factors Collaborators, “Global, regional, and national comparative risk assessment of 79 behavioural, environmental and occupational, and metabolic risks or clusters of risks, 1990-2015: a systematic analysis for the Global Burden of Disease Study 2015,” *Lancet Lond. Engl.*, vol. 388, no. 10053, pp. 1659–1724, Oct. 2016, doi: 10.1016/S0140-6736(16)31679-8.

[20] H. Cortez-Pinto, M. Gouveia, L. dos Santos Pinheiro, J. Costa, M. Borges, and A. Vaz Carneiro, “The burden of disease and the cost of illness attributable to alcohol drinking--results of a national study,” *Alcohol. Clin. Exp. Res.*, vol. 34, no. 8, pp. 1442–1449, Aug. 2010, doi: 10.1111/j.1530-0277.2010.01229.x.

[21] J. De Oliveira Mota, G. Boué, S. Guillou, F. Pierre, and J.-M. Membré, “Estimation of the burden of disease attributable to red meat consumption in France: Influence on colorectal cancer and cardiovascular diseases,” *Food Chem. Toxicol. Int. J. Publ. Br. Ind. Biol. Res. Assoc.*, vol. 130, pp. 174–186, Aug. 2019, doi: 10.1016/j.fct.2019.05.023.

[22] L. Degenhardt *et al.*, “The global epidemiology and burden of psychostimulant dependence: findings from the Global Burden of Disease Study 2010,” *Drug Alcohol Depend.*, vol. 137, pp. 36–47, Apr. 2014, doi: 10.1016/j.drugalcdep.2013.12.025.

[23] L. Degenhardt *et al.*, “Estimating the burden of disease attributable to injecting drug use as a risk factor for HIV, hepatitis C, and hepatitis B: findings from the Global Burden of Disease Study 2013,” *Lancet Infect. Dis.*, vol. 16, no. 12, pp. 1385–1398, Dec. 2016, doi: 10.1016/S1473-3099(16)30325-5.

[24] L. Degenhardt *et al.*, “Global burden of disease attributable to illicit drug use and dependence: findings from the Global Burden of Disease Study 2010,” *Lancet Lond. Engl.*, vol. 382, no. 9904, pp. 1564–1574, Nov. 2013, doi: 10.1016/S0140-6736(13)61530-5.

[25] Direção-Geral da Saúde, Institute for Health Metrics and Evaluation., “Portugal: The Nation’s Health 1990–2016: An overview of the Global Burden of Disease Study 2016 Results.,” *Seattle, WA: IHME, 2018*, Apr. 04, 2018. https://www.healthdata.org/policy-report/portugal-nation%E2%80%99s-health-1990%E2%80%932016 (accessed Feb. 05, 2023).

[26] GBD 2016 Occupational Chronic Respiratory Risk Factors Collaborators and GBD 2016 occupational chronic respiratory risk factors collaborators, “Global and regional burden of chronic respiratory disease in 2016 arising from non-infectious airborne occupational exposures: a systematic analysis for the Global Burden of Disease Study 2016,” *Occup. Environ. Med.*, vol. 77, no. 3, pp. 142–150, Mar. 2020, doi: 10.1136/oemed-2019-106013.

[27] T. Driscoll *et al.*, “The global burden of disease due to occupational carcinogens,” *Am. J. Ind. Med.*, vol. 48, no. 6, pp. 419–431, Dec. 2005, doi: 10.1002/ajim.20209.

[28] A. Dzhambov and D. Dimitrova, “Road traffic noise and annoyance: exposure-response relationship and burden of disease calculations in Bulgaria,” *Scr. Sci. Medica*, vol. 47, no. 2, p. 22, Jun. 2015, doi: 10.14748/ssm.v47i2.1153.

[29] T. Effertz and K. Mann, “The burden and cost of disorders of the brain in Europe with the inclusion of harmful alcohol use and nicotine addiction,” *Eur. Neuropsychopharmacol. J. Eur. Coll. Neuropsychopharmacol.*, vol. 23, no. 7, pp. 742–748, Jul. 2013, doi: 10.1016/j.euroneuro.2012.07.010.

[30] M. Ezzati, A. D. Lopez, A. Rodgers, S. Vander Hoorn, C. J. L. Murray, and Comparative Risk Assessment Collaborating Group, “Selected major risk factors and global and regional burden of disease,” *Lancet Lond. Engl.*, vol. 360, no. 9343, pp. 1347–1360, Nov. 2002, doi: 10.1016/S0140-6736(02)11403-6.

[31] GBD 2015 Neurological Disorders Collaborator Group, “Global, regional, and national burden of neurological disorders during 1990-2015: a systematic analysis for the Global Burden of Disease Study 2015,” *Lancet Neurol.*, vol. 16, no. 11, pp. 877–897, Nov. 2017, doi: 10.1016/S1474-4422(17)30299-5.

[32] A. J. Ferrari *et al.*, “Burden of depressive disorders by country, sex, age, and year: findings from the global burden of disease study 2010,” *PLoS Med.*, vol. 10, no. 11, p. e1001547, Nov. 2013, doi: 10.1371/journal.pmed.1001547.

[33] A. J. Ferrari *et al.*, “The burden attributable to mental and substance use disorders as risk factors for suicide: findings from the Global Burden of Disease Study 2010,” *PloS One*, vol. 9, no. 4, p. e91936, 2014, doi: 10.1371/journal.pone.0091936.

[34] GBD 2013 Risk Factors Collaborators *et al.*, “Global, regional, and national comparative risk assessment of 79 behavioural, environmental and occupational, and metabolic risks or clusters of risks in 188 countries, 1990-2013: a systematic analysis for the Global Burden of Disease Study 2013,” *Lancet Lond. Engl.*, vol. 386, no. 10010, pp. 2287–2323, Dec. 2015, doi: 10.1016/S0140-6736(15)00128-2.

[35] GBD 2016 Alcohol and Drug Use Collaborators, “The global burden of disease attributable to alcohol and drug use in 195 countries and territories, 1990-2016: a systematic analysis for the Global Burden of Disease Study 2016,” *Lancet Psychiatry*, vol. 5, no. 12, pp. 987–1012, Dec. 2018, doi: 10.1016/S2215-0366(18)30337-7.

[36] GBD 2016 Occupational Carcinogens Collaborators, “Global and regional burden of cancer in 2016 arising from occupational exposure to selected carcinogens: a systematic analysis for the Global Burden of Disease Study 2016,” *Occup. Environ. Med.*, vol. 77, no. 3, pp. 151–159, Mar. 2020, doi: 10.1136/oemed-2019-106012.

[37] GBD 2017 Disease and Injury Incidence and Prevalence Collaborators, “Global, regional, and national incidence, prevalence, and years lived with disability for 354 diseases and injuries for 195 countries and territories, 1990-2017: a systematic analysis for the Global Burden of Disease Study 2017,” *Lancet Lond. Engl.*, vol. 392, no. 10159, pp. 1789–1858, Nov. 2018, doi: 10.1016/S0140-6736(18)32279-7.

[38] M. Gouveia, M. Borges, J. Costa, and A. V. Carneiro, “Burden of disease from hypercholesterolemia in Portugal,” *Rev. Port. Cardiol. Orgao Of. Soc. Port. Cardiol. Port. J. Cardiol. Off. J. Port. Soc. Cardiol.*, vol. 23, no. 2, pp. 255–270, Feb. 2004.

[39] P. Grandjean and M. Bellanger, “Calculation of the disease burden associated with environmental chemical exposures: application of toxicological information in health economic estimation,” *Environ. Health Glob. Access Sci. Source*, vol. 16, no. 1, p. 123, Dec. 2017, doi: 10.1186/s12940-017-0340-3.

[40] Y. Guillois-Becel, D. Eilstein, Ph. Glorennec, and A. Lefranc, “Quantification of years of life lost attributable to chronic air pollution exposure in a health impact assessment: the case of Nantes,” *Environ. Risques Sante*, vol. 6, no. 3, pp. 189–197, 2007.

[41] O. Hänninen *et al.*, “Environmental burden of disease in Europe: assessing nine risk factors in six countries,” *Environ. Health Perspect.*, vol. 122, no. 5, pp. 439–446, May 2014, doi: 10.1289/ehp.1206154.

[42] P. Holnicki, M. Tainio, A. Kałuszko, and Z. Nahorski, “Burden of Mortality and Disease Attributable to Multiple Air Pollutants in Warsaw, Poland,” *Int. J. Environ. Res. Public. Health*, vol. 14, no. 11, p. E1359, Nov. 2017, doi: 10.3390/ijerph14111359.

[43] L. S. Jakobsen, K. Granby, V. K. Knudsen, M. Nauta, S. M. Pires, and M. Poulsen, “Burden of disease of dietary exposure to acrylamide in Denmark,” *Food Chem. Toxicol. Int. J. Publ. Br. Ind. Biol. Res. Assoc.*, vol. 90, pp. 151–159, Apr. 2016, doi: 10.1016/j.fct.2016.01.021.

[44] D. Jarosińska, K. Polańska, B. Wojtyniak, and W. Hanke, “Towards estimating the burden of disease attributable to second-hand smoke exposure in Polish children,” *Int. J. Occup. Med. Environ. Health*, vol. 27, no. 1, pp. 38–49, Jan. 2014, doi: 10.2478/s13382-014-0223-6.

[45] P. A. Kassomenos, K. Dimitriou, and A. K. Paschalidou, “Human health damage caused by particulate matter PM10 and ozone in urban environments: the case of Athens, Greece,” *Environ. Monit. Assess.*, vol. 185, no. 8, pp. 6933–6942, Aug. 2013, doi: 10.1007/s10661-013-3076-8.

[46] M. D. Keall, D. Ormandy, and M. G. Baker, “Injuries associated with housing conditions in Europe: a burden of disease study based on 2004 injury data,” *Environ. Health Glob. Access Sci. Source*, vol. 10, p. 98, Nov. 2011, doi: 10.1186/1476-069X-10-98.

[47] K. Kellerborg, A.-K. Danielsson, P. Allebeck, M. M. Coates, and E. Agardh, “Disease burden attributed to alcohol: How methodological advances in the Global Burden of Disease 2013 study have changed the estimates in Sweden,” *Scand. J. Public Health*, vol. 44, no. 6, pp. 604–610, Aug. 2016, doi: 10.1177/1403494816653512.

[48] AB Knol and BAM Staatsen, “Trends in the environmental burden of disease in the Netherlands 1980 – 2020,” 500029001/2005. [Online]. Available: https://www.rivm.nl/bibliotheek/rapporten/500029001.pdf

[49] Nordic Burden of Disease Collaborators, “Life expectancy and disease burden in the Nordic countries: results from the Global Burden of Diseases, Injuries, and Risk Factors Study 2017,” *Lancet Public Health*, vol. 4, no. 12, pp. e658–e669, Dec. 2019, doi: 10.1016/S2468-2667(19)30224-5.

[50] Ann Kristin Knudsen, Jonas Minet Kinge, Vegard Skirbekk, and Stein Emil Vollset, “Sykdomsbyrde i Norge 1990–2013,” Bergen/Oslo: Folkehelseinstituttet, 2016, 2016:1. [Online]. Available: fhi.no/publ/2016/sykdomsbyrde-i-norge-1990-2013/#:~:text=Til%20tross%20for%20at%20befolkningen,leveår%20som%20følge%20av%20hjertesykdom.

[51] Knudsen AK, Tollånes MC, Haaland ØA, Kinge JM, Skirbekk V, Vollset SE, “Sykdomsbyrde i Norge 2015. Resultater fra Global Burden of Diseases, Injuries, and Risk Factors Study 2015 (GBD 2015),” Bergen/Oslo: Folkehelseinstituttet, 2017., Rapport 2017. [Online]. Available: https://www.fhi.no/publ/2017/sykdomsbyrde-i-norge-2015/#:~:text=Forventet%20levealder%20ved%20f%C3%B8dsel%20i,%2Dd%C3%B8delig%20helsetap%20(sykelighet).

[52] T. R. Ülikool, “Haiguskoormuse tõttu kaotatud eluaastad Eestis: seosed riskifaktoritega ja riskide vähendamise kulutõhusus,” 2004.

[53] C. M. M. Lawes, S. Vander Hoorn, M. R. Law, P. Elliott, S. MacMahon, and A. Rodgers, “Blood pressure and the global burden of disease 2000. Part II: estimates of attributable burden,” *J. Hypertens.*, vol. 24, no. 3, pp. 423–430, Mar. 2006, doi: 10.1097/01.hjh.0000209973.67746.f0.

[54] H. Lehtomäki *et al.*, “Health Impacts of Ambient Air Pollution in Finland,” *Int. J. Environ. Res. Public. Health*, vol. 15, no. 4, p. 736, Apr. 2018, doi: 10.3390/ijerph15040736.

[55] J. Lelieveld *et al.*, “Cardiovascular disease burden from ambient air pollution in Europe reassessed using novel hazard ratio functions,” *Eur. Heart J.*, vol. 40, no. 20, pp. 1590–1596, May 2019, doi: 10.1093/eurheartj/ehz135.

[56] X. Li, X. Cao, M. Guo, M. Xie, and X. Liu, “Trends and risk factors of mortality and disability adjusted life years for chronic respiratory diseases from 1990 to 2017: systematic analysis for the Global Burden of Disease Study 2017,” *BMJ*, vol. 368, p. m234, Feb. 2020, doi: 10.1136/bmj.m234.

[57] S. S. Lim *et al.*, “A comparative risk assessment of burden of disease and injury attributable to 67 risk factors and risk factor clusters in 21 regions, 1990-2010: a systematic analysis for the Global Burden of Disease Study 2010,” *Lancet Lond. Engl.*, vol. 380, no. 9859, pp. 2224–2260, Dec. 2012, doi: 10.1016/S0140-6736(12)61766-8.

[58] K. Lock, J. Pomerleau, L. Causer, D. R. Altmann, and M. McKee, “The global burden of disease attributable to low consumption of fruit and vegetables: implications for the global strategy on diet,” *Bull. World Health Organ.*, vol. 83, no. 2, pp. 100–108, Feb. 2005.

[59] A. D. Lopez, C. D. Mathers, M. Ezzati, D. T. Jamison, and C. J. L. Murray, “Global and regional burden of disease and risk factors, 2001: systematic analysis of population health data,” *Lancet Lond. Engl.*, vol. 367, no. 9524, pp. 1747–1757, May 2006, doi: 10.1016/S0140-6736(06)68770-9.

[60] S. Marmet, J. Rehm, and G. Gmel, “The importance of age groups in estimates of alcohol-attributable mortality: impact on trends in Switzerland between 1997 and 2011,” *Addict. Abingdon Engl.*, vol. 111, no. 2, pp. 255–262, Feb. 2016, doi: 10.1111/add.13164.

[61] J. J. Martín-Ramiro, E. Alvarez-Martín, and R. Gil-Prieto, “[Disability attributable to excess weight in Spain],” *Med. Clin. (Barc.)*, vol. 143, no. 4, pp. 150–156, Aug. 2014, doi: 10.1016/j.medcli.2013.05.028.

[62] A. M. May *et al.*, “The impact of a healthy lifestyle on Disability-Adjusted Life Years: a prospective cohort study,” *BMC Med.*, vol. 13, p. 39, Feb. 2015, doi: 10.1186/s12916-015-0287-6.

[63] T. Meier, P. Deumelandt, O. Christen, G. I. Stangl, K. Riedel, and M. Langer, “Global Burden of Sugar-Related Dental Diseases in 168 Countries and Corresponding Health Care Costs,” *J. Dent. Res.*, vol. 96, no. 8, pp. 845–854, Jul. 2017, doi: 10.1177/0022034517708315.

[64] T. Meier *et al.*, “Cardiovascular mortality attributable to dietary risk factors in 51 countries in the WHO European Region from 1990 to 2016: a systematic analysis of the Global Burden of Disease Study,” *Eur. J. Epidemiol.*, vol. 34, no. 1, pp. 37–55, Jan. 2019, doi: 10.1007/s10654-018-0473-x.

[65] H. Meijerink *et al.*, “Modelling the burden of hepatitis C infection among people who inject drugs in Norway, 1973-2030,” *BMC Infect. Dis.*, vol. 17, no. 1, p. 541, Aug. 2017, doi: 10.1186/s12879-017-2631-2.

[66] T. Miazgowski, A. Taszarek, K. Widecka, B. Miazgowski, and K. Homa, “Deaths, disability-adjusted life years and years of life lost due to elevated systolic blood pressure in Poland: estimates for the Global Burden of Disease Study 2016,” *Arter. Hypertens.*, vol. 22, no. 2, pp. 95–103, Jun. 2018, doi: 10.5603/AH.a2018.0005.

[67] A. H. Mokdad *et al.*, “Global burden of diseases, injuries, and risk factors for young people’s health during 1990-2013: a systematic analysis for the Global Burden of Disease Study 2013,” *Lancet Lond. Engl.*, vol. 387, no. 10036, pp. 2383–2401, Jun. 2016, doi: 10.1016/S0140-6736(16)00648-6.

[68] H. Möller, M. Dherani, C. Harwood, T. Kinsella, and D. Pope, “Health planning for the future: comparative risk assessment of five major lifestyle risk factors: evidence from the Wirral, UK,” *J. Public Health Oxf. Engl.*, vol. 34, no. 3, pp. 430–437, Aug. 2012, doi: 10.1093/pubmed/fds005.

[69] GBD 2017 Italy Collaborators, “Italy’s health performance, 1990-2017: findings from the Global Burden of Disease Study 2017,” *Lancet Public Health*, vol. 4, no. 12, pp. e645–e657, Dec. 2019, doi: 10.1016/S2468-2667(19)30189-6.

[70] A. Murphy *et al.*, “Ischaemic heart disease in the former Soviet Union 1990-2015 according to the Global Burden of Disease 2015 Study,” *Heart Br. Card. Soc.*, vol. 104, no. 1, pp. 58–66, Jan. 2018, doi: 10.1136/heartjnl-2016-311142.

[71] C. J. L. Murray *et al.*, “UK health performance: findings of the Global Burden of Disease Study 2010,” *Lancet Lond. Engl.*, vol. 381, no. 9871, pp. 997–1020, Mar. 2013, doi: 10.1016/S0140-6736(13)60355-4.

[72] J. N. Newton *et al.*, “Changes in health in England, with analysis by English regions and areas of deprivation, 1990-2013: a systematic analysis for the Global Burden of Disease Study 2013,” *Lancet Lond. Engl.*, vol. 386, no. 10010, pp. 2257–2274, Dec. 2015, doi: 10.1016/S0140-6736(15)00195-6.

[73] M. Oberg, M. S. Jaakkola, A. Woodward, A. Peruga, and A. Prüss-Ustün, “Worldwide burden of disease from exposure to second-hand smoke: a retrospective analysis of data from 192 countries,” *Lancet Lond. Engl.*, vol. 377, no. 9760, pp. 139–146, Jan. 2011, doi: 10.1016/S0140-6736(10)61388-8.

[74] M. Öberg, M. S. Jaakkola, A. Prüss-Üstün, A. Peruga, A. Woodward, and World Health Organization, “Global estimate of the burden of disease from second-hand smoke / by Mattias Öberg ... [et al],” 2010, [Online]. Available: https://apps.who.int/iris/handle/10665/44426

[75] S. Oberoi, B. Devleesschauwer, H. J. Gibb, and A. Barchowsky, “Global burden of cancer and coronary heart disease resulting from dietary exposure to arsenic, 2015,” *Environ. Res.*, vol. 171, pp. 185–192, Apr. 2019, doi: 10.1016/j.envres.2019.01.025.

[76] H. Orru *et al.*, “Health impact assessment of particulate pollution in Tallinn using fine spatial resolution and modeling techniques,” *Environ. Health Glob. Access Sci. Source*, vol. 8, p. 7, Mar. 2009, doi: 10.1186/1476-069X-8-7.

[77] M. C. Tollånes, A. K. Knudsen, S. E. Vollset, J. M. Kinge, V. Skirbekk, and S. Øverland, “Sykdomsbyrden i Norge i 2016,” *Tidsskr. Den Nor. Legeforening*, 2018, doi: 10.4045/tidsskr.18.0274.

[78] N. Papadimitriou *et al.*, “Burden of hip fracture using disability-adjusted life-years: a pooled analysis of prospective cohorts in the CHANCES consortium,” *Lancet Public Health*, vol. 2, no. 5, pp. e239–e246, May 2017, doi: 10.1016/S2468-2667(17)30046-4.

[79] K. Paunovic and G. Belojević, “Burden of myocardial infarction attributable to road-traffic noise: a pilot study in Belgrade,” *Noise Health*, vol. 16, no. 73, pp. 374–379, Dec. 2014, doi: 10.4103/1463-1741.144415.

[80] J. Pomerleau, K. Lock, and M. McKee, “The burden of cardiovascular disease and cancer attributable to low fruit and vegetable intake in the European Union: differences between old and new Member States,” *Public Health Nutr.*, vol. 9, no. 5, pp. 575–583, Aug. 2006, doi: 10.1079/phn2005910.

[81] J. W. Powles, W. Zatonski, S. Vander Hoorn, and M. Ezzati, “The contribution of leading diseases and risk factors to excess losses of healthy life in Eastern Europe: burden of disease study,” *BMC Public Health*, vol. 5, p. 116, Nov. 2005, doi: 10.1186/1471-2458-5-116.

[82] M. Rayner and P. Scarborough, “The burden of food related ill health in the UK,” *J. Epidemiol. Community Health*, vol. 59, no. 12, pp. 1054–1057, Dec. 2005, doi: 10.1136/jech.2005.036491.

[83] J. Rehm, K. D. Shield, M. X. Rehm, G. Gmel, and U. Frick, “Alcohol consumption, alcohol dependence, and attributable burden of disease in Europe: Potential gains from effective interventions for alcohol dependence,” 2012, doi: 10.5167/UZH-64919.

[84] J. Rehm, B. Taylor, M. Roerecke, and J. Patra, “Alcohol consumption and alcohol-attributable burden of disease in Switzerland, 2002,” *Int. J. Public Health*, vol. 52, no. 6, pp. 383–392, Dec. 2007, doi: 10.1007/s00038-007-7010-0.

[85] J. Rehm, J. Manthey, K. D. Shield, and C. Ferreira-Borges, “Trends in substance use and in the attributable burden of disease and mortality in the WHO European Region, 2010-16,” *Eur. J. Public Health*, vol. 29, no. 4, pp. 723–728, Aug. 2019, doi: 10.1093/eurpub/ckz064.

[86] J. Rehm, C. Mathers, S. Popova, M. Thavorncharoensap, Y. Teerawattananon, and J. Patra, “Global burden of disease and injury and economic cost attributable to alcohol use and alcohol-use disorders,” *Lancet Lond. Engl.*, vol. 373, no. 9682, pp. 2223–2233, Jun. 2009, doi: 10.1016/S0140-6736(09)60746-7.

[87] GBD 2015 Tobacco Collaborators, “Smoking prevalence and attributable disease burden in 195 countries and territories, 1990-2015: a systematic analysis from the Global Burden of Disease Study 2015,” *Lancet Lond. Engl.*, vol. 389, no. 10082, pp. 1885–1906, May 2017, doi: 10.1016/S0140-6736(17)30819-X.

[88] J. Rovira, J. L. Domingo, and M. Schuhmacher, “Air quality, health impacts and burden of disease due to air pollution (PM10, PM2.5, NO2 and O3): Application of AirQ+ model to the Camp de Tarragona County (Catalonia, Spain),” *Sci. Total Environ.*, vol. 703, p. 135538, Feb. 2020, doi: 10.1016/j.scitotenv.2019.135538.

[89] GBD 2017 Colorectal Cancer Collaborators, “The global, regional, and national burden of colorectal cancer and its attributable risk factors in 195 countries and territories, 1990-2017: a systematic analysis for the Global Burden of Disease Study 2017,” *Lancet Gastroenterol. Hepatol.*, vol. 4, no. 12, pp. 913–933, Dec. 2019, doi: 10.1016/S2468-1253(19)30345-0.

[90] M. Savolahti *et al.*, “Residential Wood Combustion in Finland: PM2.5 Emissions and Health Impacts with and without Abatement Measures,” *Int. J. Environ. Res. Public. Health*, vol. 16, no. 16, p. E2920, Aug. 2019, doi: 10.3390/ijerph16162920.

[91] L. Schwingshackl *et al.*, “Intake of 12 food groups and disability-adjusted life years from coronary heart disease, stroke, type 2 diabetes, and colorectal cancer in 16 European countries,” *Eur. J. Epidemiol.*, vol. 34, no. 8, pp. 765–775, Aug. 2019, doi: 10.1007/s10654-019-00523-4.

[92] K. D. Shield, J. Rehm, G. Gmel, M. X. Rehm, and A. Allamani, “Alcohol consumption, alcohol dependence, and related mortality in Italy in 2004: effects of treatment-based interventions on alcohol dependence,” *Subst. Abuse Treat. Prev. Policy*, vol. 8, p. 21, Jun. 2013, doi: 10.1186/1747-597X-8-21.

[93] K. D. Shield and J. Rehm, “Russia-specific relative risks and their effects on the estimated alcohol-attributable burden of disease,” *BMC Public Health*, vol. 15, p. 482, May 2015, doi: 10.1186/s12889-015-1818-y.

[94] K. Shield *et al.*, “National, regional, and global burdens of disease from 2000 to 2016 attributable to alcohol use: a comparative risk assessment study,” *Lancet Public Health*, vol. 5, no. 1, pp. e51–e61, Jan. 2020, doi: 10.1016/S2468-2667(19)30231-2.

[95] K. D. Shield, G. Gmel, J. Patra, and J. Rehm, “Global burden of injuries attributable to alcohol consumption in 2004: a novel way of calculating the burden of injuries attributable to alcohol consumption,” *Popul. Health Metr.*, vol. 10, no. 1, p. 9, May 2012, doi: 10.1186/1478-7954-10-9.

[96] K. Siddiqi *et al.*, “Global burden of disease due to smokeless tobacco consumption in adults: analysis of data from 113 countries,” *BMC Med.*, vol. 13, p. 194, Aug. 2015, doi: 10.1186/s12916-015-0424-2.

[97] D. Sifaki-Pistolla *et al.*, “Lung cancer and tobacco smoking in Crete, Greece: reflections from a population-based cancer registry from 1992 to 2013,” *Tob. Induc. Dis.*, vol. 15, p. 6, 2017, doi: 10.1186/s12971-017-0114-2.

[98] S. Sipetić *et al.*, “The burden of disease preventable by risk factor reduction in Serbia,” *Vojnosanit. Pregl.*, vol. 70, no. 5, pp. 445–451, May 2013, doi: 10.2298/vsp111024049s.

[99] GBD 2017 Disease and Injury Incidence and Prevalence Collaborators, “Global, regional, and national incidence, prevalence, and years lived with disability for 354 diseases and injuries for 195 countries and territories, 1990-2017: a systematic analysis for the Global Burden of Disease Study 2017,” *Lancet Lond. Engl.*, vol. 392, no. 10159, pp. 1789–1858, Nov. 2018, doi: 10.1016/S0140-6736(18)32279-7.

[100] N. Steel *et al.*, “Changes in health in the countries of the UK and 150 English Local Authority areas 1990-2016: a systematic analysis for the Global Burden of Disease Study 2016,” *Lancet Lond. Engl.*, vol. 392, no. 10158, pp. 1647–1661, Nov. 2018, doi: 10.1016/S0140-6736(18)32207-4.

[101] M. Tobollik, M. Hintzsche, J. Wothge, T. Myck, and D. Plass, “Burden of Disease Due to Traffic Noise in Germany,” *Int. J. Environ. Res. Public. Health*, vol. 16, no. 13, p. 2304, Jun. 2019, doi: 10.3390/ijerph16132304.

[102] E. Tod *et al.*, *Hospital admissions, deaths and overall burden of disease attributable to alcohol consumption in Scotland*. 2018.

[103] E. Tod *et al.*, “What causes the burden of stroke in Scotland? A comparative risk assessment approach linking the Scottish Health Survey to administrative health data,” *PloS One*, vol. 14, no. 7, p. e0216350, 2019, doi: 10.1371/journal.pone.0216350.

[104] GBD 2016 Lower Respiratory Infections Collaborators, “Estimates of the global, regional, and national morbidity, mortality, and aetiologies of lower respiratory infections in 195 countries, 1990-2016: a systematic analysis for the Global Burden of Disease Study 2016,” *Lancet Infect. Dis.*, vol. 18, no. 11, pp. 1191–1210, Nov. 2018, doi: 10.1016/S1473-3099(18)30310-4.

[105] K. K. Tsilidis *et al.*, “Burden of Cancer in a Large Consortium of Prospective Cohorts in Europe,” *J. Natl. Cancer Inst.*, vol. 108, no. 10, p. djw127, Oct. 2016, doi: 10.1093/jnci/djw127.

[106] Global Burden of Disease 2016 Greece Collaborators, “The burden of disease in Greece, health loss, risk factors, and health financing, 2000-16: an analysis of the Global Burden of Disease Study 2016,” *Lancet Public Health*, vol. 3, no. 8, pp. e395–e406, Aug. 2018, doi: 10.1016/S2468-2667(18)30130-0.

[107] F. Valent, D. Little, R. Bertollini, L. E. Nemer, F. Barbone, and G. Tamburlini, “Burden of disease attributable to selected environmental factors and injury among children and adolescents in Europe,” *Lancet Lond. Engl.*, vol. 363, no. 9426, pp. 2032–2039, Jun. 2004, doi: 10.1016/S0140-6736(04)16452-0.

[108] K. van, K. AGAC, R. van, and CVG, “Our food, our health-Healthy diet and safe food in the Netherlands,” Jan. 2006.

[109] D. Vienneau *et al.*, “Years of life lost and morbidity cases attributable to transportation noise and air pollution: A comparative health risk assessment for Switzerland in 2010,” *Int. J. Hyg. Environ. Health*, vol. 218, no. 6, pp. 514–521, Aug. 2015, doi: 10.1016/j.ijheh.2015.05.003.

[110] H. A. Whiteford *et al.*, “Global burden of disease attributable to mental and substance use disorders: findings from the Global Burden of Disease Study 2010,” *Lancet Lond. Engl.*, vol. 382, no. 9904, pp. 1575–1586, Nov. 2013, doi: 10.1016/S0140-6736(13)61611-6.

[111] World Health Organization, “The global burden of disease : 2004 update,” World Health Organization, 2008. Accessed: Feb. 05, 2023. [Online]. Available: https://apps.who.int/iris/handle/10665/43942
